# “Bligh and Dyer” and Folch Methods for Solid–Liquid–Liquid Extraction of Lipids from Microorganisms. Comprehension of Solvatation Mechanisms and towards Substitution with Alternative Solvents

**DOI:** 10.3390/ijms18040708

**Published:** 2017-03-27

**Authors:** Cassandra Breil, Maryline Abert Vian, Thomas Zemb, Werner Kunz, Farid Chemat

**Affiliations:** 1 GREEN (Groupe de Recherche en Eco-Extraction de Produits Naturels), Université d’Avignon et des Pays du Vaucluse, INRA, UMR 408, GREEN, F-84000 Avignon, France; cassandra.breil@alumni.univ-avignon.fr (C.B.); maryline.vian@univ-avignon.fr (M.A.V.); 2 Institut de Chimie Séparative de Marcoule, F-30207 Bagnols Sur Cèze, France; thomas.zemb@icsm.fr; 3 Institute of Physical and Theoretical Chemistry, University of Regensburg, D-93040 Regensburg, Germany; werner.kunz@chemie.uni-regensburg.de

**Keywords:** Bligh and Dyer, Folch, bio-sourced solvent, yeast, lipids

## Abstract

Bligh and Dyer (B & D) or Folch procedures for the extraction and separation of lipids from microorganisms and biological tissues using chloroform/methanol/water have been used tens of thousands of times and are “gold standards” for the analysis of extracted lipids. Based on the Conductor-like Screening MOdel for realistic Solvatation (COSMO-RS), we select ethanol and ethyl acetate as being potentially suitable for the substitution of methanol and chloroform. We confirm this by performing solid–liquid extraction of yeast (*Yarrowia lipolytica IFP29*) and subsequent liquid–liquid partition—the two steps of routine extraction. For this purpose, we consider similar points in the ternary phase diagrams of water/methanol/chloroform and water/ethanol/ethyl acetate, both in the monophasic mixtures and in the liquid–liquid miscibility gap. Based on high performance thin-layer chromatography (HPTLC) to obtain the distribution of lipids classes, and gas chromatography coupled with a flame ionisation detector (GC/FID) to obtain fatty acid profiles, this greener solvents pair is found to be almost as effective as the classic methanol–chloroform couple in terms of efficiency and selectivity of lipids and non-lipid material. Moreover, using these bio-sourced solvents as an alternative system is shown to be as effective as the classical system in terms of the yield of lipids extracted from microorganism tissues, independently of their apparent hydrophilicity.

## 1. Introduction

Lipids from microorganisms’ matrices such as microalgae or yeasts are hydrophobic molecules which are soluble in many organic solvents. They can be divided according to the polarity of their head groups: neutral lipids (acylglycerols, free fatty acids, sterols, sterols esters, waxes and hydrophobic pigments) [1] which are synthetized by the cells to store energy, and polar lipids (phospholipids, glycolipids, polysaccharides and proteins) which are the matrix of the cellular membrane. Estimation of the total lipids content in a microorganism sample is crucial for various kinds of applications such as biodiesel [2], nutritional supplements [3], cosmetic [4], etc.

The safest and nowadays most popular way to ensure that all of the cellular lipids are extracted is to employ a ternary solvent composition including a polar as well as a non-polar solvent. The first step is a solid–liquid extraction from an initial amorphous gel state, followed by a second liquid–liquid partitioning step. In the second step, the initially extracted mixture of all biomolecules is separated into two phases: an organic-rich phase (OR) containing the total lipids and a water-rich phase (WR) containing others compounds (sugars, proteins, etc.).

The standard of these solvent mixtures is chloroform–methanol, described for the first time more than 50 years ago by Folch [5,6] and Bligh and Dyer [7] (B & D) and cited more than 50,000 times in the “experimental” section of papers describing the analytics of lipids and proteins extracted and separated with high efficiency form micro-organisms.

The crucial point in the second step is the partial miscibility of the chloroform in the WR phase and the water in the OR phase [8]. This ensures that all biomolecules, independent of their volume and “hydrophilicity”, are solubilized either in the OR or in WR coexisting phases.

The sample of biological origin is mixed with water, methanol and chloroform in solvent ratios to form a monophasic ternary solvent mixture. In this region of the phase diagram, no structuring appears and predictions of models based on random phase approximation such as COSMO-RS are expected to be good. By adding a sufficient amount of water, a biphasic system is then spontaneously formed leading to the partitioning of proteins [9], carbohydrates [10] and phospholipids [11] into the WR-rich upper-layer (essentially water–methanol) and non-polar components such as most lipids into a hydrophobic, chloroform-rich lower layer [12]. These two layers are in equilibrium via exchange through the meniscus, i.e., water, chloroform as well as ethanol chemical potentials are the same in the two phases. Both layers are nanostructured close to a critical point. Further, the WR layer requires less cost in free energy to insert proteins, while the OR layer is an efficient host for all types of lipids present, independent of their intrinsic hydrophilicity.

These above-mentioned gravimetric methods are still widely used for the estimation of lipids in hospital, pharmaceutical, food, or biofuel laboratories [13]. However, these methods have serious disadvantages in terms of safety, especially with chloroform, which is highly toxic and carcinogenic [11], making it inappropriate for large-scale application.

In order to improve these basic methods of Folch or B & D, many researchers adopted modifications concerning the substitution of hazardous solvents. Atsushi et al. were the first to substitute the chloroform–methanol system by hexane–isopropanol mixtures [14]. Despite this initial breakthrough, the results showed a lower efficiency. Several years later, more studies have been pursued by several teams such as Molina Grima [15], Lee [16], Sheng [17], Caprioli [18] and many others, that have shown the effectiveness of many promising solvents to replace the chloroform–methanol mixture. However, these methods still remain toxic to human health and the environment. Nowadays, scientific and industrial research laboratories face the challenge of finding an appropriate extraction method with minimum energy consumption and greener solvents [19] such as the last work of the research laboratory of Wallenberg using heptane and ethyl acetate as alternative solvents [20]. Since the Folch as well as the Bligh and Dyer methods for total determination of lipids were published, a large number of publications appeared showing modifications or intensifications of both methods. They have been reviewed by Iverson and coworkers [21]. Among them, the use of ultrasound [1,22,23,24], microwaves [16,22,23,25,26], heat [25,27], pressure [27,28] or beads [24,27] were often raised to improve the efficiency of lipid recovery from various tissues such as bacteria [23], yeasts [29] or microalgae [30] (supplementary material). However, all these methods were using solvents of fossil origin issued from petrol.

From the point of view of environmental protection and the development of green chemistry, toxic petroleum solvents will have to be replaced in the future by bio-sourced solvents (or “bio-solvents”) [31].

In this paper, we compare the performances of several alternative solvents to substitute chloroform–methanol mixtures used in the Folch and B & D protocols for the extraction of *Yarrowia lipolytica* (*Y.L*), a well-known oleaginous organism proven to be suitable for many different industrial processes such as the production of biodiesel fuel, functional fatty acids and carotenoids. First, the theoretical approach of the conductor-like screening model for realistic solvation (COSMO-RS) was used to simulate the relative solubility of the solutes (free fatty acids (FFAs), diglycerides (DAGs), triglycerides (TAGs), phospholipids (PLs), proteins, polysaccharides, glucose, amino acids and sterols from the microorganism sample) in several selected alternative solvents: 2-methyltetrahydrofuran (MeTHF), cyclopentyl methyl ether (CPME), ethyl acetate (EtOAc), ethyl lactate, dimethyl carbonate (DMC), *p*-cymene, *d*-limonene and α-pinene as potential substitutes of chloroform; and ethanol (EtOH) and isopropanol (IPA) to substitute methanol.

In the course of further selection of solvents, we keep the common strategy of Folch and B & D protocols which are based on two-step solid/liquid and then liquid/liquid extraction methods with partition of hydrophobic lipids to an organic solvent-rich ternary fluid phase, while unwanted compounds are partitioned in the water-rich phase. Lipids extracted in both phases are analyzed by high thin layer chromatography (HPTLC) to obtain quantities of extracted lipids by lipid classes and by gas chromatography coupled with a flame ionization detector (GC/FID) in order to obtain the fatty acid profiles. Sugars and proteins are quantified by the UV spectrometry method. 

## 2. Results and Discussion

### 2.1. Bligh and Dyer: Principle

The B & D method has been considered as the standard method for the determination of total lipids in biological tissues such as microorganisms. Methanol, chloroform and water are added to the sample in a two-step extraction and, after phase separation, lipids are quantified in the chloroform phase.

The compounds with a known amount of water (80%) are dissolved in this binary system and are then separated by transition from the monophasic system to the biphasic system induced by the addition of water: the final composition is located inside the miscibility gap: two samples with compositions given by the points at the end of the tie-line coexist [32]. The partition of compounds between the WR and the OR phases can be estimated in a first approximation by Hansen’s solubility parameters as a roughly quantitative description of “like dissolves like”; the proteins and sugars are preferably partitioned into the WR layer and lipids into the OR layer. The adding of potassium chloride in the separation step can modify the distribution of lipids between the two phases and is sometimes considered as a substance that favours lipid exchange between the aqueous phase and the organic phase. This phenomenon is supposed to be due to cations generated by the salts (KCl) which decreased the dissociation of lipids by a mass action effect, which, therefore, shift lipids to the OR phase, keeping salts in the WR phase [6]. It can be also considered as a typical salting-out effect. 

Nowadays, the use of hazardous and toxic solvents such as chloroform and methanol in the chemical sector (laboratories and industry) is considered as a very important problem for the health and safety of workers and environmental concerns. The green chemistry approach aims to substitute toxic solvents by greener alternatives. In the case of extraction, chlorinated solvents and methanol are two typical examples of such problematic solvents. Consequently, investigations of alternative solvents have been done following the same principles as for conventional B & D extraction.

### 2.2. Strategy for Selection of Bio-Sourced Solvents: COSMO-RS Approach

In the present case, *Y.L* yeast biomass was used as the lipid matrix. Thus, molecules synthetized by *Y.L* IFP29 were determined thanks to a preliminary study [11] performed with the same yeast. A simulation with this software was conducted to determine the solubility of the synthetized molecules by yeasts such as free fatty acids (oleic acid, linoleic acid, stearic acid), triglycerides, diglycerides, polar lipids (phosphatidylethanolamine, phosphatidylcholine) [11], sterols (lanosterol and ergosterol) [13], polysaccharides (1,3 bd glucan, 1,4 bd glucan and chitin) [33,34], amino acids (arginine and histidine) and sugars (glucose), in the collection of solvents considered. Regarding the results given by COSMO-RS predictions of convenient solvents, two of them were selected: isopropanol and ethanol. Bio-isopropanol derived from *E. coli* bacteria via fermentation [35], allows the solubilization of almost all kinds of model compounds in contrast to ethanol, which is selective only towards polar lipids and sterols, polysaccharides, glycerol and amino acids. Moreover, ethanol can be obtained from agricultural resources via fermentation thanks to many bacteria [36,37,38].

Eight alternative solvents were selected to replace chloroform: ethyl acetate, 2-methyltetrahydrofuran (MeTHF), cyclopenthylmethylether (CPME), dimethylcarbonate (DMC), ethyl lactate, α-pinene, *d*-limonene and *p*-cymene. A priori, the best candidate should have polarity properties similar to chloroform. However, several additional properties have to be considered further to solubility, such as volatility, viscosity, energy required for elimination [39] and ability to form a two-phase system with water. According to these requirements, ethyl acetate, MeTHF and CPME turned out to be the most appropriate alternative solvents. However, although CPME can be considered as a “greener” solvent compared to chloroform, because it is in agreement with green chemistry principles 1, 5 and 12 [40], it is not bio-sourced [41]. Ethyl acetate and MeTHF forming a ternary system with water, are able to create a biphasic system under specific conditions and are considered as being bio-sourced solvents. According to COSMO-RS simulations, shown in Table 1, MeTHF is not selective enough for proteins, polysaccharides and glucose. So, according to this theoretical work, ethyl acetate was considered to be the most appropriate solvent to replace chloroform.

### 2.3. Partition of Macro-Constituents from Y.L Yeast into Pure Solvents

In this part, the solubility of constituents from *Y.L* yeast such as lipids, proteins and sugars was studied in each pure solvent of the B & D procedure and bio-sourced solvents as pre-selected based on the computational study with COSMO-RS: ethyl acetate and ethanol.

**Figure 1:**
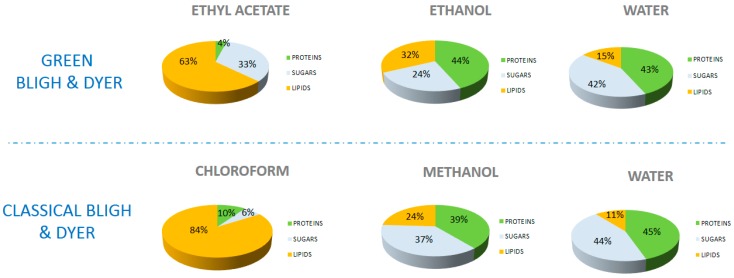
Initial test of solubilities in pure solvents. Relative distribution (weight) of different extracted compounds (lipids, proteins and sugars) in the pure solvents.

According to Figure 1, chloroform and ethyl acetate phases contain mainly lipids (respectively 84% and 63%) and solubilize, in smaller amounts, proteins (4% and 10%) and sugars (6% and 33%). These results show that chloroform is more selective towards lipids than ethyl acetate.

Ethanol and methanol are respectively mixed in the aqueous phases; they solubilize sugars (respectively, 24% and 37%), proteins (44% and 39%) and lipids (32% and 24%) such as polar lipids and free fatty acids. The solubility of lipids in methanol or ethanol is lower than in chloroform or in ethyl acetate, but higher for glucose and proteins.

Water extracts about 43% of proteins, 44% of glucose and 13% of lipids (being nonpolar, lipids are hardly solubilized). 

These results are in good agreement with the “like dissolves like” empirical rule. It is based on the polarity of the systems; polar molecules dissolve in polar solvents (alcohol, water) and non-polar solvent molecules in non-polar solvents. 

As shown in the Table 1, no significant differences exist between the theoretical predictions and the experimental results. In both cases, ethyl acetate and chloroform have a high solubility power for lipids. Moreover, ethyl acetate extracts glucose (33%), which was also predicted by COSMO-RS. According to simulations, ethanol and methanol are less efficient than other solvents (chloroform and ethyl acetate) to solubilize lipids, which was experimentally confirmed by lower lipid extraction yields. Concerning water, the experimental results confirm the expected high solubility of proteins and glucose, which is also consistent with the predicted COSMO-RS values. Furthermore, as also expected and predicted, water solubilizes 250 times less lipids than the others solvents investigated.

### 2.4. Evaluation of Lipids Extraction from Y.L with B & D Solvent Pair versus the Alternative Solvent Pair

*Yarrowia lipolytica* yeast was extracted in two different ways: with chloroform–methanol–water which is the classical B & D method, and with ethyl acetate/ethanol/water which is the alternative mixture with different compositions (shown in ternary diagram with blue points).

The purpose of this part is to define the best proportion of mixtures which can extract all lipids. Therefore, three types of extractions were carried out. The first type of extraction was done with wet yeast from the first stage, the second one was performed with dried yeast and the third extraction was performed directly with compositions located in the miscibility gap of the ternary diagram (12′, 13′ and 14′ for the classical system and L, M and N for the alternative system).

#### 2.4.1. Evaluation of Total Lipid Contents

The results obtained by gas chromatography give the lipid content in only the OR phase, while the gravimetric method gives the overall content of the extract (proteins, lipids, sugars …). The results obtained by the two methods can be compared to define the purity (only lipids) of the extract. According to our results, the amounts detected by the gravimetric method and gas chromatography are equivalent. As a consequence, the organic extracts found in the OR phase are almost pure lipids, therefore both ethyl acetate and chloroform are sufficiently selective to ensure reliable analytics.

**Figure 2:**
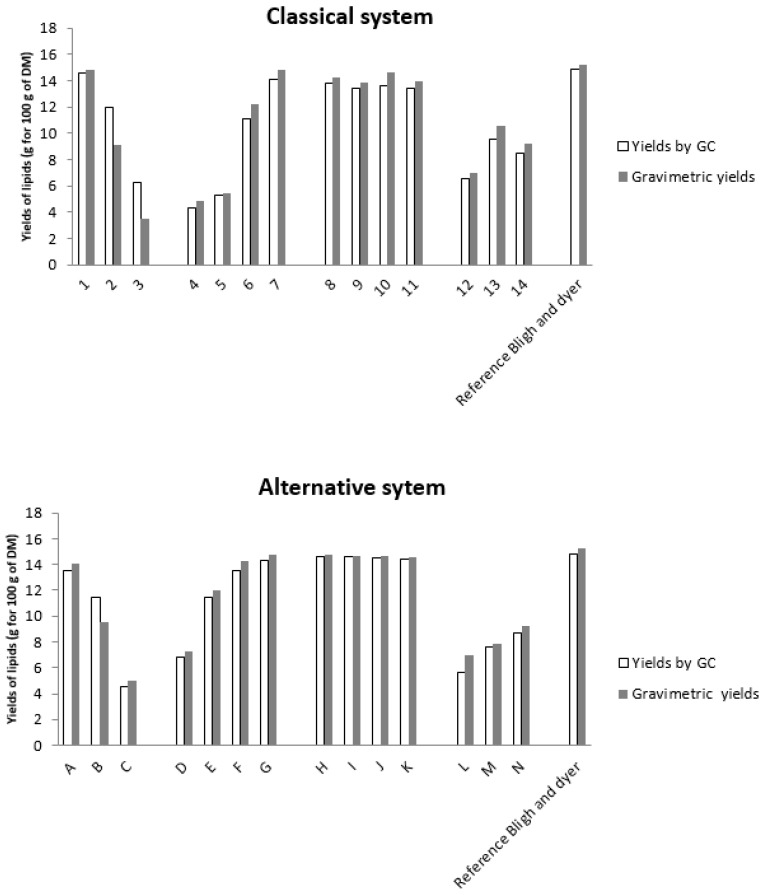
Extraction yields (g for 100 g of dry matter (DM)): Results of different extractions realized with the chloroform–methanol–water system and with the ethyl acetate–ethanol–water system, given by the gravimetric method and gas chromatography compared to the Bligh and Dyer reference method (1959).

The effect on *Yarrowia lipolytica* lipid extraction is shown in Figure 2 and Table 2 and Table 3. The highest yield was 14.85%, as obtained with the classical method of B & D with a ratio 2:1 methanol:chloroform (*v*:*v*), although other compositions (points 8, 9, G, H, J, K) show comparable yields. When the percentage of water increases, yields decrease (see points 4–7). The highest yield is for the composition of point 7 (14.12%) with a ratio of chloroform/methanol/water of 56.25/37.5/6.25. For the extraction with dry yeast (point 8–11), the yields are similar; the best ratio was 62.5% of chloroform to extract almost all lipids present in the biomass.

On the other hand, biphasic systems are not efficient; this may be explained by the heterogeneity of the mixture, which obviously has a low extraction power of molecules. 

Regarding the experimental results with the greener system, the yield for the composition of point G was 14.35% with a ratio of 67/30/3 in ethyl acetate/ethanol/water (2/1/0.08 *v*/*v*/*v*). Similar to the classical system, the yields for points I to J were almost similar, so a ratio of 75/20/0 of ethyl acetate/ethanol/water was enough to extract 83% of all lipids. Biphasic systems extract only a maximum of 59% of total lipids. These biphasic systems are not optimized for a solid–liquid extraction.

The fatty acids profiles obtained with both systems are similar. Oleic acid (C18:1), linoleic acid (C18:2n6) and palmitic acid (C16) were mainly present in microbial oil and represented at least 90% of the total extract (respectively 50% of C18:1, 30% of C18:2n6 and 10% of C16:0). Palmitoleic acid (C16:1) and stearic acids were present in minor amounts. The detailed composition for each extract is reported in Table 2 and Table 3.

Diphasic systems are inefficient in the solid–liquid solubilization step while they are more efficient in the second step for liquid–liquid partition. This basic principle is common to Bligh and Dyer and to the alternative safe solvent mixtures of ethanol/ethyl and acetate/water that we propose here.

#### 2.4.2. Lipid Classes of *Y.L* Yeast

**Figure 3:**
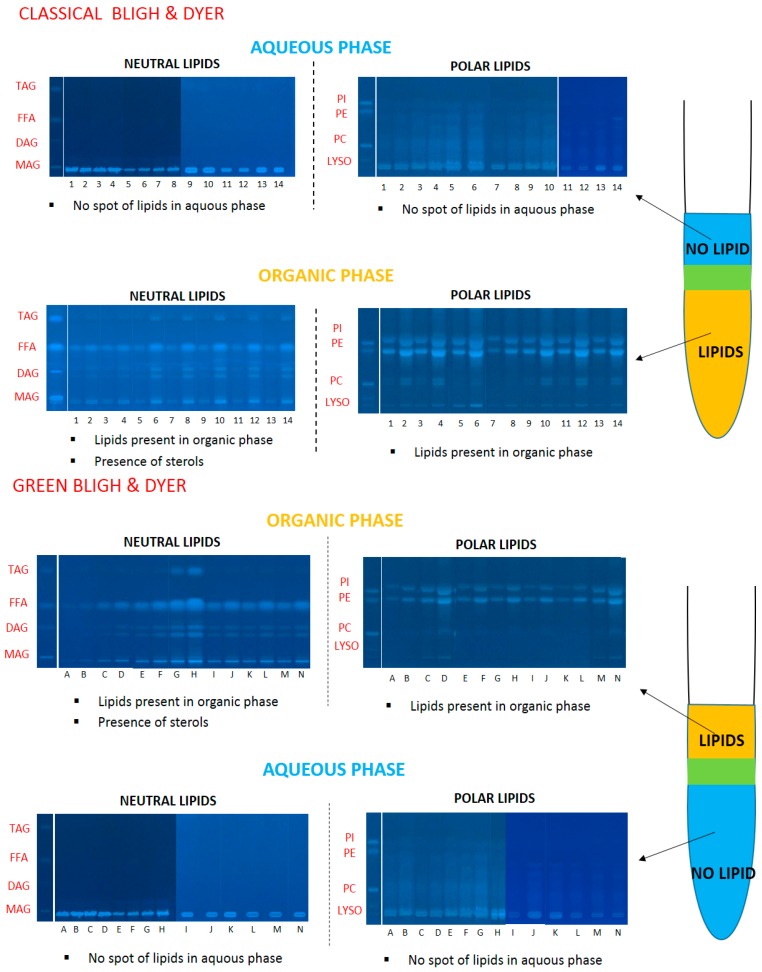
High performance thin-layer chromatography (HPTLC) plates of classical and alternative systems; validation of methods by the presence of lipids in organic phases. (PI: phosphatidylinositol, PE: phosphatidylethanolamine, PC: phosphatitylcholine, Lyso: lysophosphatidylcholine, FFA: Free Fatty Acids, DAG: diacylglycerol, TAG: Triacylglycerol, MAG: monoacylglycerol).

**Figure 4:**
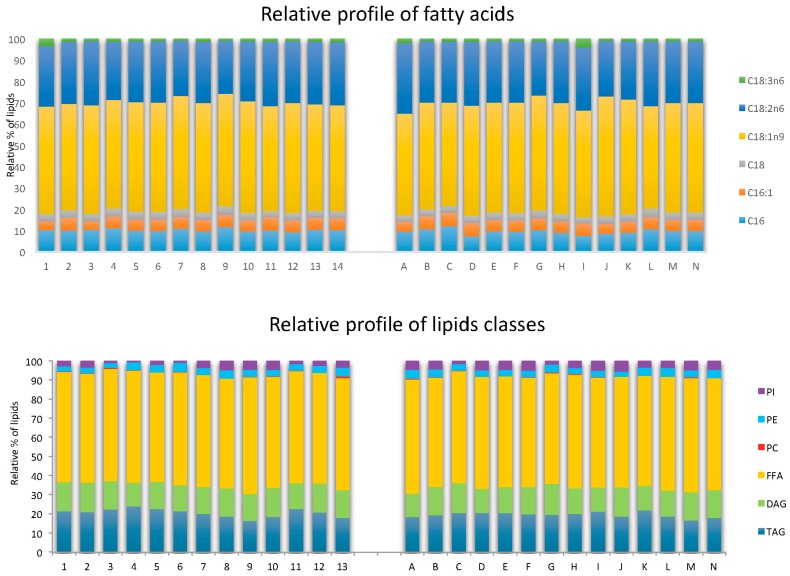
Relative profiles of fatty acids and lipid classes given by gas chromatography and HPTLC. The aim was to determine whether there was selectivity of different lipid classes (PI: phosphatydilinositol, PE: phosphatidylchoine, PC: phosphatitylcholine, FFA: Free Fatty Acids, DAG: diacylglycerol, TAG: Triacylglycerol) and fatty acids (C16: palmitic acid, C16:1: hexadecanoic acid, C18: stearic acid, C18:1n9: oleic acid, C18:2n6: linoleic acid, C18:3n6 : linolenic acid) between the different extraction compositions of points 1 to 13 in a classical diagram and A to N in an alternative diagram.

With the HPTLC technique, lipids can be detected qualitatively and quantitatively in each phase. From HPTLC plates in Figure 3 and Figure 4 and Table 2 and Table 3, we deduced the main classes of lipids generated by the yeast: triglycerides (about 20%), diglycerides (about 15%), free fatty acids (about 58%), phosphatidylcholine (about 0.2%), phosphatidylethanolamine (about 3.8%) and phosphatidylinositol (3%).

For the classical system with chloroform–methanol–water, all classes of lipids (neutral and polar) are visible on the plates. Moreover, a significant amount of sterols, not quantified by this method, was present. Conversely, in aqueous phases, lipids were not or not clearly observable in Figure 4; this means that the aqueous phases do not significantly solubilize lipids.

Similarly, in the greener system with ethyl acetate–ethanol–water, lipids are extracted by the OR phase and found only in negligible amounts in the WR phase.

To conclude, with this analytical method, it was shown that both ternary solvent mixtures are effective to extract lipids, and have a high selectivity when the monophasic system is converted to biphasic systems. Even if there are traces in the aqueous phase, the two systems are appropriate for the recovery of lipids.

#### 2.4.3. Determination of Proteins and Sugars in Both Systems

**Figure 5:**
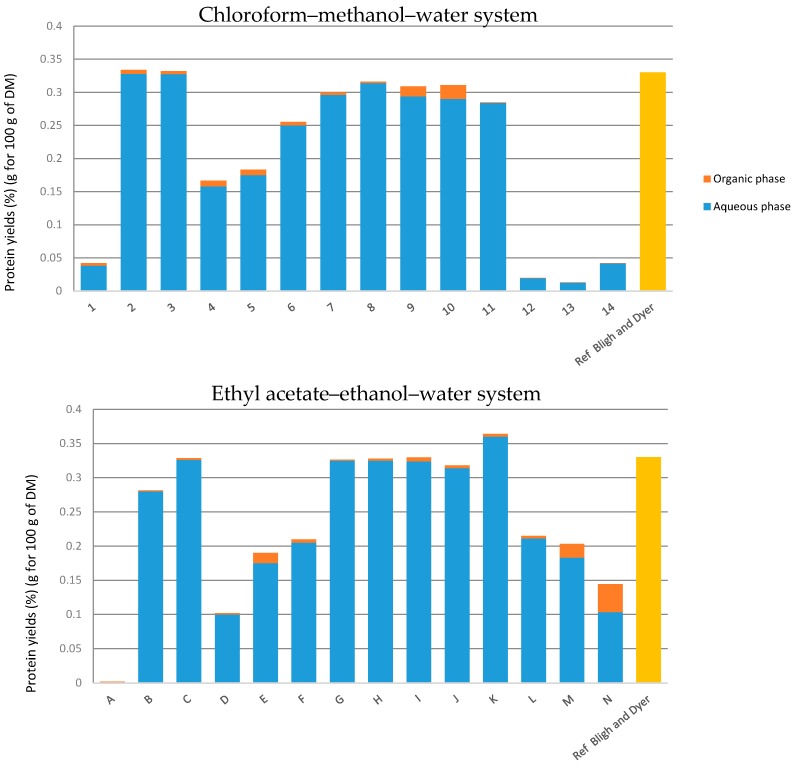
Extraction yields of proteins in both systems. Yields obtained by UV spectrometry in aqueous and organic phases. Points 1 to 13 are in the diagram with the classical solvents and points A to N are in the diagram with the green alternative solvents. The orange part is the organic phase and the blue part is the aqueous phase.

In terms of selectivity, water-soluble proteins possess a great affinity to the aqueous phase (points 2 and 3 or B and C in Figure 5). Points 1 and A, representing extractions exclusively with chloroform and ethyl acetate, show that both these solvents have low extraction powers for proteins. Yields are higher for the points 4, 5, 6 and 7 for the chloroform–methanol–water system and D, E, F and G for the ethyl acetate–ethanol–water system. The restructuring of cell walls is greater when the amount of chloroform or ethyl acetate increases. Regarding both systems, it is noted that results of extraction yields follow the same trend: extractions made directly in the biphasic area of the diagram are ineffective and the extraction of proteins is total from point 8 to 11 for the classical system and G to K for the alternative system.

**Figure 6:**
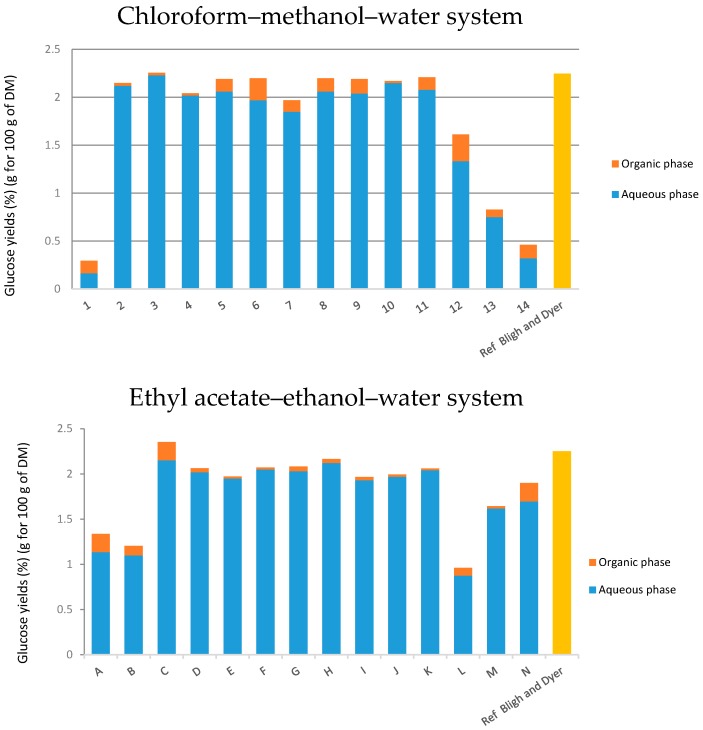
Extraction yields of glucose in both systems. Yields obtained by UV spectrometry in aqueous and organic phases.

The glucose extraction yields of various mixtures are presented in Table 2 and Table 3 and Figure 6. It can be noted that glucose yields are almost similar for points 4–11 for the classical system and D to K for the new greener system. Hypothetically, that means that the glucose was a component mainly present in the substrate, which the yeast did not entirely consume during the cultivation time. It can also be noticed that the amount of glucose contained in the organic phase is minimal. On the other hand, the amount of sugars in the aqueous phase is significant. Its solubility in water and methanol/ethanol is higher than in the organic phase composed of chloroform or ethyl acetate. Points 12, 13 and 14 or L, M and N show that extractions performed in the biphasic area of the phase diagram were not effective, probably because of the heterogeneity of the system.

### 2.5. COSMO-RS Calculations: Comparison of Experimental and Theoretical Approach

Using data shown in Table 1, we can now compare experimentally determined yields with COSMO-RS calculations of the ethyl acetate–ethanol–water system used in the previous study. All compositions tested in the experimental part (with or without water and the biphasic system) were modelled with COSMO-RS.

The aim of this study is to find the most efficient monophasic mixture for solubilizing all compounds from yeast. According to experiments of monophasic mixtures containing water, the composition denoted with G in Figure 3 is the best system to extract lipids with a yield of 14.35%, i.e., 96% of total lipids. Regarding the COSMO-RS results (Table 4), the system D is also the best for the solubilization of all the compounds synthesized by the yeast. The theory is consistent with the experiment: monophasic system G is selected as being the best combination of solvents. This system is effective because the amount of ethyl acetate present is more important than in other mixtures (67%).

Regarding greener systems which do not contain water (Table 4), systems H, I, J, and K are equivalent in terms of solubility of various compounds. Considering these theoretical and experimental approaches, the combination of solvent H (70/30 in ethyl acetate/ethanol) would be as efficient as mixtures I, J or K.

Concerning the simulations of extraction realized directly in the biphasic area of the diagram, the compositions given by the points L and M are less efficient than that given by point N. The latter is considered to be the best system compared to others from the viewpoint of solvation.

From a practical point of view, solid–liquid extractions with biphasic mixtures are not appropriate because they do not form a homogeneous mixture when they are in contact with the tissue. If the polar solvent (in this case ethanol) is more in contact with the biomass than the apolar solvent (ethyl acetate), extraction of lipids would not be effective.

## 3. Material and Methods

### 3.1. Computational Method: Theoretical Prediction with COSMO-RS

The principle of the theoretical procedure using COSMO-RS for solvent–solute interactions has been explained and detailed in our previous publication: Breil et al. [11].

In this work, we use the COSMO-RS approach to derive the chemical potential of a substance in the liquid solvent [42]. Calculations of the relative solubility of typical proteins and sugars, but also TAGs, DAGs, MAGs, fatty acids FFAs and PLs of microbial oil in various solvents were done by implementing this COSMO-RS model on COSMOtherm software (C30 1401, CosmothermX14, COSMOlogic GmbH & Co, KG, Cosmologic, Leverkusen, Germany). Relative solubility is calculated from COSMOlogic (GmbH & Co, KG, 2013, Cosmologic, Leverkusen, Germany) with the help of the following equation:

(1) $$\log_{10}(x_{j})=\log_{10}\left[\frac{\exp\left(\mu_{j}^{\mathrm{pure}}-\mu_{j}^{\mathrm{solvent}}-\Delta G_{j.\mathrm{fusion}}\right)}{RT}\right].$$

$\mu_{j}^{\mathrm{pure}}:\;\mathrm{chemical}\;\mathrm{potential}\;\mathrm{of}\;\mathrm{pure}\;\mathrm{compound}\;j\;\left(\mathrm{Joule}/\mathrm{mol}\right)$
$\mu_{j}^{\mathrm{solvent}}:\;\mathrm{chemical}\;\mathrm{potential}\;\mathrm{of}\;j\;\mathrm{at}\;\mathrm{infinite}\;\mathrm{dilution}\;\left(\mathrm{Joule}/\mathrm{mol}\right)$
$\Delta G_{j.\mathrm{fusion}}:\;\mathrm{free}\;\mathrm{energy}\;\mathrm{of}\;\mathrm{fusion}\;\mathrm{of}\;j\;\left(\mathrm{Joule}/\mathrm{mol}\right)$
$x_{j}:\;\mathrm{solubility}\;j\;\left(g/g\;\mathrm{solvent}\right)$

Relative solubility is always calculated in infinite dilution. The logarithm of the best sobility is set to 0, and all other solvents are given relatively to the best solvent. A solvent with a log_10_(*x*_j_) value of −1.00 yields a solubility that is lower by a factor of 10 compared to the best solvents [39,43].

### 3.2. Strain, Culture and Harvesting Conditions

The strain, culture and harvesting conditions of *Yarrowia lipolitica IFP29* have been detailed in our previous work: Meullemiestre et al. [29]. 

### 3.3. Chemicals

Hexane, ethanol >99.99%, ethyl acetate, all analytical grade, were obtained from VWR International (Darmstadt, Germany). Methanol, chloroform, methyl acetate, diethyl ether, *n*-hexane, potassium chloride, sodium chloride, sulfuric acid, acetic acid and water were of analytical grade and were sourced from VWR International (Darmstadt, Germany). Primuline and acetone were of analytical grade and purchased from Sigma Aldrich. Analytical standard such as triolein (TAG), glyceryl dipalmitate (DAG) and stearic acid (FFA), (PC) phosphatidylcholine, (PE) phosphatidylethanolamine, (Lyso) lysophosphatidylcholine, (PI) phosphatitylinositol, mixture of FAMEs (Fatty Acids Methyl Esters), BSA and glucose were purchased from Sigma Aldrich (Saint-Louis, MO, USA).

### 3.4. Procedure for Construction of the Demixing Curve

The following protocol was followed for the construction of demixing curves: 15 mL glass equipment, a magnetic stirrer plate, thermostat bath, glass buret (30 mL), and a thermometer were used. For example, 0.3192 g ± 0.0001 mg of ethanol (absolute) was mixed with 2.6945 g of water (demineralized) in glass equipment (15 mL) and thermostatted to 20 °C for the construction of the first point of the demixing curve in Figure 1.

Then, with the buret, ethyl acetate was added dropwise to the ethanol–water mixture. When 0.73 mL of ethyl acetate was added, a biphasic system formed as a milky spontaneous emulsion. The mass of ethyl acetate was deduced via a known density. The relative mass composition was established for the given point and indicated in a ternary diagram. The same procedure was realized to construct each point (in triplicate) on the alternative and classical system curve and compared with data published in the literature [19].

### 3.5. Lipid Extractions: Application on Yeast

#### 3.5.1. Bligh and Dyer Extractions: Classical and Alternative Ternary Systems

*Extraction:* The following procedure was used with wet and dry *Yarrowia lipolytica* *IFP29* containing about 14.85% of lipids. To get a controlled moisture content by weight (about 80% of water), water was initially added to the biomass. Then, for all extractions, the three solvents were mixed in such proportions that, taking into account the water in the biomass, monophasic solvent systems were formed as shown in the phase diagrams (Figure 1). Each sample was homogenized for 10 min in different proportions of ethanol–ethyl acetate or methanol–chloroform (ratios for the different points investigated are shown in Figure 1 (blue points). Each monophasic system was notified as 1 to 14 for the chloroform/methanol system and A to L for the ethyl acetate–ethanol system. The final volume of the total mixture was 3 mL.

*Separation:* A sufficient quantity of water containing 0.58% of KCl and apolar solvents (chloroform or ethyl acetate) were added to the solvent monophasic system in order to induce phase separation. Each mixture was homogenized for 10 min. Macroscopically, even three phases appeared with the middle phase containing visible cell debris. For the chloroform–methanol–water system, a clear, mostly chloroform-containing phase formed the lower phase part and a clear, mostly methanol–water mixture (KCl 0.58%) formed the upper phase. Conversely, for the ethyl acetate–ethanol–water system, a clear phase, rich in ethyl acetate, was observed in the upper layer and a clear phase, rich in ethanol–water (KCl 0.58%), was obtained in the lower phase. In both systems, cell debris were present at the interface between the upper and lower phases. The final proportion of mixtures was given in the ternary diagram: points labelled 1′ to 14′ for the chloroform/methanol system and points labelled A′ to L′ for the ethyl acetate/ethanol system. “Organic” and “aqueous” phases were then separated from the middle layer and filtered.

*Analysis:* Lipids, proteins and sugar were measured for all phases. Total lipid content was given by the gravimetric method and confirmed with gas chromatography. The fatty acids profile was obtained by gas chromatography coupled with a flame ionization detector and lipid classes (TAG, DAG, MAG, FFA, PL) by high performance thin-layer chromatography. Proteins and sugars were detected by UV spectrometry (Figure 1).

**Figure 7:**
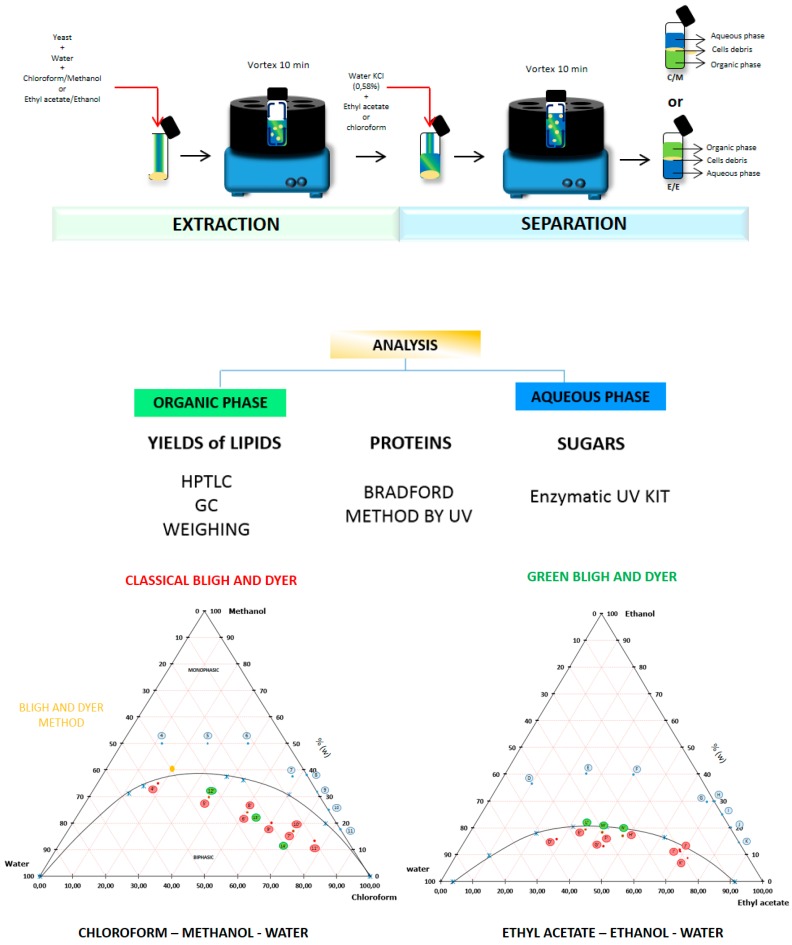
Ternary systems of classical Bligh and Dyer (B &D) (Methanol/Chloroform/Water) and Greener Bligh and Dyer (ethanol–ethyl acetate–water). The black curve separates the monophasic region (**above**) from the diphasic region (**below**).

The blue points give the composition of the monophasic mixtures used for the solid–liquid extraction process (Figure 7). The red and green points denote the compositions used in the second step for the liquid–liquid separation. The blue crosses are the points of the bimodal curve, the orange point in the left diagram denotes the extraction realized with the reference method of Bligh and Dyer (1959).

#### 3.5.2. Classical Bligh and Dyer Procedure

Dried yeast was mixed with distilled water, chloroform and methanol to reach 1:2:0.8 parts chloroform:methanol:water (*v*/*v*/*v*) and homogenized for 10 min. Then, chloroform and water containing 0.85% of KCl were added to get a final ratio of 2:2:1.8 chloroform:methanol:water (*v*/*v*/*v*). The mixture was homogenized for 10 min and finally filtered to remove cell debris. The final biphasic system was then separated into two phases and the lower chloroform phase was collected and analysed to determine the total lipid content, proteins and sugars [7].

### 3.6. Determination of Lipids, Proteins and Sugars

#### 3.6.1. Qualitative and Quantitative Analysis of Total Lipids

##### Evaluation of Total Lipids Content in Tissue by Gravimetry

An aliquot of organic phases containing at least 10 mg of lipids was evaporated to dryness under nitrogen and at 50 °C. The dry residue was weighed and yields were determined with the following equation:

$$\%\;\mathrm{of}\;\mathrm{lipids}=\frac{\mathrm{weight}\;\mathrm{of}\;\mathrm{residue}\times \text{total volume of organic phase}}{\mathrm{weight}\;\mathrm{of}\;\mathrm{yeast}\times \text{evaporated volume}}\times 100$$

##### Analysis by Gas Chromatography Coupled with Flame Ionization Detector

FAMEs (Fatty Acids Methyl Ester) were prepared from the lipid extract using acid-catalyzed transmethylation as described by Li et al. [44]. All extracts were evaporated under nitrogen and at 50 °C. The protocol of transesterification was described in our previous publication: Sicaire et al. [39].

##### Determination by High Performance Thin-Layer Chromatography

Lipids were quantified by a CAMAG 3 TLC scanning densitometer (CAMAG, Muttenz, Switzerland). The protocol of quantification of phospholipids and neutral lipids was explained in our previous work: Breil et al. [11].

#### 3.6.2. Quantitative Analysis of Proteins

Dosage of proteins was realized by Bradford [45] methods. A sample of 100 µL (organic and aqueous phases) is mixed with 1mL of Bradford reactive. After 15 min, the protein concentrations in the sample were determined spectrophotometrically (with UV biochrom, libra S22) at 595 nm. Protein content was calculated with the following equation:

$$\%\;\mathrm{of}\;\mathrm{proteins}=\frac{\mathrm{Concentration}\times (\text{volume of organic phase or volume of aqueous phase})}{\mathrm{weight}\;\mathrm{of}\;\mathrm{yeast}}\times 100$$

BSA was used as standard protein.

#### 3.6.3. Quantitative Analysis of Glucose

Dosage of glucose was carried out via enzymatic analysis (“Biosentech” kit glucose). A sample of 100 µL (organic and aqueous phases) is mixed with 1mL of water and 1.9 mL of buffer (pH: 7.5 with NADP 70 mg and ATP 90 mg). After an UV reading, another buffer solution was added. After 15 min, sugar concentrations in the sample were determined spectrophotometrically (UV biochrom, libra S22) with an absorbance at 340 nm. Sugar yields were calculated with the following equation:

$$\%\;\mathrm{of}\;\mathrm{sugars}=\frac{\mathrm{Concentration}\times (\text{volume of organic phase or volume of aqueous phase})}{\mathrm{weight}\;\mathrm{of}\;\mathrm{yeast}}\times 100$$

## 4. Conclusions

B & D or Folch systems using chloroform/methanol/water were most efficient in terms of selectivity and can be considered as “gold standards” for lipid analysis [17]. Replacement of these solvents by bio-based solvents from agricultural sources, respecting principles of green chemistry [46], is a challenge that we have taken up in the present study. Based on the evaluation of the free energy of solubilization calculated with COSMO-RS and on experimentally determined efficiency and selectivity results, we could show that the greener solvent pair ethyl acetate/ethanol 1.794/0.784 (*w*/*w*) with 0.08 g of wet sample was the most efficient procedure when followed by the addition of water (with 0.8% of KCl) and ethyl acetate 1/2 (*v*/*v*) for the separation of aqueous and organic phases. This new system is selective enough in terms of lipid classes or fatty acids and very efficient in terms of extraction power.

## Figures and Tables

**Table 1:** Conductor-like Screening MOdel for realistic Solvatation (COSMO-RS) results; simulation of modeled molecules in several alternative solvents for the substitution of chloroform and methanol. Green (0), yellow (−0.1 ≤ *x* ≤ −0.99), red (−1 ≤ *x* ≤ −5).

Solvents/Molecules	TAG LLL	TAG LOO	TAG OOO	DAG LGL	DAG LGO	DAG OGO	FFA18 1n9	FFA18 2n6	FFA 16	PC LL	PC OL	PE LL	PE OL	Lanosterol	Ergosterol	1.6 bd Glucan	1.4 bd Glucan	Chitine	Glycerol	Histidine	Arginine	Glucose
Water	−23.96	−24.57	−24.55	−15.09	−17.26	−16.23	−8.18	−7.99	−7.67	−7.86	−5.92	−14.56	−14.13	−10.05	−9.19	0.00	−1.01	−0.21	0.00	0.00	0.00	0.00
Methanol	−3.97	−4.36	−4.39	0.00	−2.73	−1.98	−0.13	−0.26	−0.30	0.00	0.00	−0.11	−0.08	0.00	0.00	0.00	0.00	0.00	0.00	0.00	0.00	0.00
Ethanol	−2.11	−2.41	−2.40	0.00	−0.99	−0.11	−0.10	−0.13	0.12	0.00	0.00	−0.27	−0.16	0.00	0.00	0.00	0.00	0.00	0.00	0.00	0.00	0.00
Isopropanol	0.00	0.00	0.00	−0.19	−0.07	−0.20	0.00	0.00	0.00	0.00	0.00	0.00	0.00	0.00	0.00	0.00	0.00	0.00	0.00	0.00	0.00	0.00
Chloroform	0.00	0.00	0.00	0.00	0.00	0.00	−0.39	−0.17	−0.29	0.00	0.00	0.00	0.00	0.00	−0.22	0.00	−3.65	0.00	0.00	0.00	0.00	−4.66
Ethyl acetate	0.00	0.00	0.00	0.00	0.00	0.00	0.00	0.00	0.00	0.00	0.00	0.00	0.00	0.00	0.00	−7.86	−5.92	−14.56	−14.13	−17.48	−20.22	0.00
MeTHF	0.00	0.00	0.00	0.00	0.00	0.00	0.00	0.00	0.00	0.00	0.00	0.00	0.00	0.00	0.00	0.00	0.00	0.00	0.00	0.00	0.00	0.00
CPME	0.00	0.00	0.00	0.00	0.00	0.00	0.00	0.00	0.00	−0.95	−0.21	0.00	0.00	0.00	0.00	−2.25	0.00	0.00	0.00	0.00	−3.08	0.00
DMC	−0.17	−0.10	−0.10	−0.10	−0.24	−0.19	−0.10	0.00	−0.20	0.00	0.00	−0.65	−0.21	−0.09	−0.06	−0.17	0.00	0.00	0.00	−0.28	−0.20	0.00
Ethyl lactate	−2.71	−3.24	−3.24	0.00	−1.60	−0.09	−0.15	−0.04	−0.14	0.00	0.00	−0.07	−0.04	0.00	0.00	0.00	0.00	0.00	0.00	0.00	0.00	0.00
α−Pinene	0.00	0.00	0.00	0.00	−0.19	−0.17	0.00	0.00	0.00	−3.52	−4.56	0.00	0.00	0.00	0.00	−9.94	0.00	0.00	0.00	0.00	0.00	0.00
*d*−Limonene	0.00	0.00	0.00	−0.20	−0.10	−0.37	0.00	0.00	0.00	−2.93	−3.97	0.00	0.00	−0.07	0.00	−9.29	−8.71	−7.64	−5.11	−4.85	−7.25	−7.55
*p*−Cymene	0.00	0.00	0.00	−0.17	−0.14	−0.14	0.00	0.00	0.00	−2.21	−3.40	0.00	0.00	−0.04	0.00	−8.92	−8.42	−7.31	−4.96	−4.56	−6.96	−7.37

*Triglycerides: TAG LLL* (*R1: C18:2n-6, R2: C18:2n6, R3: C18:2n-6*), *TAG LOO* (*R1: C18:2n-6, R2: C18:1n9, R3: C18:1*), *TAG OOO* (*R1: C18:1n9, R2: C18:1n9, R3: C18:1n9*); *Diglycerides: DAG LGL* (*R1: C18:2n6, R2: C18:2n6*), *DAG LGO* (*R1: C18:2n6, R2: C18:1n9*), *DAG OGO* (*R1: C18:1n9, R2: C18:1n9*); *Free Fatty Acids: FFA 18:1n9, FFA 18 2n6* (*R1: C18:2n6*), *FFA 16* (*R1: C18:2n6*); *Phosphatidylethanolamine: PE LL* (*R1: C18:2n-6, R2: C18:2n6*), *PE LO* (*R1: C18:1n9, R2: C18:2n-6*); *Phosphatidylcholine: PC LL* (*R1: C18:2n-6, R2: C18:2n6*), *PC LO* (*R1: C18:1n9, R2: C18:2n-6*); *Sterols:Lanosterol, Ergosterol. Polysacharides: 1,6 bd, glucan, 1,4 bd, glucan, chitine. Glycerol. Amined acids: Histidine, Arginine. Sugar: Glucose.*

**Table 2:** Crude lipids, distribution of lipid classes, fatty acids, proteins and glucose compositions of extracts (organic and aqueous phases) obtained by extractions with different ratios of classical solvents. Lipid yields and gravimetric yields (g per 100 g of dry matter), lipid classes and fatty acids compositions (relative percentages). Proteins and sugars yields in g per 100 g of dry matter.

Compositions	1	2	3	4	5	6	7	8	9	10	11	12	13	14
**Lipids yieds by GC**	14.55 ± 0.12	11.94 ± 0.52	6.25 ± 0.12	4.32 ± 0.15	5.31 ± 0.23	11.08 ± 0.24	14.12 ± 0.89	13.77 ± 0.56	13.45 ± 0.26	13.62 ± 0.27	13.39 ± 0.85	6.51± 0.07	9.51 ± 0.23	18.50 ± 0.75
**Lipids yields by gravimetry**	14.85 ± 0.10	9.12 ± 0.35	3.5 ± 0.20	4.85 ± 0.12	5.42 ± 0.17	12.25 ± 0.18	14.83 ± 0.42	14.25 ± 0.21	13.89 ± 0.14	14.63 ± 0.17	13.95 ± 0.15	7.02 ± 0.16	10.52 ± 0.24	9.25 ± 0.36
**Lipid classes composition (%)**														
FFA: Free fatty acid	58.69 ± 0.98	57.89 ± 2.75	57.07 ± 0.40	58.96 ± 0.49	58.69 ± 2.13	57.23 ± 1.07	58.93 ± 2.11	58.45 ± 1.04	57.36 ± 1.21	61.23 ± 1.27	58.23 ± 1.40	58.52 ± 1.25	57.85 ± 1.74	58.63 ± 2.36
TAG: Triacylglycerol	20.03 ± 0.52	21.23 ± 1.02	20.96 ± 2.3	22.36 ± 1.02	23.78 ± 1.58	22.45 ± 1.82	21.36 ± 1.02	19.87 ± 0.95	18.65 ± 1.24	16.20 ± 1.52	18.25 ± 1.75	22.52 ± 1.36	20.60 ± 1.95	17.81 ± 1.52
DAG: Diacylglycerol	15.45 ± 0.23	15.26 ± 0.82	15.36 ± 0.47	14.56 ± 1.18	12.58 ± 0.40	14.25 ± 1.11	13.69 ± 1.36	14.25 ± 1.12	14.74 ± 1.23	14.10 ± 1.45	15.24 ± 1.91	13.63 ± 1.14	15.25 ± 1.02	14.63 ± 1.41
PE: Phosphatdylethanolamine	2.58 ± 0.25	2.58 ± 0.80	2.85 ± 0.40	2.47 ± 0.18	4.16 ± 0.42	3.81 ± 0.05	4.76 ± 0.05	3.69 ± 0.12	4.43 ± 0.14	3.69 ± 0.31	3.62 ± 0.25	3.56 ± 0.32	3.58 ± 012	4.52 ± 0.36
PC: Phosphatydylcholine	0.15 ± 0.003	0.23 ± 0.008	0.24 ± 0.003	0.54 ± 0.006	0.24 ± 0.008	0.21 ± 0.007	0.29 ± 0.002	0.17 ± 0.007	0.01 ± 0.001	0.2 ± 0.02	0.14 ± 0.01	0.25 ± 0.01	0.14 ± 0.10	0.96 ± 0.01
PI: Phosphatydylinositol	3.10 ± 0.01	2.81 ± 0.94	3.52 ± 0.05	1.11 ± 0.08	0.55 ± 0.001	2.05 ± 0.008	0.97 ± 0.004	3.57 ± 0.004	4.81 ± 0.20	4.58 ± 0.041	4.52 ± 0.43	1.52 ± 0.04	2.58 ± 0.21	3.45 ± 0.02
**Fatty acids composition (%)**														
C16	10.26 ± 0.02	10.20 ± 0.02	10.49 ± 0.08	11.18 ± 0.07	10.00 ± 0.06	9.89 ± 0.05	10.84 ± 0.04	9.69 ± 0.12	10.62 ± 0.19	11.79 ± 0.03	9.60 ± 0.03	10.03 ± 0.01	9.37 ± 0.06	10.50 ± 0.06
C18	3.25 ± 0.085	3.49 ± 0.005	3.52 ± 0.027	3.76 ± 0.023	3.59 ± 0.018	3.53 ± 0.02	3.91 ± 0.021	3.55 ± 0.05	3.33 ± 0.21	4.11 ± 0.013	3.36 ± 0.014	2.94 ± 0.004	3.55 ± 0.023	3.33 ± 0.019
C16:1n9	4.28 ± 0.005	5.84 ± 0.009	3.98 ± 0.11	5.47 ± 0.009	5.36 ± 0.008	5.42 ± 0.03	5.48 ± 0.096	5.35 ± 0.07	5.57 ± 0.050	4.11 ± 0.041	5.32 ± 0.013	6.20 ± 0.007	5.40 ± 0.036	5.63 ± 0.023
C18:1n9	50.36 ± 0.07	49.81 ± 0.07	50.92 ± 0.39	50.78 ± 0.81	51.39 ± 0.11	51.20 ± 0.30	52.91 ± 0.72	51.26 ± 0.65	51.23 ± 0.10	52.67 ± 0.25	48.37 ± 0.17	49.16 ± 0.062	51.61 ± 0.32	49.79 ± 0.29
C18:2n6	28.39 ± 0.036	29.29 ± 0.04	29.80 ± 0.22	27.44 ± 0.76	28.44 ± 0.07	28.71 ± 0.18	25.76 ± 1.16	28.82 ± 0.35	27.52 ± 0.40	24.85 ± 0.57	28.12 ± 0.093	30.18 ± 0.0038	28.80 ± 0.17	29.38 ± 0.14
C18:3n6	3.46 ± 0.036	1.35 ± 0.002	1.25 ± 0.008	1.34 ± 0.01	1.19 ± 0.007	1.21 ± 0.007	1.07 ± 0.054	1.31 ± 0.027	1.52 ± 0.014	1.00 ± 0.028	5.20 ± 0.029	1.47 ± 0.001	1.24 ± 0.008	1.33 ± 0.013
**Proteins yields (%)**														
Aqueous phases	0.038 ± 0.001	0.328 ± 0.020	0.327 ± 0.030	0.158 ± 0.002	0.175 ± 0.015	0.25 ± 0.023	0.296 ± 0.021	0.314 ± 0.020	0.294 ± 0.025	0.290 ± 0.021	0.284 ± 0.021	0.0195 ± 0.001	0.012 ± 0.001	0.041 ± 0.001
Organic phases	0.0036 ± 1.10 × 10^−4^	0.0058 ± 1.10 × 10^−4^	0.0048 ± 1.10 × 10^−4^	0.0087 ± 1.10 × 10^−4^	0.0084 ± 1.10 × 10^−4^	0.0053 ± 1.10 × 10^−4^	0.0039 ± 1.10 × 10^−4^	0.0021 ± 1.10 × 10^−4^	0.015 ± 1.10 × 10^−4^	0.021 ± 1.10 × 10^−4^	0.0007 ± 1.10 × 10^−4^	0.0002 ± 1.10 × 10^−4^	0.00036 ± 1.10 × 10^−4^	0.00025 ± 1.10 × 10^−4^
**Sugars yields (%)**														
Aqueous phases	0.17 ± 0.010	2.12 ± 0.17	2.23 ± 0.15	2.02 ± 0.18	2.06 ± 0.14	1.97 ± 0.12	1.85 ± 0.17	2.06 ± 0.10	2.04 ± 0.11	2.15 ± 0.14	2.08 ± 0.20	1.33 ± 0.04	0.74 ± 0.01	0.32 ± 0.02
Organic phases	0.13 ± 0.01	0.03 ± 0.001	0.025 ± 0.002	0.023 ± 0.002	0.13 ± 0.001	0.23 ± 0.012	0.12 ± 0.014	0.14 ± 0.017	0.19 ± 0.012	0.02 ± 0.001	0.13 ± 0.02	0.28 ± 0.014	0.08 ± 0.001	0.14 ± 0.001

**Table 3:** Crude lipids, distribution of lipid classes, fatty acids, proteins and glucose compositions of extracts (organic and aqueous phases) obtained by extractions with different ratios of alternative solvents. Lipid yields and gravimetric yields (g per 100 g of dry matter), lipid classes and fatty acids compositions (relative percentages). Proteins and sugars yields in g per 100 g of dry matter.

Compositions	A	B	C	D	E	F	G	H	I	J	K	L	M	N
**Lipids yieds by GC**	13.51 ± 0.09	11.44 ± 0.13	4.52 ± 0.17	6.78 ± 0.52	11.45 ± 0.12	13.53 ± 0.36	14.35 ± 0.63	14.38 ± 12	14.15 ± 0.15	14.24 ± 0.19	14.18 ± 0.13	5.63 ± 0.25	7.58 ± 0.36	8.69 ± 0.50
**Lipids yields by gravimetry**	14.03 ± 0.15	9.53 ± 0.48	5.03 ± 0.38	7.25 ± 0.12	11.97 ± 0.21	14.25 ± 0.46	14.75 ± 0.13	14.65 ± 0.23	14.28 ± 0.60	14.86 ± 0.64	14.52 ± 0.42	6.96 ± 0.31	7.86 ± 0.52	9.19 ± 0.07
**Lipid classes composition (%)**														
FFA: Free fatty acid	59.84 ± 1.52	57.23 ± 2.8	58.62 ± 1.58	58.69 ± 2.53	57.85 ± 1.69	57.37 ± 1.52	58.06 ± 2.12	59.36 ± 1.58	57.38 ± 3.69	57.89 ± 1.88	57.43 ± 1.76	59.56 ± 1.97	59.63 ± 1.45	58.67 ± 1.47
TAG: Triacylglycerol	18.26 ± 0.85	19.20 ± 1.96	20.52 ± 1.02	20.41 ± 1.32	20.33 ± 2.47	19.78 ± 1.75	19.43 ± 1.19	19.85 ± 0.74	21.03 ± 2.12	18.55 ± 1.23	21.81 ± 2.15	18.56+ ± 1.41	16.57 ± 1.41	17.85 ± 1.20
DAG: Diacylglycerol	12.30 ± 1.07	14.85 ± 0.89	15.69 ± 0.47	12.64 ± 1.21	13.67 ± 0.21	14.16 ± 1.03	16.20 ± 1.24	13.60 ± 1.23	12.76 ± 1.12	15.23 ± 1.45	12.87 ± 1.17	13.65 ± 0.55	14.78 ± 0.51	14.52 ± 0.57
PE: Phosphatdylethanolamine	4.58 ± 0.35	3.96 ± 0.32	3.56 ± 0.21	3.30 ± 0.15	3.54 ± 0.12	3.59 ± 0.07	4.01 ± 0.40	3.12 ± 0.21	3.55 ± 0.12	2.63 ± 0.23	4.25 ± 0.12	4.58 ± 0.12	3.65 ± 0.14	4.25 ± 0.12
PC: Phosphatydylcholine	0.33 ± 0.01	0.32 ± 0.01	0.07 ± 0.001	0.05 ± 0.001	0.03 ± 0.001	0.12 ± 0.011	0.45 ± 0.010	0.49 ± 0.041	0.23 ± 2.58	0.14 ± 0.012	0.12 ± 0.013	0.02 ± 0.0018	0.5 ± 0.04	0.08 ± 0.007
PI: Phosphatydylinositol	4.69 ± 0.32	4.44 ± 0.12	1.54 ± 0.05	4.91 ± 0.31	4.58 ± 0.40	4.98 ± 0.48	1.85 ± 0.12	3.58 ± 0.21	5.05 ± 0.54	5.56 ± 0.52	3.52 ± 0.24	3.63 ± 0.21	4.87 ± 0.31	4.63 ± 0.41
**Fatty acids composition (%)**														
C16	9.44 ± 0.48	10.72 ± 0.03	12.20 ± 0.14	7.37 ± 0.77	9.60 ± 0.091	9.42 ± 0.033	10.32 ± 0.005	9.16 ± 0.05	9.70 ± 0.093	8.58 ± 0.092	9.02 ± 0.003	10.80 ± 0.45	9.85 ± 0.065	9.85 ± 0.003
C18	2.92 ± 0.17	3.33 ± 0.01	3.27 ± 0.006	3.43 ± 0.032	3.47 ± 0.028	3.49 ± 0.010	3.96 ± 0.008	3.52 ± 0.023	3.01 ± 0.036	3.52 ± 0.066	3.49 ± 0.001	4.04+ ± 0.13	3.43 ± 0.024	3.43 ± 0.001
C16:1n9	4.77 ± 0.33	5.96 ± 0.020	6.24 ± 0.078	6.16 ± 0.042	5.46 ± 0.047	5.42 ± 0.013	5.43 ± 0.096	5.36 ± 0.035	5.62 ± 0.024	4.67 ± 0.035	5.31 ± 0.002	5.60 ± 0.26	5.26 ± 0.024	5.26 ± 0.002
C18:1n9	47.84 ± 2.01	50.08 ± 0.16	48.45 ± 0.15	61.65 ± 0.43	51.45 ± 0.44	51.64 ± 0.16	53.61 ± 0.62	51.78 ± 0.35	51.02 ± 0.54	56.08 ± 1.99	53.61 ± 0.11	47.89+ ± 0.60	51.36 ± 0.30	51.36 ± 0.119
C18:2n6	32.95 ± 0.59	28.67 ± 0.089	28.53 ± 0.12	30.08 ± 0.19	28.76 ± 0.23	29.78 ± 0.06	25.42 ± 1.06	28.90 ± 0.22	28.66 ± 0.30	26.07 ± 0.23	27.45 ± 0.039	30.38 ± 1.51	28.85 ± 0.16	28.85 ± 0.039
C18:3n6	2.06 ± 0.031	1.21 ± 0.003	1.29 ± 0.007	1.29 ± 0.007	1.23 ± 0.009	1.23 ± 0.002	1.24 ± 0.025	1.25 ± 0.014	1.99 ± 0.074	1.05 ± 0.003	1.09 ± 0.005	1.27 ± 0.069	1.23 ± 0.005	1.23 ± 0.006
**Proteins yields (%)**														
Aqueous phases	0.052 ± 0.001	0.28 ± 0.012	0.326 ± 0.013	0.10 ± 0.009	0.175 ± 0.015	0.205 ± 0.015	0.325 ± 0.012	0.321 ± 0.014	0.324 ± 0.020	0.314 ± 0.012	0.359 ± 0.034	0.211 ± 0.020	0.183 ± 0.012	0.103 ± 0.001
Organic phases	0.0021± 1.10 × 10^−4^	0.0014 ± 10 × 10^−4^	0.0026 ± 2.10 × 10^−4^	0.002 ± 1.10 × 10^−4^	0.015 ± 7.10 × 10^−4^	0.0048 ± 1.10 × 10^−4^	0.0015 ± 1.10 × 10^−4^	0.003 ± 1.10 × 10^−4^	0.0058 ± 1.10 × 10^−4^	0.004 ± 1.10 × 10^−4^	0.0042 ± 1.10 × 10^−4^	0.0036 ± 1.10 × 10^−4^	0.02 ± 1.10 × 10^−4^	0.00025 ± 1.10 × 10^−4^
**Sugars yields (%)**														
Aqueous phases	1.13 ± 0.014	1.10 ± 0.09	2.15 ± 0.15	2.02 ± 0.20	1.95 ± 0.14	2.05 ± 0.12	2.03 ± 0.10	2.12 ± 0.18	1.93 ± 0.21	1.97 ± 0.16	2.04 ± 0.17	0.87 ± 0.074	1.61 ± 0.01	1.69 ± 0.012
Organic phases	0.20 ± 0.001	0.10 ± 0.001	0.20 ± 0.002	0.042 ± 0.001	0.023 ± 0.003	0.021 ± 0.001	0.052 ± 0.001	0.045 ± 0.004	0.036 ± 0.001	0.025 ± 0.001	0.021 ± 0.001	0.085 ± 0.001	0.023 ± 0.002	0.20 ± 0.001

**Table 4:** Results given by COSMO-RS: simulation of modeled molecules in different systems of extractions realized in the experimental part. Green (0), yellow (−0.1 ≤ *x* ≤ −0.99), red (−1 ≤ *x* ≤ −5).

Extractions/Molecules	TAG LLL	TAG LOO	TAG OOO	DAG LGL	DAG LGO	DAG OGO	FFA18 1n9	FFA18 2n6	FFA 16	PC LL	PC OL	PE LL	PE OL	Lanosterol	Ergosterol	1.6 bd Glucan	1.4 bd Glucan	Chitine	Glycerol	Histidine	Arginine	Glucose
D	−9.80	−10.11	−10.10	−5.69	−7.11	−6.31	−2.92	−2.80	−2.70	0.00	0.00	−4.78	−4.64	−4.05	−3.55	0.00	0.00	0.00	0.00	0.00	0.00	0.00
E	−5.99	−6.28	−6.28	−3.14	−4.33	−3.66	−0.14	−0.45	−0.23	0.00	0.00	0.00	−0.28	−2.30	0.00	0.00	0.00	0.00	0.00	0.00	0.00	0.00
F	−3.44	−3.77	−3.78	−0.09	−2.32	−0.23	−0.59	−0.25	−0.28	0.00	0.00	−0.16	−0.18	0.00	0.00	0.00	0.00	0.00	0.00	0.00	0.00	0.00
G	0.00	0.00	0.00	0.00	−0.38	−0.05	0.00	0.00	0.00	0.00	0.00	0.00	0.00	−0.21	−0.27	0.00	0.00	0.00	0.00	0.00	0.00	0.00
H	−0.04	−0.05	−0.05	0.00	−0.07	0.00	0.00	0.00	0.00	0.00	0.00	0.00	0.00	−0.04	0.00	0.00	0.00	0.00	0.00	0.00	0.00	0.00
I	0.00	0.00	−0.09	−0.08	0.00	0.00	−0.03	0.00	0.00	0.00	0.00	0.00	0.00	0.00	0.00	0.00	0.00	0.00	0.00	0.00	0.00	0.00
J	0.00	−0.25	−0.14	0.00	0.00	0.00	0.00	0.00	0.00	0.00	0.00	0.00	0.00	0.00	0.00	0.00	0.00	0.00	0.00	0.00	0.00	0.00
K	0.00	−0.09	−0.06	0.00	−0.07	0.00	0.00	0.00	0.00	0.00	0.00	0.00	0.00	−0.04	0.00	0.00	0.00	0.00	0.00	0.00	0.00	0.00
L	−6.97	−6.97	−6.63	−3.59	−4.83	−4.15	−0.43	−0.19	−0.27	0.00	0.00	−2.51	−2.34	−2.65	−2.13	0.00	0.00	0.00	0.00	0.00	0.00	0.00
M	−4.88	−4.87	−4.51	−0.12	−3.26	−2.58	−0.46	−0.28	−0.39	0.00	0.00	−0.18	−0.15	0.00	0.00	0.00	0.00	0.00		−0.08	0.00	0.00
N	−0.03	0.00	−0.02	−0.40	−0.03	−0.21	−0.01	0.00	−0.01	0.00	0.00	0.00	0.00	−0.04	0.00	0.00	0.00	0.00	0.00	0.00	0.00	0.00
Water	−23.96	−24.57	−24.55	−15.09	−17.26	−16.23	−8.18	−7.99	−7.67	−7.86	−5.92	−14.56	−14.13	−10.05	−9.19	0.00	−1.01	−0.21	0.00	0.00	0.00	0.00
Ethanol	−2.41	−2.40	−2.26	0.00	−0.03	−0.08	0.00	0.00	0.00	0.00	−0.27	−0.16	0.00	0.00	0.00	0.00	0.00	0.00	0.00	0.00	0.00	0.00
Ethyl acetate	0.00	0.00	0.00	0.00	0.00	0.00	0.00	0.00	0.00	0.00	0.00	0.00	0.00	0.00	0.00	−7.86	−5.92	−14.56	−14.13	−17.48	−20.22	0.00
Water	−23.96	−24.57	−24.55	−15.09	−17.26	−16.23	−8.18	−7.99	−7.67	−7.86	−5.92	−14.56	−14.13	−10.05	−9.19	0.00	−1.01	−0.21	0.00	0.00	0.00	0.00

*Triglycerides: TAG LLL* (*R1: C18:2n-6, R2: C18:2n6, R3: C18:2n-6*), *TAG LOO* (*R1: C18:2n-6, R2: C18:1n9, R3: C18:1*), *TAG OOO* (*R1: C18:1n9, R2: C18:1n9, R3: C18:1n9*); *Diglycerides: DAG LGL* (*R1: C18:2n6, R2: C18:2n6*), *DAG LGO* (*R1: C18:2n6, R2: C18:1n9*), *DAG OGO* (*R1: C18:1n9, R2: C18:1n9*); *Free Fatty Acids: FFA 18:1n9, FFA 18 2n6* (*R1: C18:2n6*), *FFA 16* (*R1: C18:2n6*); *Phosphatidylethanolamine: PE LL* (*R1: C18:2n-6, R2: C18:2n6*), *PE LO* (*R1: C18:1n9, R2: C18:2n-6*); *Phosphatidylcholine: PC LL* (*R1: C18:2n-6, R2: C18:2n6*), *PC LO* (*R1: C18:1n9, R2: C18:2n-6*); *Sterols:Lanosterol, Ergosterol. Polysacharides: 1,6 bd, glucan, 1,4 bd, glucan, chitine. Glycerol. Amined acids: Histidine, Arginine. Sugar: Glucose.*

## Supplementary Materials

Supplementary materials can be found at www.mdpi.com/1422-0067/18/4/708/s1.

## Abbreviations

COSMO-RS	Conductor-like screening model for realistic solvation
HSP	Hansen solubility parameters
RED	Relative energy difference
HPTLC	High performance thin-layer chromatography
GC	Gas chromatography
FID	Flame ionization detector
CPME	Cyclopentyl methyl ether
MeTHF	2-methyltetrahydrofuran
DMC	Dimethyl carbonate
IPA	Isopropanol
EtOAc	Ethyl acetate
TAGs	Triglycerides
DAGs	Diglycerides
MAGs	Monoglycerides
FFAs	Free fatty acids
PLs	Phospholipids
FAMEs	Fatty acid methyl esters
