# Supplementary material for: “Bligh and Dyer” and Folch Methods for Solid–Liquid–Liquid Extraction of Lipids from Microorganisms. Comprehension of Solvatation Mechanisms and towards Substitution with Alternative Solvents
Source: Int J Mol Sci. 2017 Mar 27;18(4):708. doi: 10.3390/ijms18040708 (PMC5412294; doi:10.3390/ijms18040708)
Supplement: Supplementary file 1 [file ijms-18-00708-s001.pdf]

Bligh & Dyer” and Folch methods for solid-liquid-liquid extraction of lipids from microorganisms. Comprehension of solvatation mechanisms and towards substitution with alternative solvents.

Cassandra Breil [a], Maryline Vian [a], Thomas Zemb [b], Werner Kunz [c], Farid Chemat [a]

a- Université d’Avignon et des Pays du Vaucluse, INRA, UMR 408, F-84000 Avignon, France
b- Institut de chimie séparative de Marcoule, 30207, Bagnols Sur Ceze
c- Université de Regenburg, 96053 Regenburg, Allemagne

Graphical abstract :

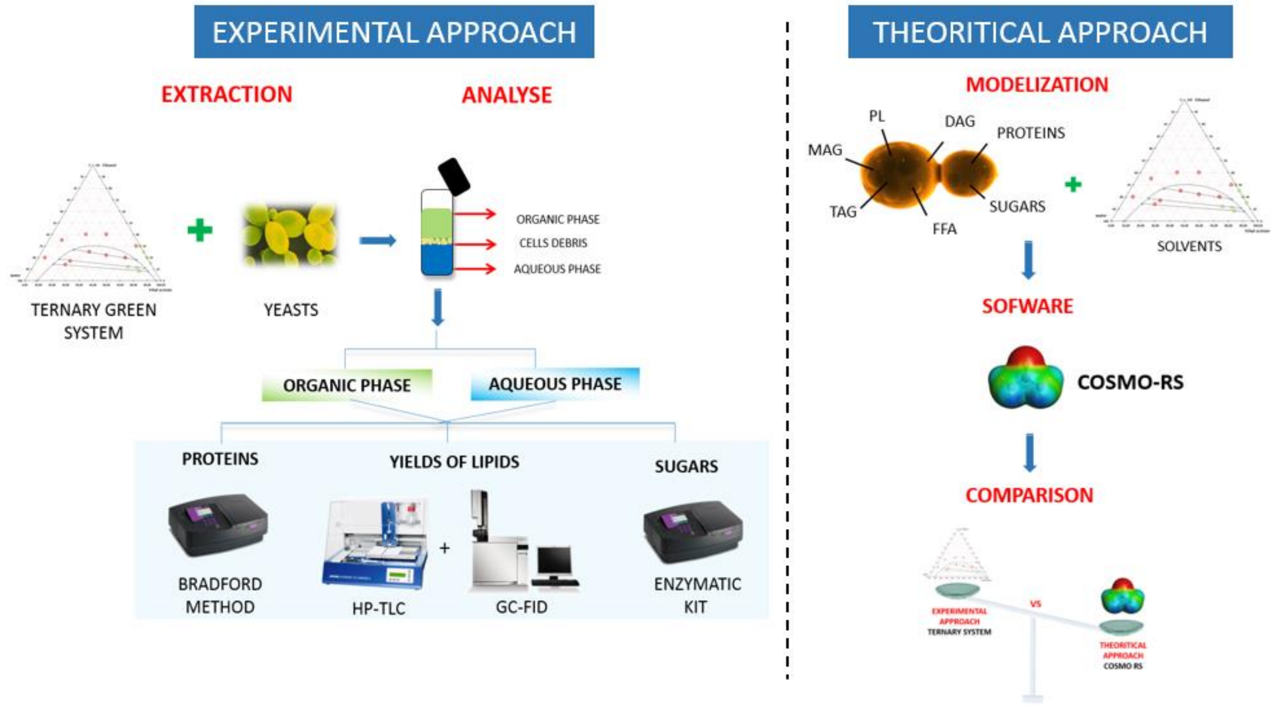

BLIGH AND DYER FROM 1959 TO 2016

Cells brain

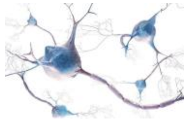

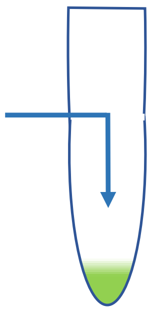

Add MeOH/CHCl3

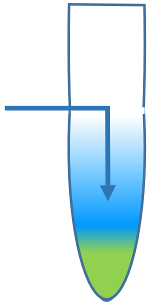

MONOPHASIC

EXTRACTION

Agitation

Salts

$CHCl_3$

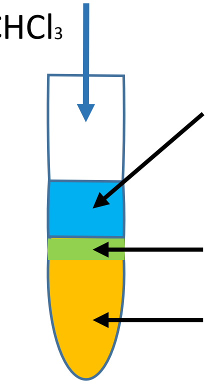

Aqueous phase

Debris

Lipidic phase

BIPHASIC

SEPARATION

Evaporation

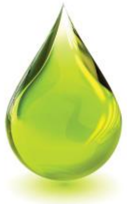

OIL

YIELDS BY GRAVIMETRY

Cells brain

Microalgae

Yeasts

Plants/ Vegetable

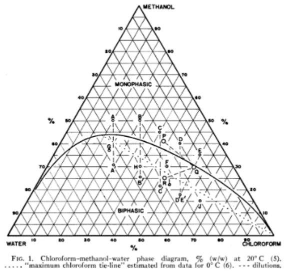

OPTIMIZATION

Bligh and Dyer et al., 1959

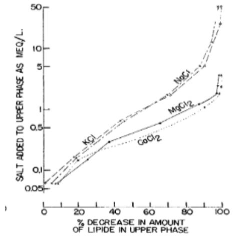

ANALYSE OF COMPOUNDS IN PHASES

Folch et al., 1957

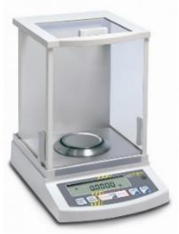

Folch : (2:1 v:v) CHCl3:MeOH

Bligh and Dyer (1:2 v:v) CHCl3:MeOH

Salt : KCl or NaCl (0,5 to 0,9%)

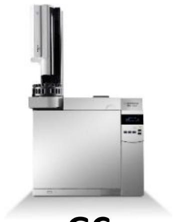

GC

BLIGH AND DYER FROM 1959 TO 2016

MODIFIED EXTRACTIONS

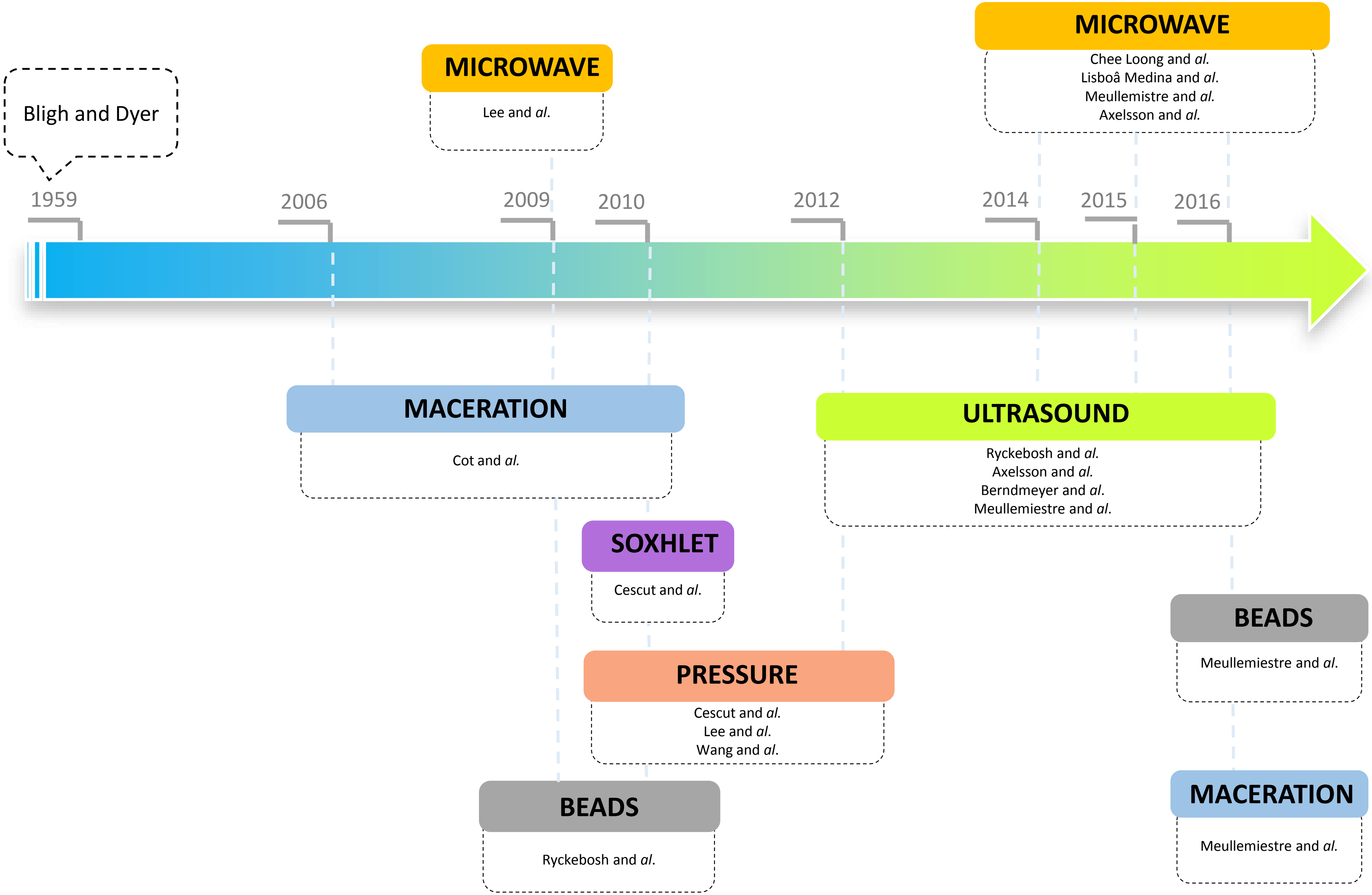

BLIGH AND DYER FROM 1959 TO 2016

ALTERNATIVE SOLVENTS

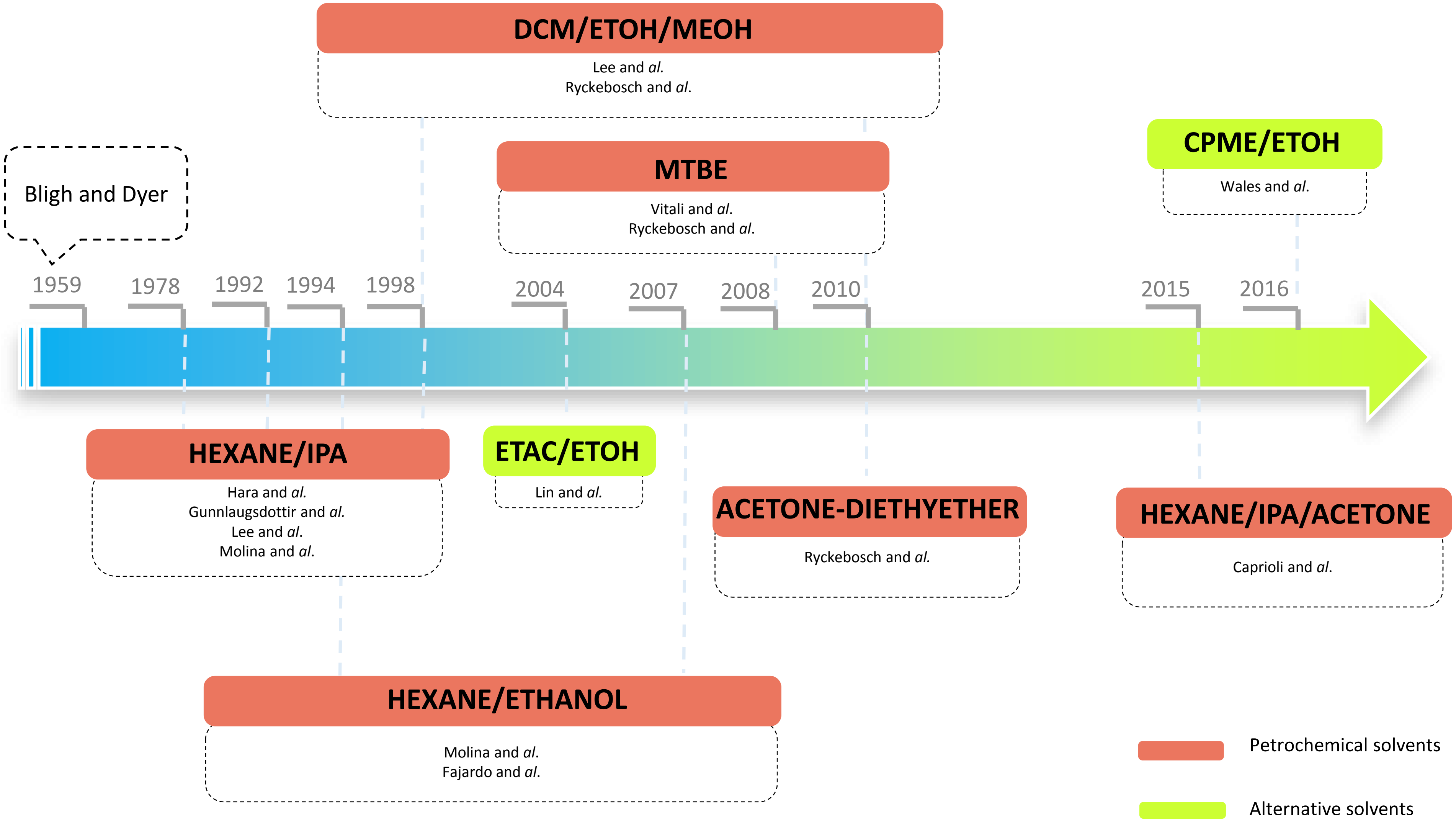

MODELIZATION PART
UTILISATION OF COSMO-RS

BIBLIOGRAPHY **PREVIOULY STUDY**

+ +

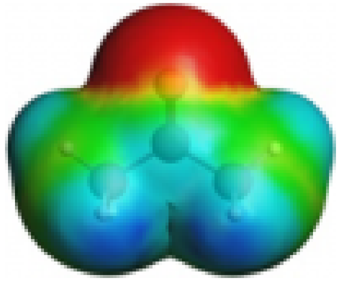

MODELISATION (COSMO-RS)

=

TO FOUND THE BEST SOLVENTS FOR THE SUBSTITUTION OF CHLOROFORM AND METHANOL

MOLECULES MODEL FOR MODELISATION BY COSMO-RS

NEUTRAL LIPIDS

O = Oleic
L = Linoleic
G = Glycerol

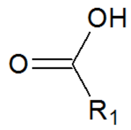

Free Fatty Acids
FFA

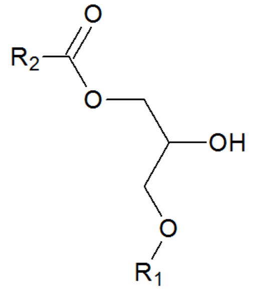

Diglycerides
DAG

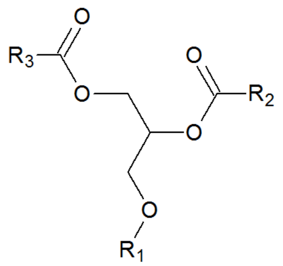

Triglycerides
TAG

POLAR LIPIDS

O = Oléic
L = Linoleic

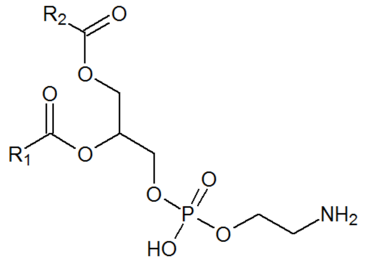

Phosphatidylethanolamine
PE

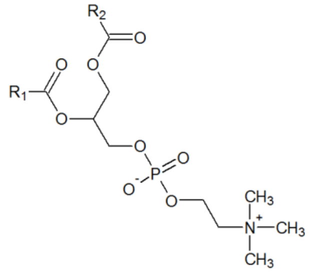

Phosphatidylcholine
PC

OTHER LIPIDS

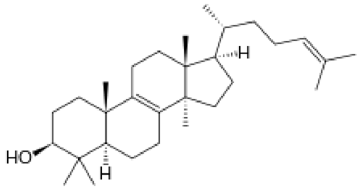

Lanosterol

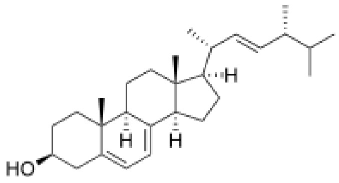

Ergosterol

MOLECULES MODEL FOR MODELISATION BY COSMO-RS

POLYSACCHARIDES

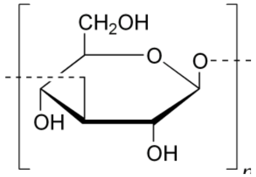

1,3 bd glucan

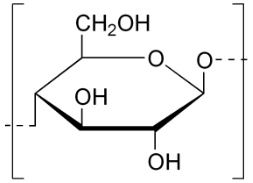

1,4 bd glucan

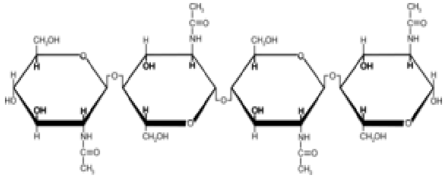

Chitin

AMINED ACIDS

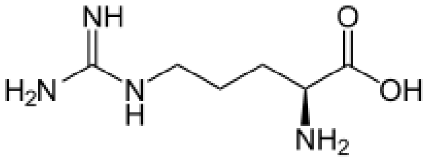

Arginine

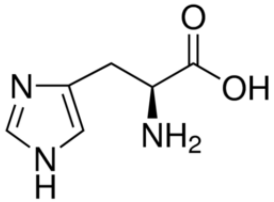

Histidine

SUGARS

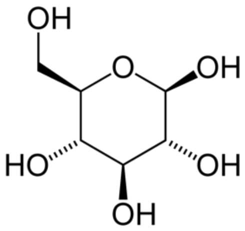

Glucose

COSMO-RS : CHOICE OF SOLVENTS

SUBSTITUTION OF POLAR SOLVENT

	TAG LLL	TAG LOO	TAG OOO	DAG LGL	DAG LGO	DAG OGO	FFA18 1n9	FFA18 2n6	FFA 16	PC LL	PC OL	PE LL	PE OL	Lanosterol	Ergosterol	1,6bd glucan	1,4bd glucan	Chitine	Glycerol	Histidine	Arginine	Glucose
Water	-23,9561	-24,5679	-24,5454	-15,0881	-17,2616	-16,2308	-8,1786	-7,9878	-7,6721	-7,8643	-5,9187	-14,5558	-14,1337	-10,0524	-9,1910	0,0000	-1,0140	-0,2142	0,0000	0,0000	0,0000	0,0000
Methanol	-3,9699	-4,3604	-4,3908	0,0000	-2,7268	-1,9762	-0,1310	-0,2645	-0,3015	0,0000	0,0000	-0,1122	-0,0839	0,0000	0,0000	0,0000	0,0000	0,0000	0,0000	0,0000	0,0000	0,0000
Ethanol	-2,1145	-2,4067	-2,3964	0,0000	-0,9853	-0,1139	-0,1002	-0,1253	0,1236	0,0000	0,0000	-0,2683	-0,1585	0,0000	0,0000	0,0000	0,0000	0,0000	0,0000	0,0000	0,0000	0,0000
Isopropanol	0,0000	0,0000	0,0000	-0,1854	-0,0699	-0,1992	0,0000	0,0000	0,0000	0,0000	0,0000	0,0000	0,0000	0,0000	0,0000	0,0000	0,0000	0,0000	0,0000	0,0000	0,0000	0,0000

Ethanol is as effective as methanol

SUBSTITUTION OF APOLAR SOLVENT

	TAG LLL	TAG LOO	TAG OOO	DAG LGL	DAG LGO	DAG OGO	FFA18 1n9	FFA18 2n6	FFA 16	PC LL	PC OL	PE LL	PE OL	Lanosterol	Ergosterol	1,6bd glucan	1,4bd glucan	Chitine	Glycerol	Histidine	Arginine	Glucose
Chloroforme	0,0000	0,0000	0,0000	0,0000	0,0000	0,0000	-0,3938	-0,1731	-0,2931	0,0000	0,0000	0,0000	0,0000	0,0000	-0,2150	0,0000	-3,6513	0,0000	0,0000	0,0000	0,0000	-4,6582
Ethyl acetate	0,0000	0,0000	0,0000	0,0000	0,0000	0,0000	0,0000	0,0000	0,0000	0,0000	0,0000	0,0000	0,0000	0,0000	0,0000	-7,8643	-5,9187	-14,5558	-14,1337	-17,4794	-20,2240	0,0000
MeTHF	0,0000	0,0000	0,0000	0,0000	0,0000	0,0000	0,0000	0,0000	0,0000	0,0000	0,0000	0,0000	0,0000	0,0000	0,0000	0,0000	0,0000	0,0000	0,0000	0,0000	0,0000	0,0000
CPME	0,0000	0,0000	0,0000	0,0000	0,0000	0,0000	0,0000	0,0000	0,0000	-0,9473	-0,2089	0,0000	0,0000	0,0000	0,0000	-2,2471	0,0000	0,0000	0,0000	0,0000	-3,0847	0,0000
DMC	-0,1729	-0,1005	-0,1009	-0,1019	-0,2355	-0,1909	-0,0995	0,0000	-0,1979	0,0000	0,0000	-0,6542	-0,2090	-0,0943	-0,0619	-0,1709	0,0000	0,0000	0,0000	-0,2789	-0,1958	0,0000
Ethyl lactate	-2,7127	-3,2395	-3,2374	0,0000	-1,5955	-0,0852	-0,1523	-0,0361	-0,1368	0,0000	0,0000	-0,0679	-0,0368	0,0000	0,0000	0,0000	0,0000	0,0000	0,0000	0,0000	0,0000	0,0000
a-Pinene	0,0000	0,0000	0,0000	0,0000	-0,1939	-0,1747	0,0000	0,0000	0,0000	-3,5168	-4,5568	0,0000	0,0000	0,0000	0,0000	-9,9356	0,0000	0,0000	0,0000	0,0000	0,0000	0,0000
d-Limonene	0,0000	0,0000	0,0000	-0,1974	-0,0979	-0,3663	0,0000	0,0000	0,0000	-2,9340	-3,9700	0,0000	0,0000	-0,0732	0,0000	-9,2923	-8,7092	-7,6394	-5,1142	-4,8487	-7,2534	-7,5514
p-Cymene	0,0000	0,0000	0,0000	-0,1749	-0,1422	-0,1426	0,0000	0,0000	0,0000	-2,2102	-3,3977	0,0000	0,0000	-0,0381	0,0000	-8,9211	-8,4173	-7,3125	-4,9631	-4,5576	-6,9593	-7,3712

$\Rightarrow$ Research of solvent which don't solubilize polysaccharides, amino acids and glucose

Ethyl acetate

OBJECTIVE OF THE STUDY

CLASSICAL BLIGH & DYER

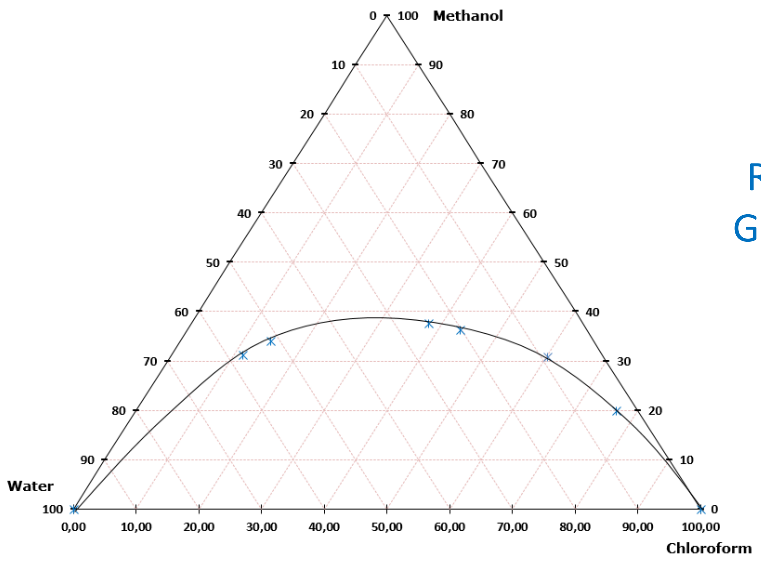

WATER - METHANOL - CHLOROFORM

REPLACE BY A GREEN SYSTEM

GREEN BLIGH & DYER

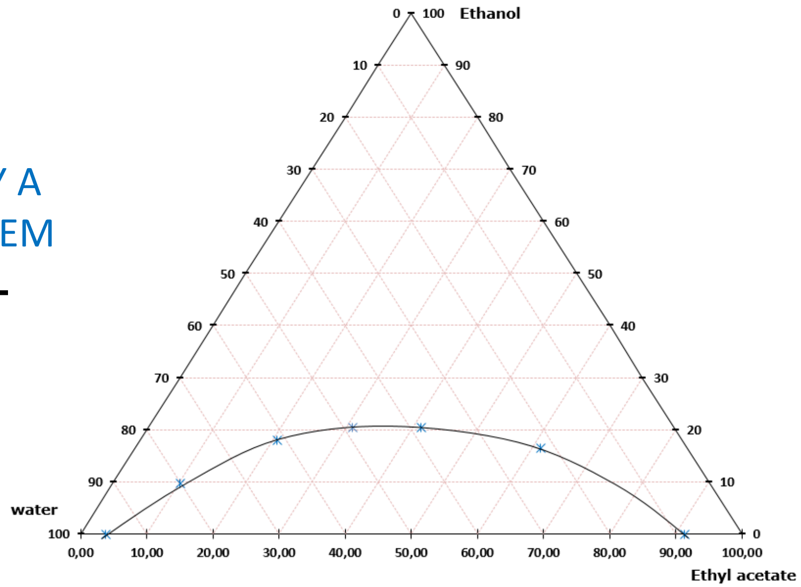

WATER - ETHANOL - ETHYL ACETATE

- To determine the role of each solvents for the extraction of:
  - Lipids
  - Proteins
  - Sugars

- Influence of yields in classical system and alternative system in various conditions :
  - Lipids
  - Proteins
  - Sugars

BIOMASS USED

Biomass : Yeast (*Yarrowia lipolytica IFP29*)

Caracteristics:

- 14,5% of lipids
- 0,33% of proteins
- 2,25% of sugars
- 10% of DM

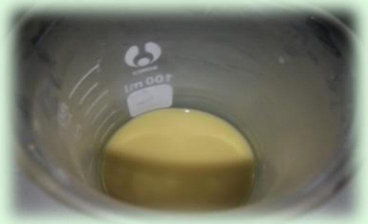

WET

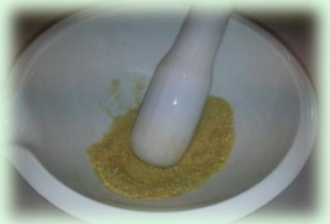

DRY

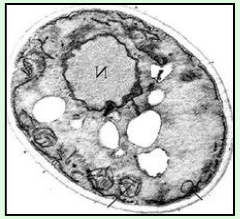

MET

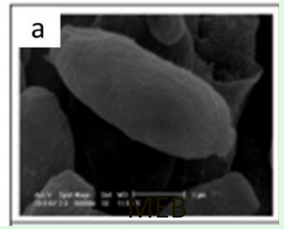

MEB

RELATIVE FATTY ACIDS PROFIL BY GC:

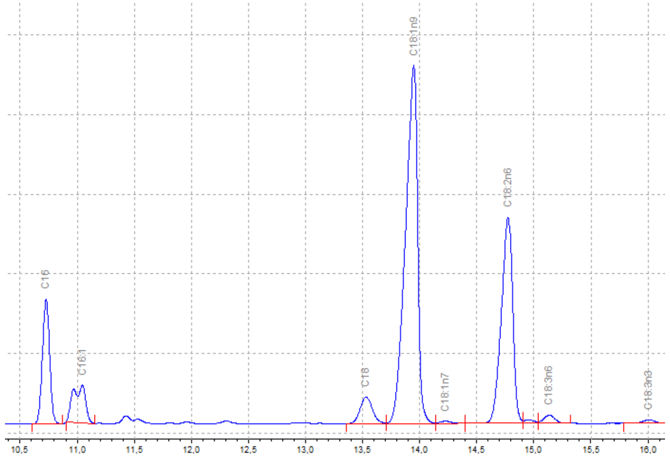

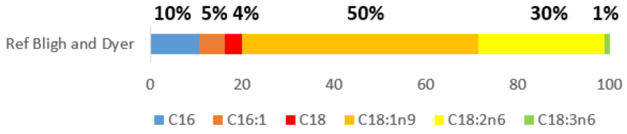

RELATIVE LIPID CLASSES PROFIL BY HPTLC:

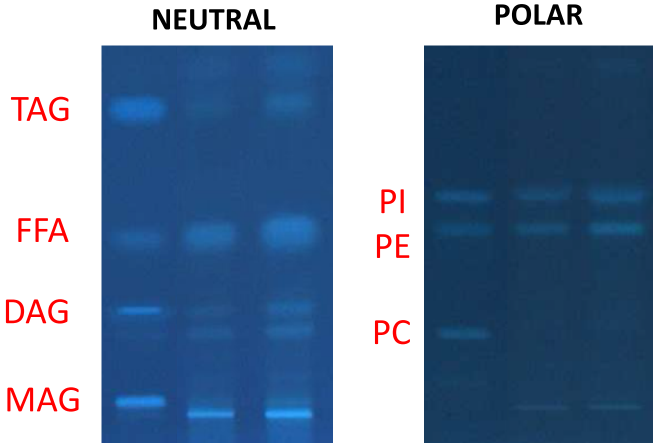

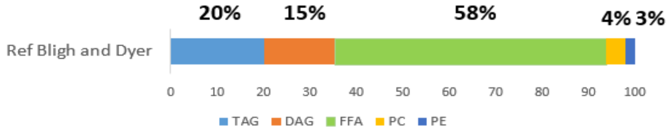

ROLE OF EACH SOLVENT

Comprehension of the role of each solvent for the extraction of sugar and lipid proteins

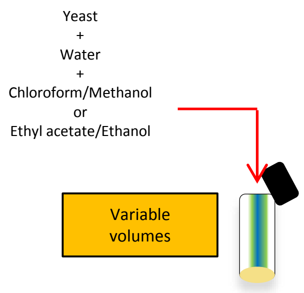

Extraction with pure solvents (solid-liquid extraction step)

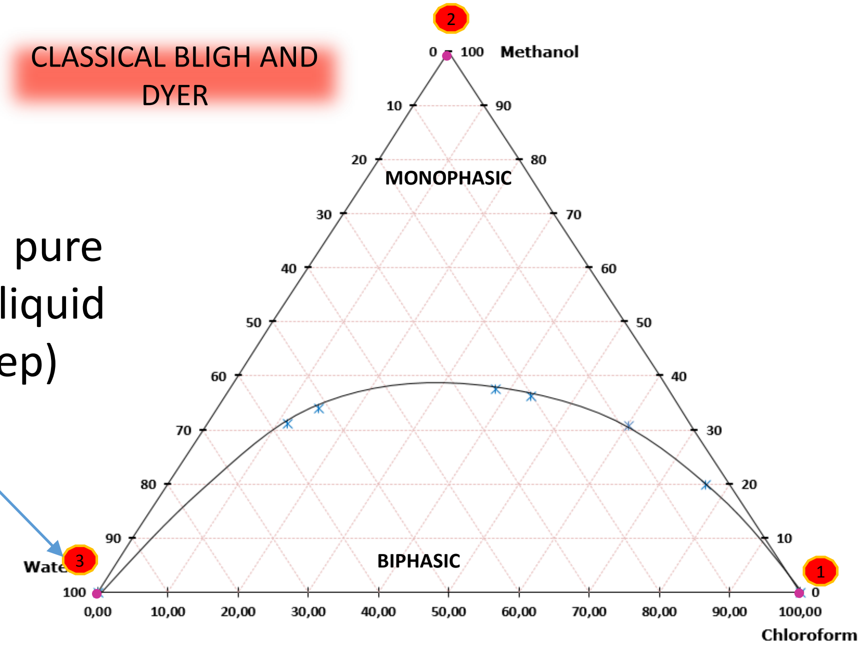

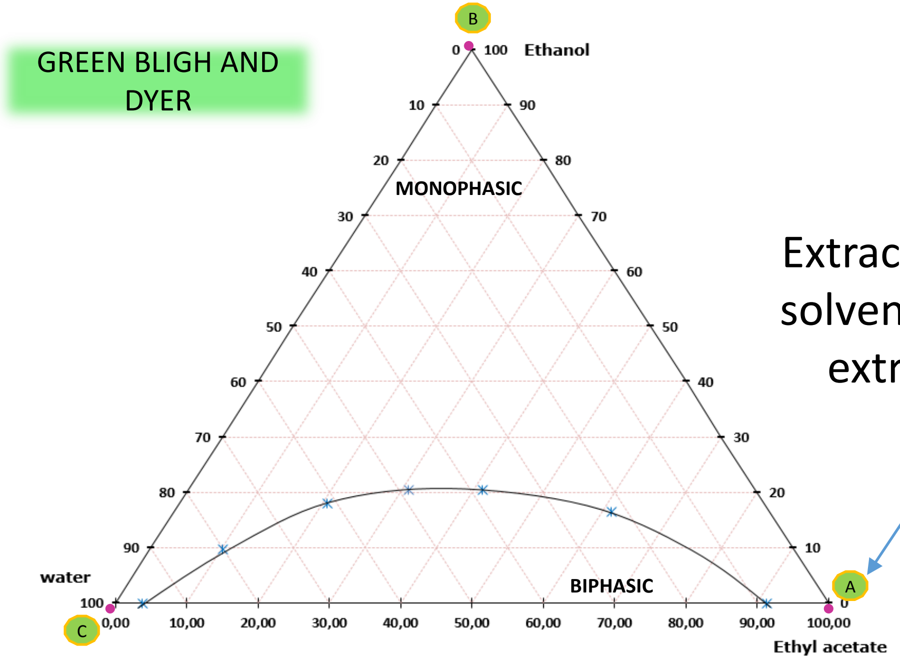

Extraction with pure solvents (solid-liquid extraction step)

ROLE OF EACH SOLVENT

Comprehension of the role of each solvent for the extraction of sugar and lipid proteins

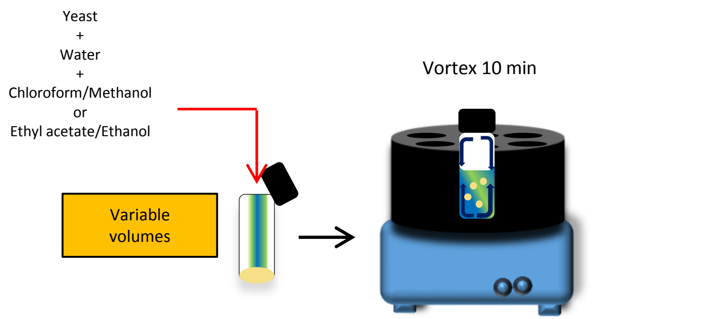

EXTRACTION

CLASSICAL BLIGH AND DYER

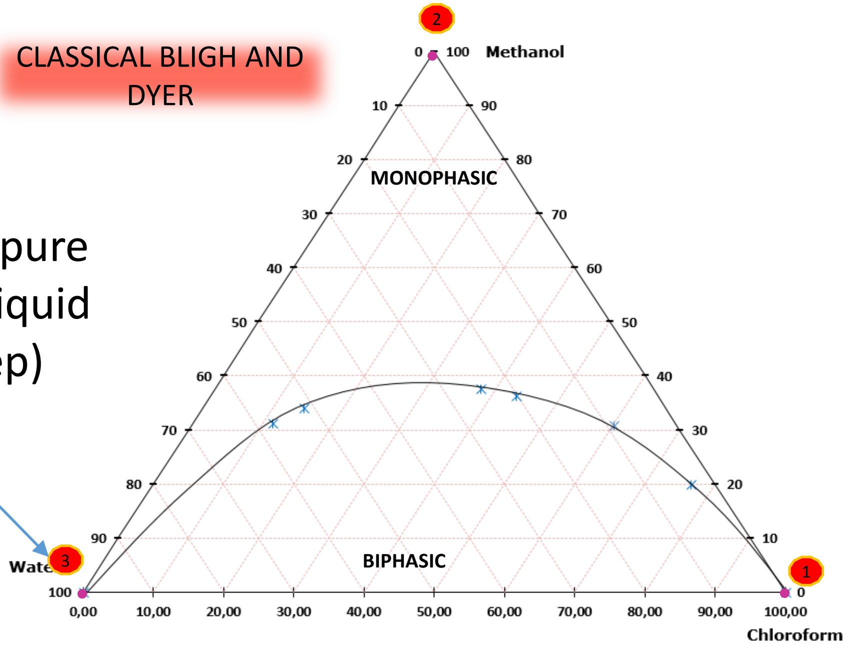

GREEN BLIGH AND DYER

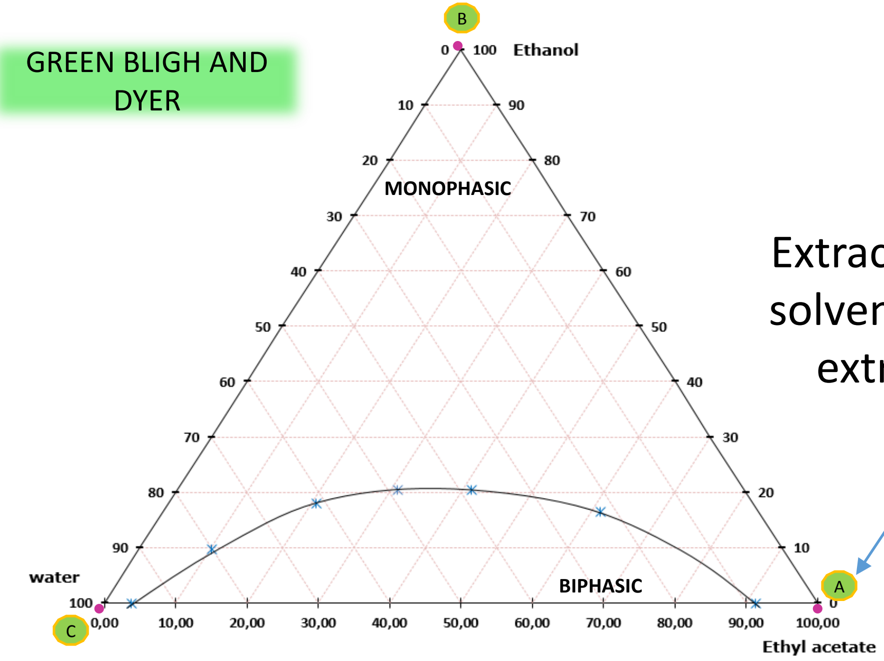

Extraction with pure solvents (solid-liquid extraction step)

Extraction with pure solvents (solid-liquid extraction step)

ROLE OF EACH SOLVENT

Comprehension of the role of each solvent for the extraction of sugar and lipid proteins

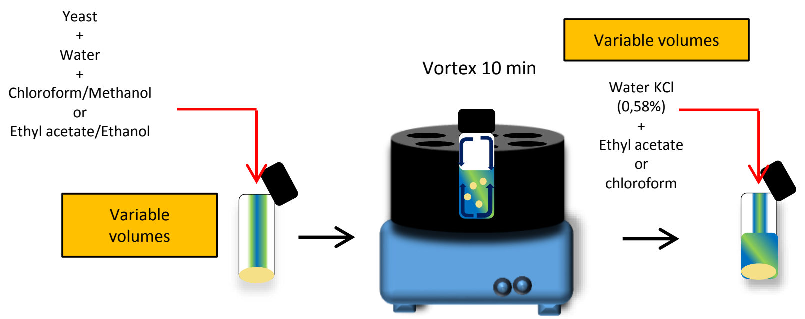

EXTRACTION

CLASSICAL BLIGH AND DYER

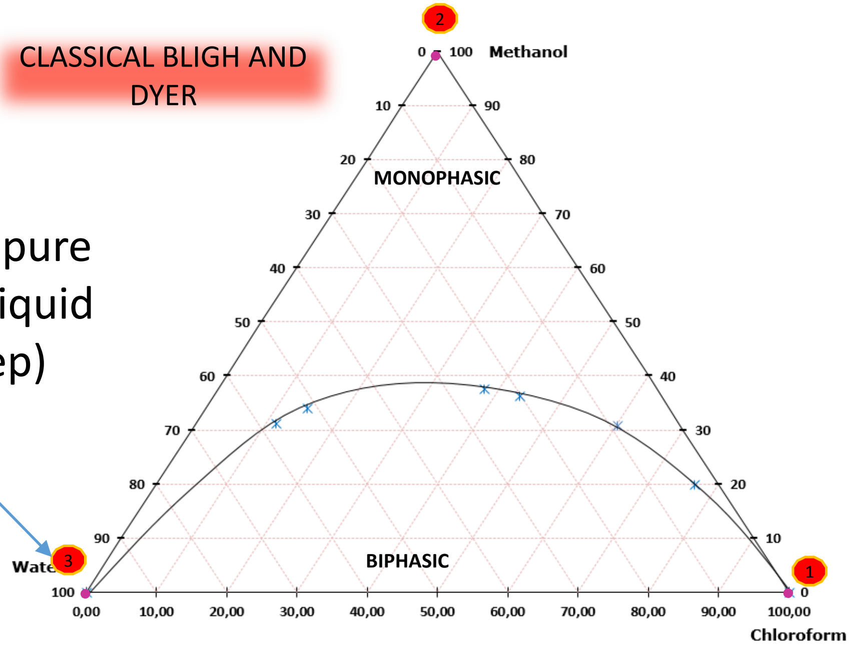

Extraction with pure solvents (solid-liquid extraction step)

GREEN BLIGH AND DYER

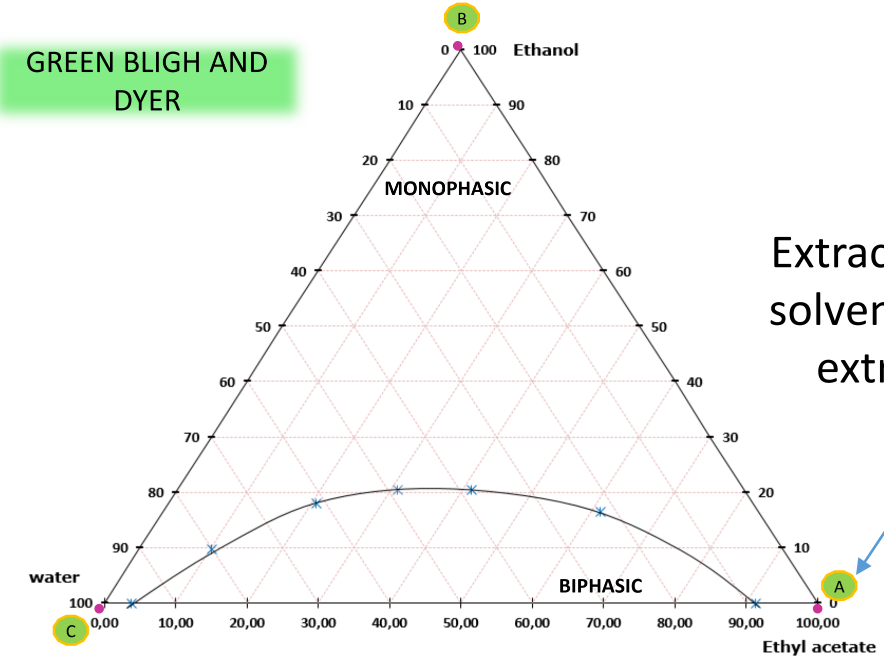

Extraction with pure solvents (solid-liquid extraction step)

ROLE OF EACH SOLVENT

Comprehension of the role of each solvent for the extraction of sugar and lipid proteins

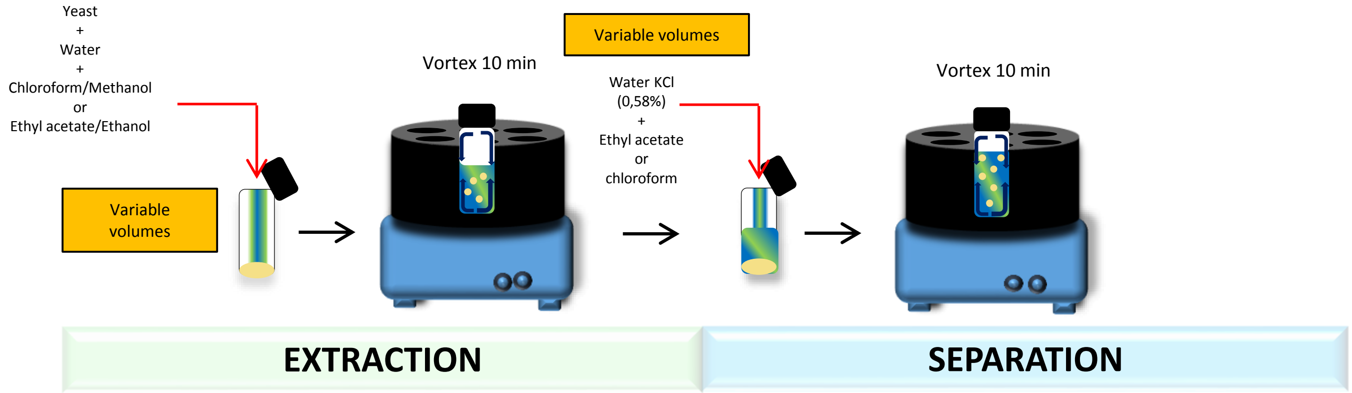

CLASSICAL BLIGH AND DYER

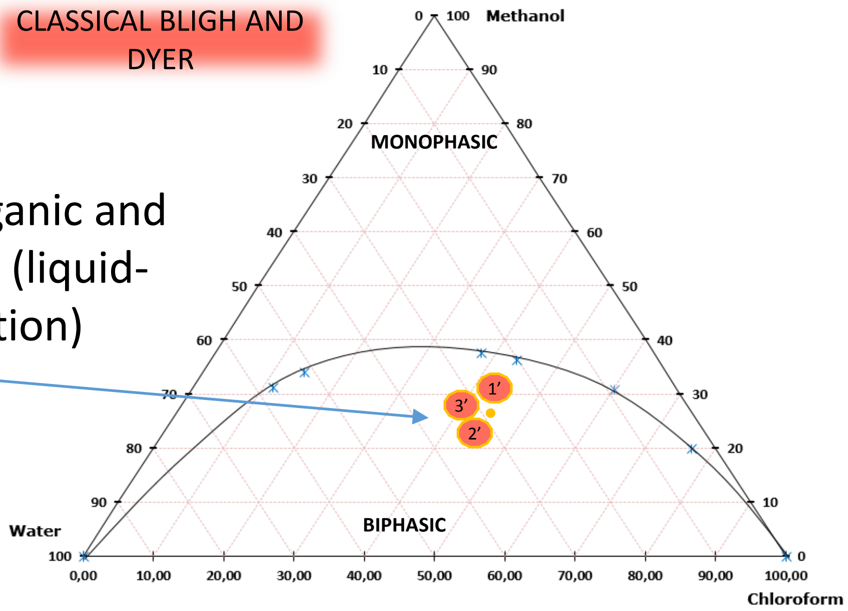

Separation of organic and aqueous phases (liquid-liquid extraction)

GREEN BLIGH AND DYER

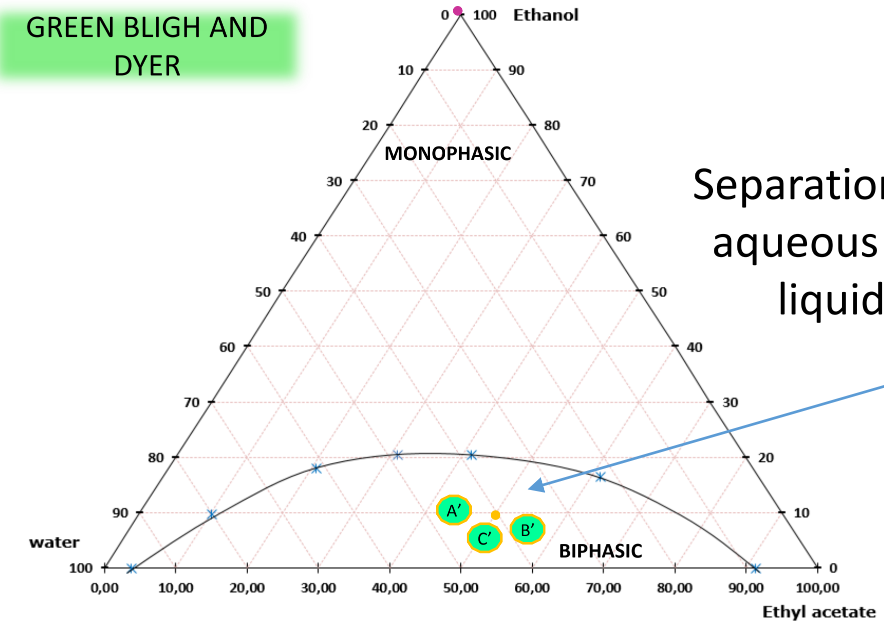

Separation of organic and aqueous phases (liquid-liquid extraction)

ROLE OF EACH SOLVENT

Comprehension of the role of each solvent for the extraction of sugar and lipid proteins

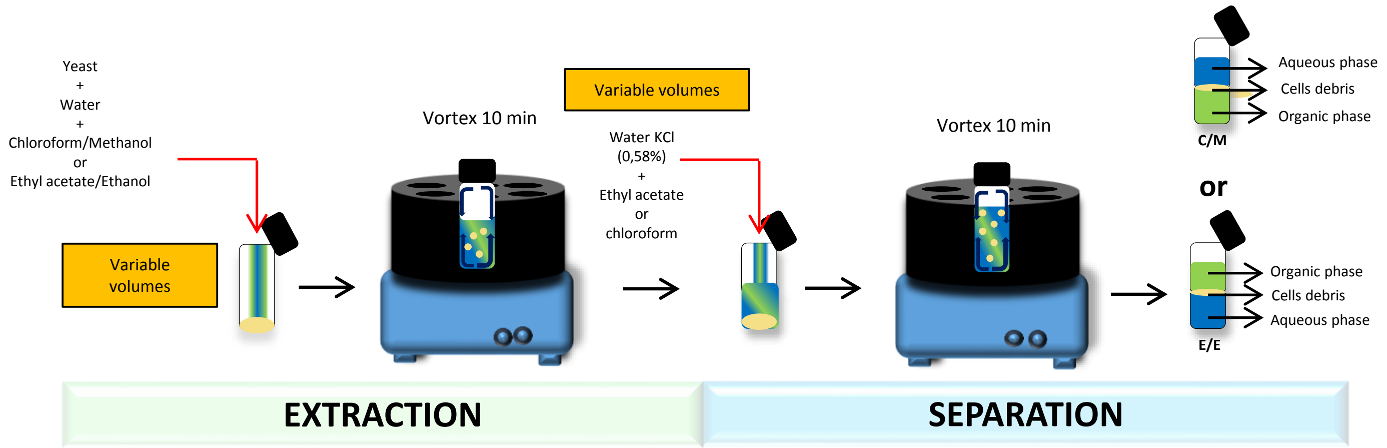

CLASSICAL BLIGH AND DYER

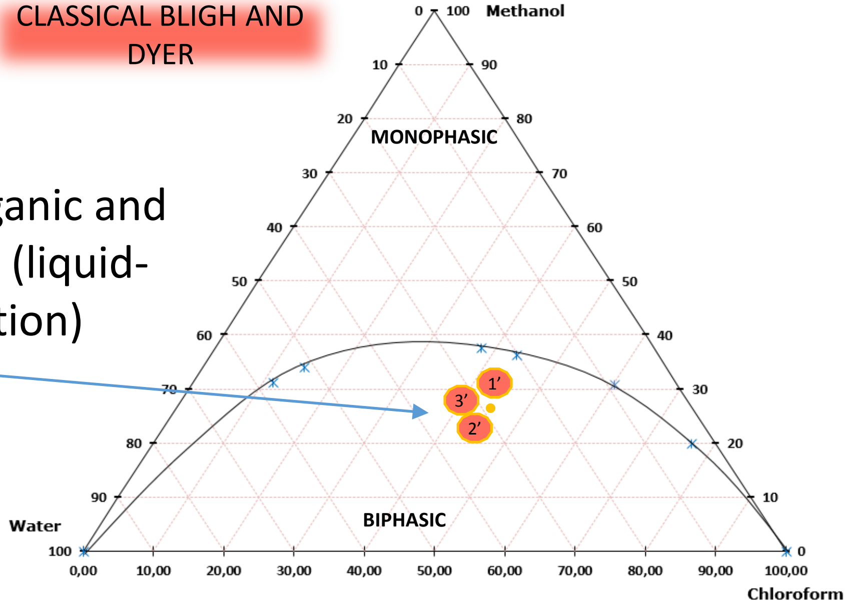

Separation of organic and aqueous phases (liquid-liquid extraction)

GREEN BLIGH AND DYER

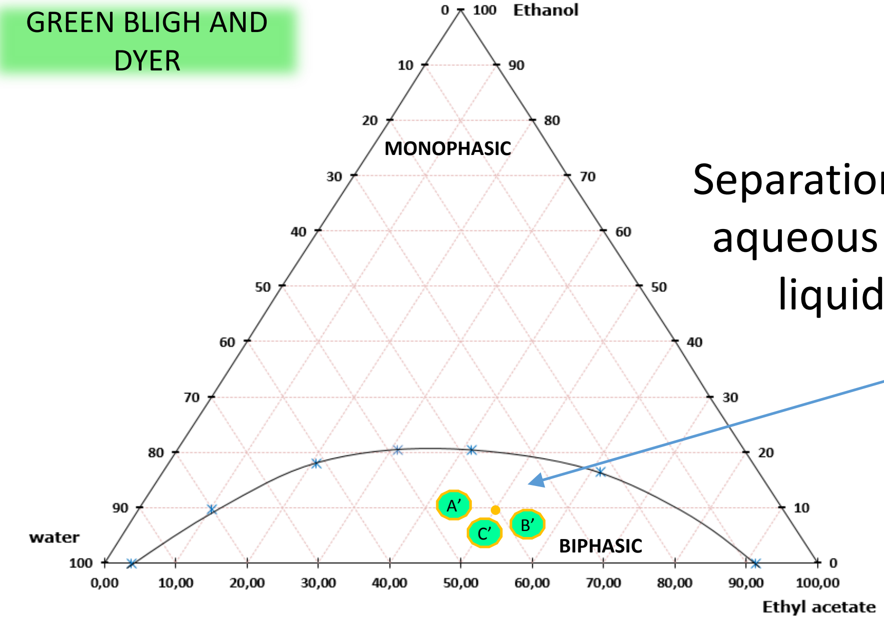

Separation of organic and aqueous phases (liquid-liquid extraction)

ANALYSIS

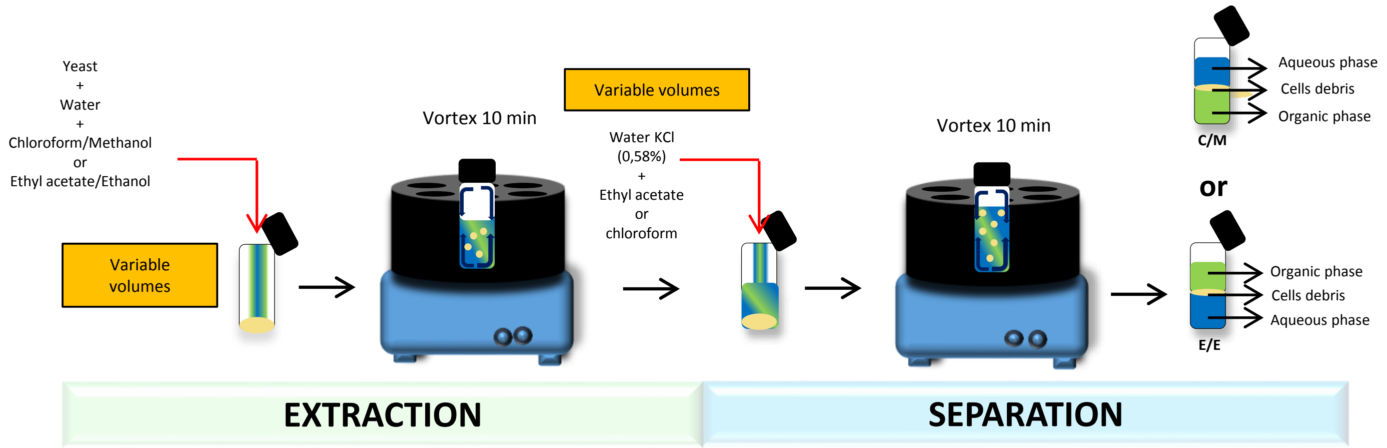

Analyzes of the different compounds in aqueous and organic phases

ANALYSIS

ORGANIC PHASE

AQUEOUS PHASE

YIELDS of LIPIDS

HPTLC
GC
WEIGHING

PROTEINS

BRADFORD METHOD BY UV

SUGARS

Enzymatic UV KIT

ROLE OF EACH SOLVENT

GREEN BLIGH & DYER

ETHYL ACETATE

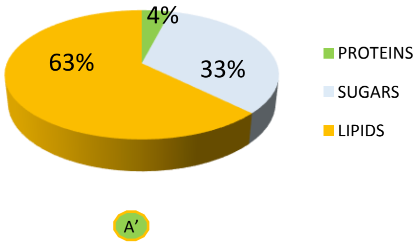

ETHANOL

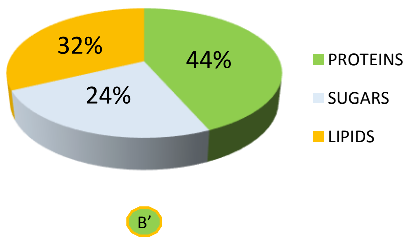

WATER

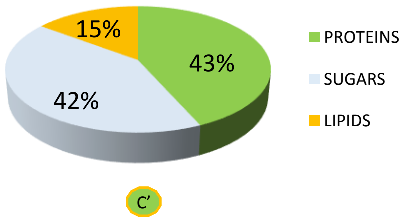

CLASSICAL BLIGH & DYER

CHLOROFORME

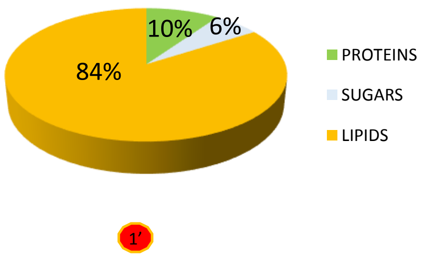

METHANOL

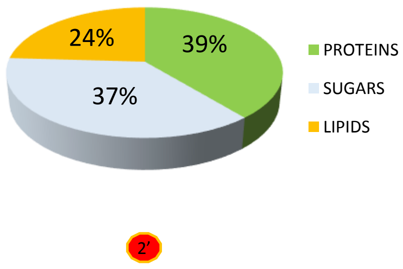

WATER

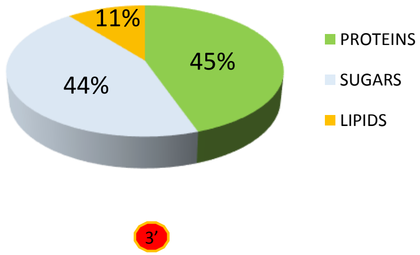

ETHYL ACETATE / CHLOROFORM

- EXTRACT LIPIDS

ETHANOL / METHANOL / WATER

- EXTRACT GLUCOSE AND PROTEINS
- EXTRACT FEW LIPIDS

EXTRACTION PROCEDURE

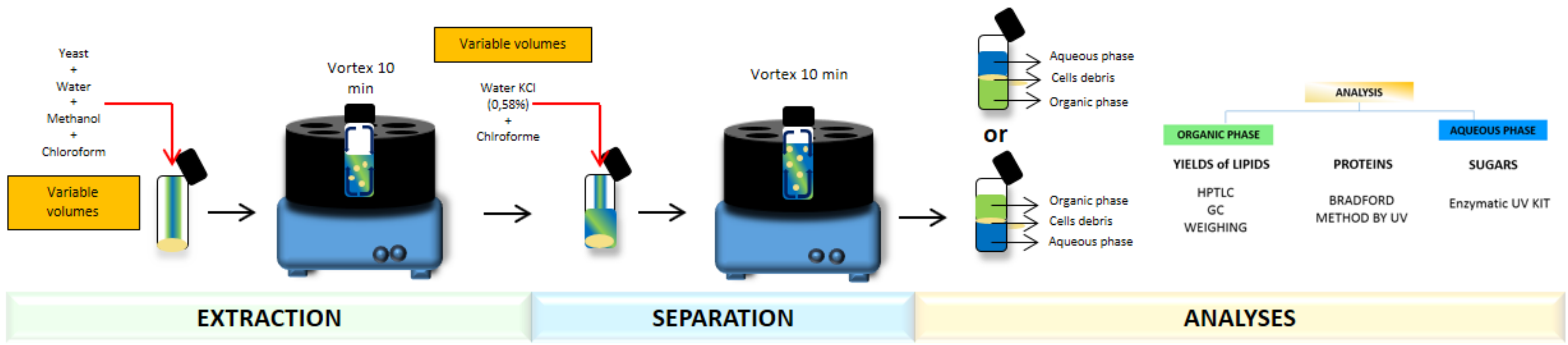

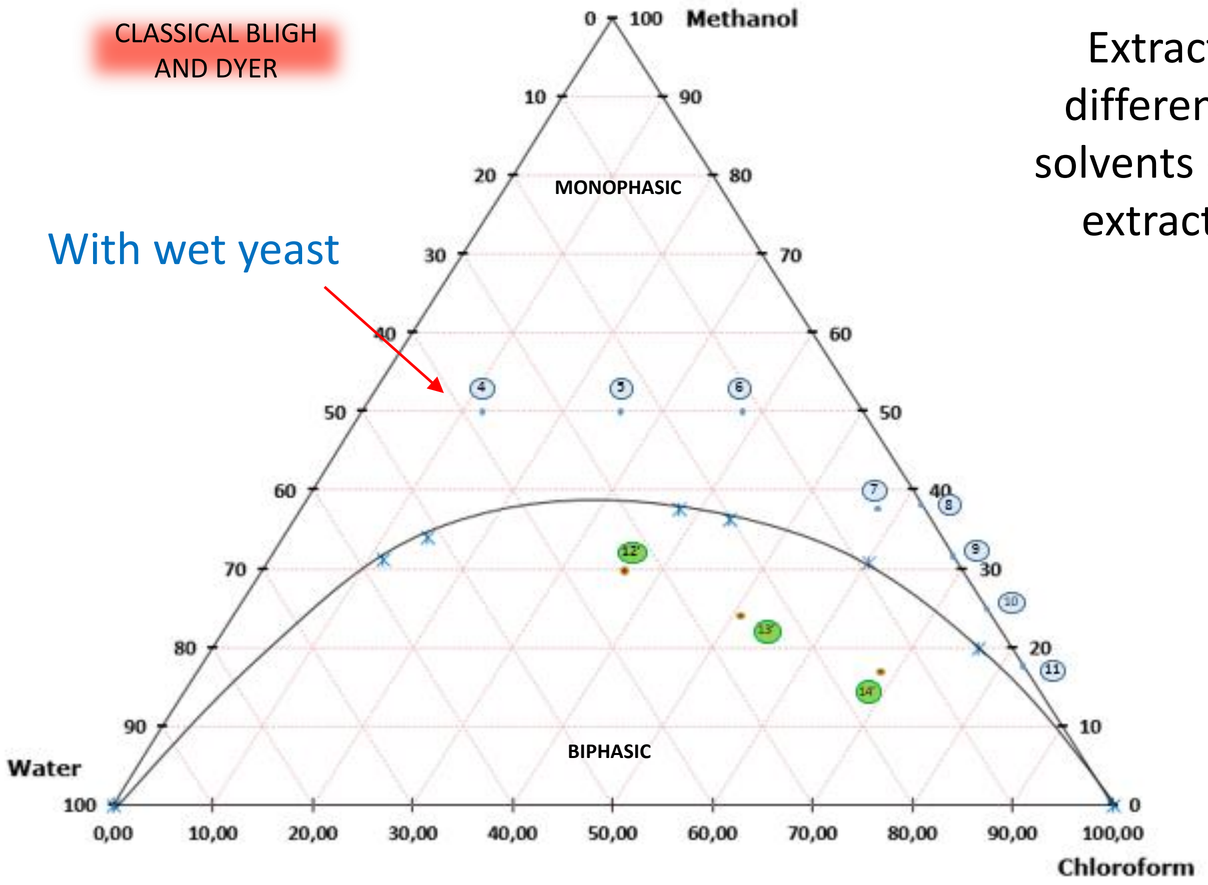

Extraction with different ratios of solvents (solid-liquid extraction step)

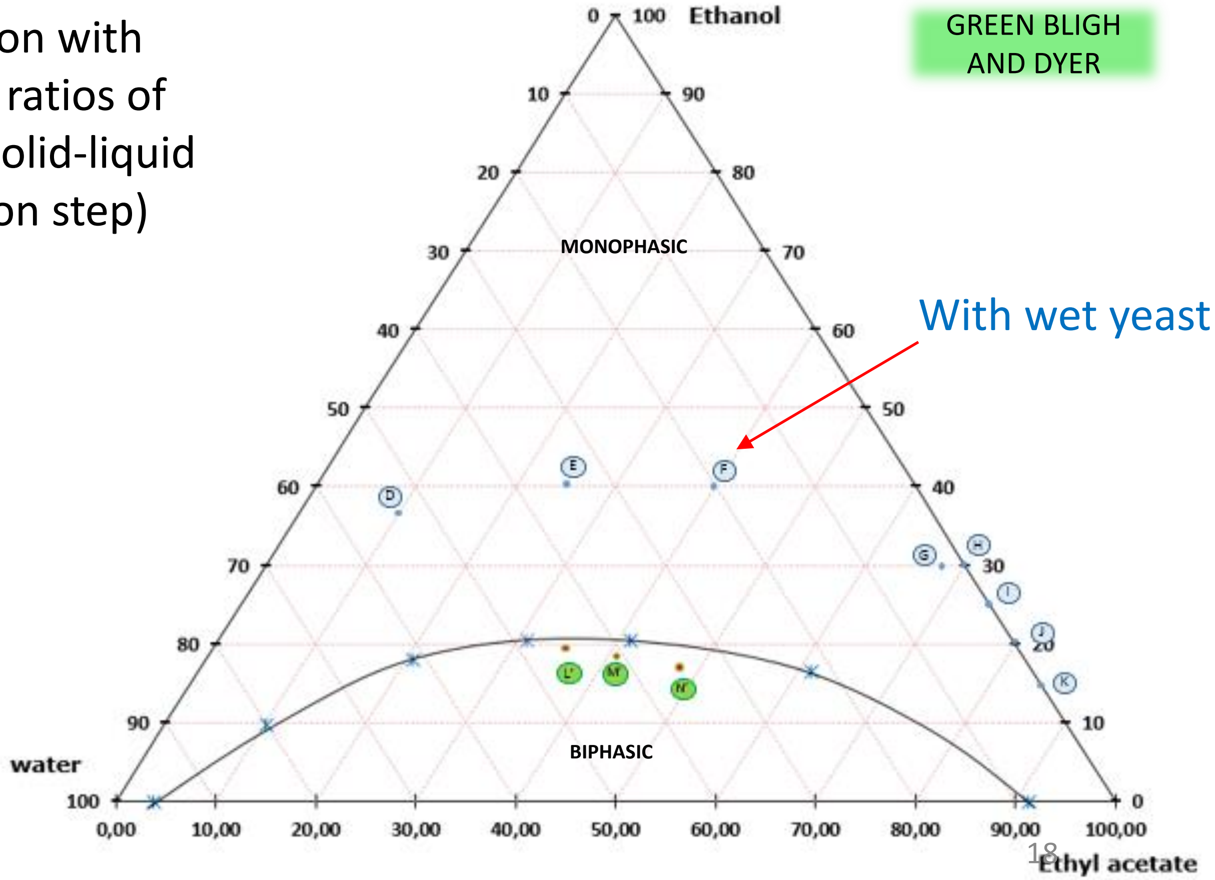

EXTRACTION PROCEDURE

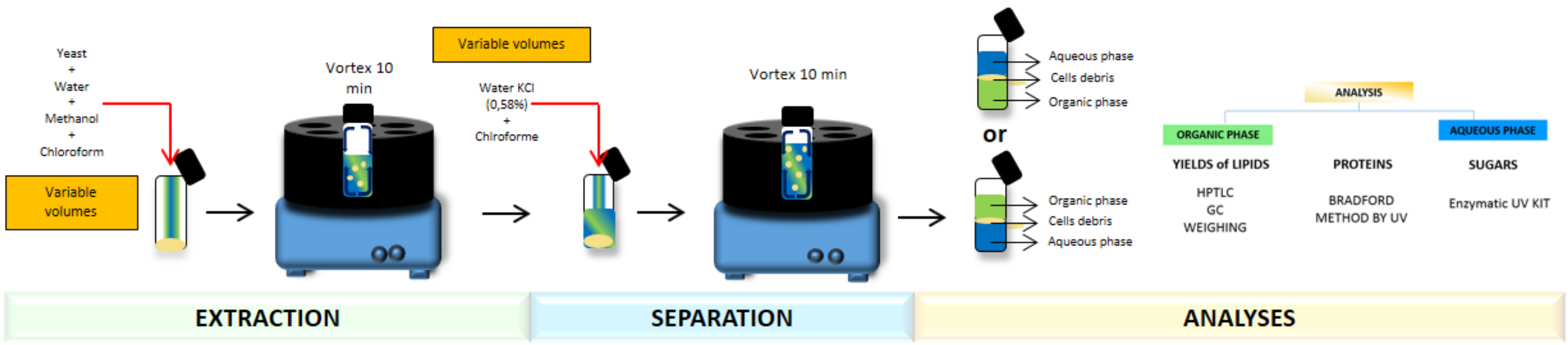

CLASSICAL BLIGH AND DYER

With wet yeast

With dry yeast

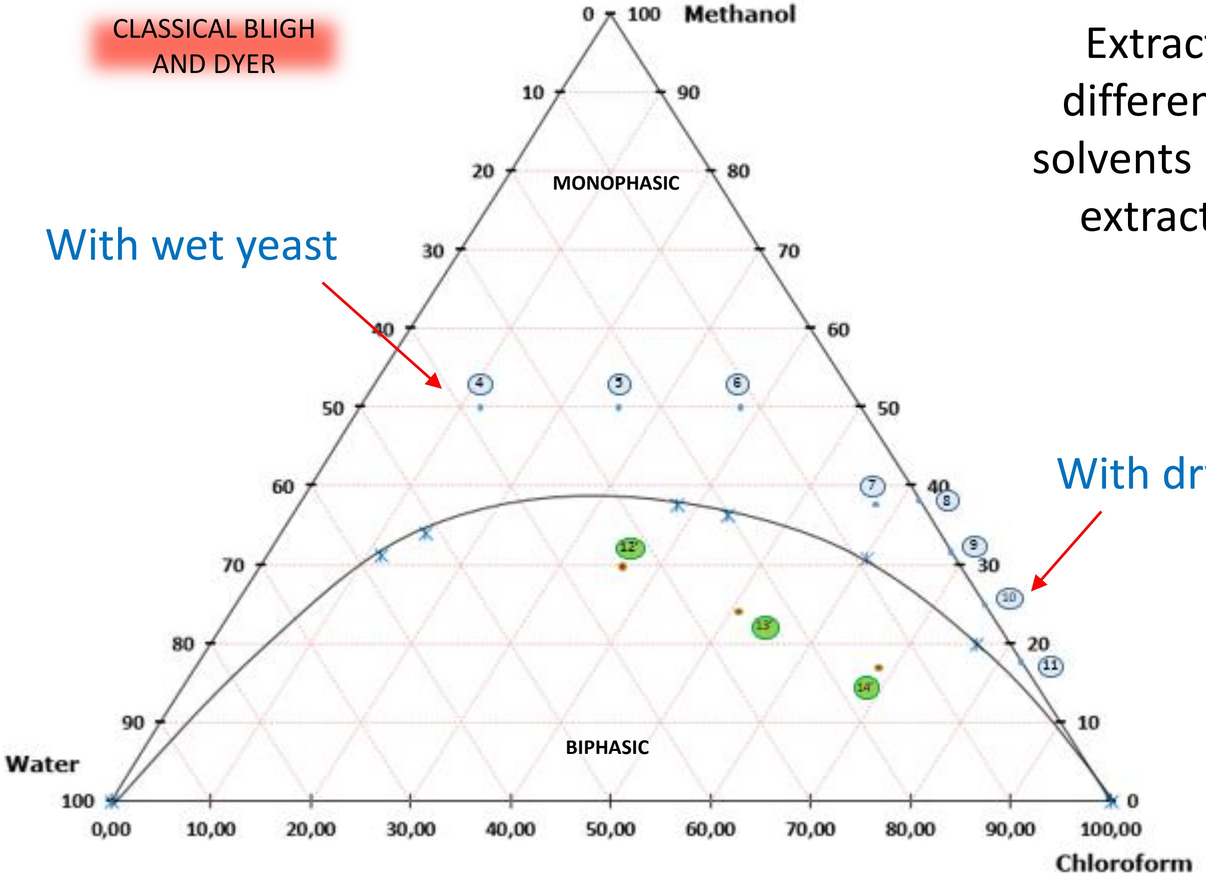

Extraction with different ratios of solvents (solid-liquid extraction step)

GREEN BLIGH AND DYER

With wet yeast

With dry yeast

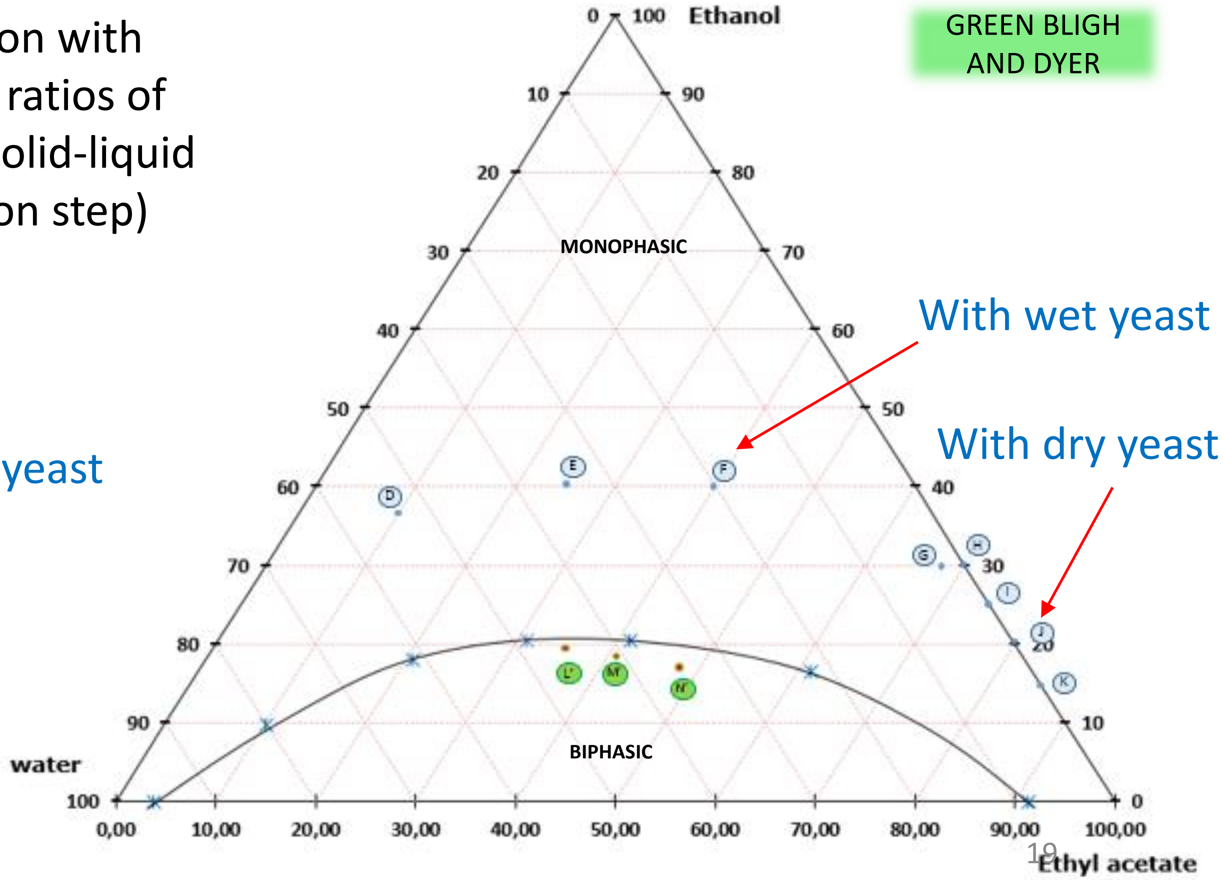

EXTRACTION PROCEDURE

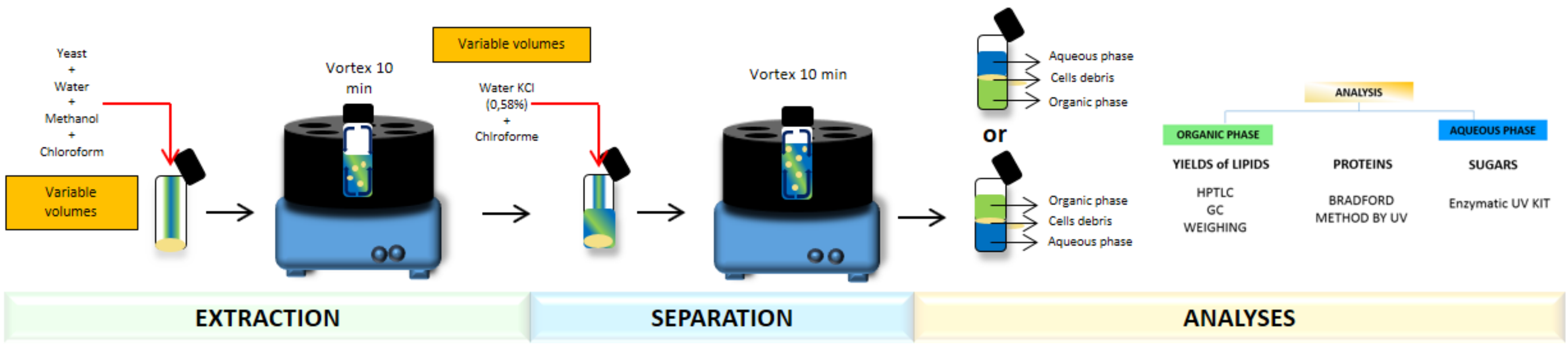

CLASSICAL BLIGH AND DYER

With wet yeast

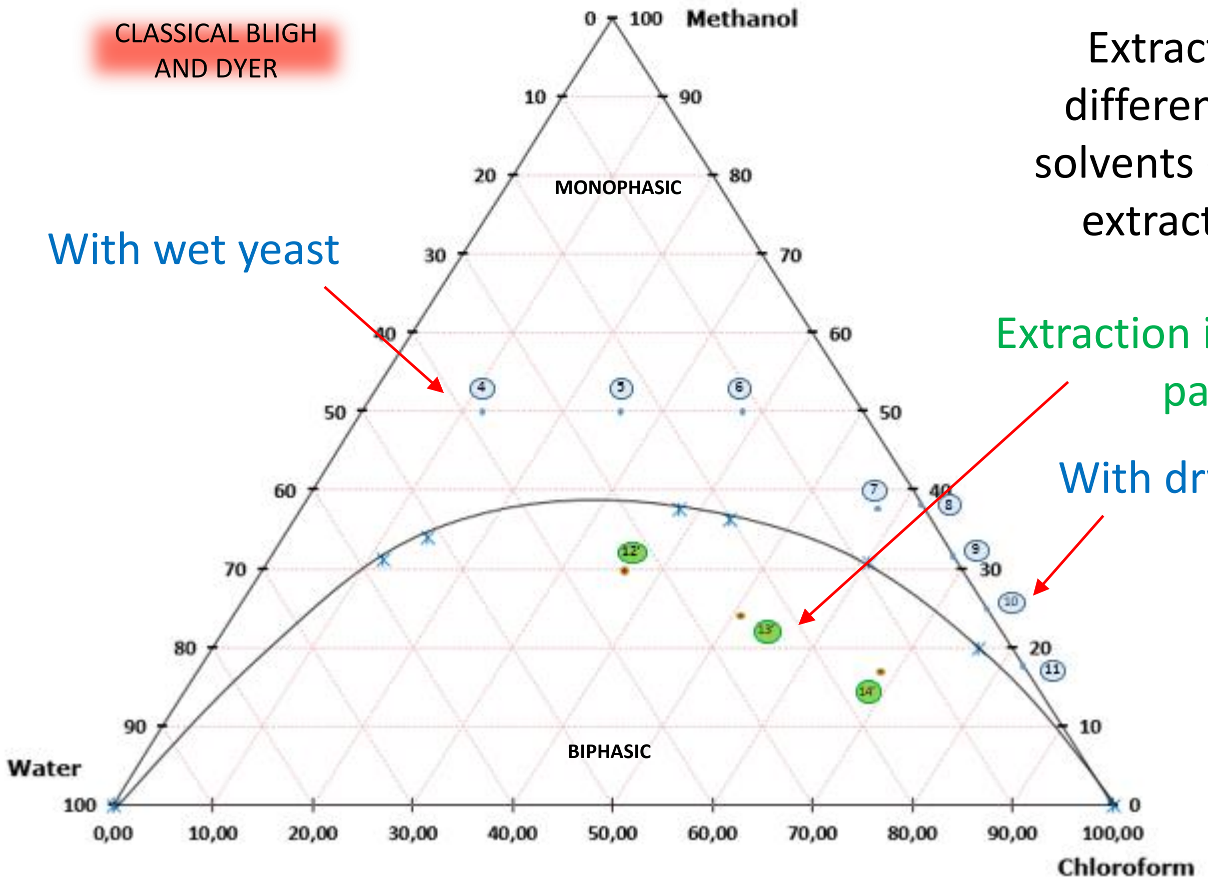

Extraction with different ratios of solvents (solid-liquid extraction step)

Extraction in biphasic part

With dry yeast

GREEN BLIGH AND DYER

With wet yeast

With dry yeast

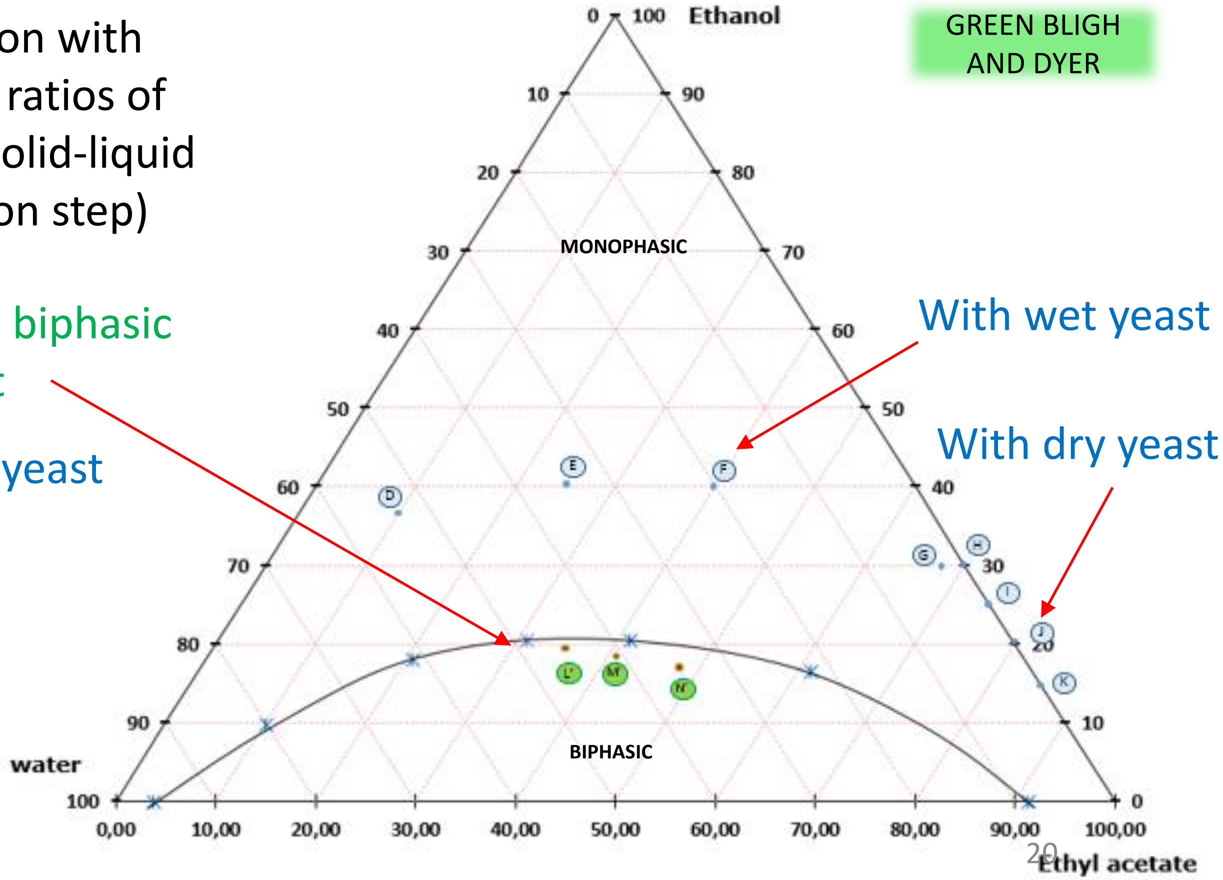

EXTRACTION PROCEDURE

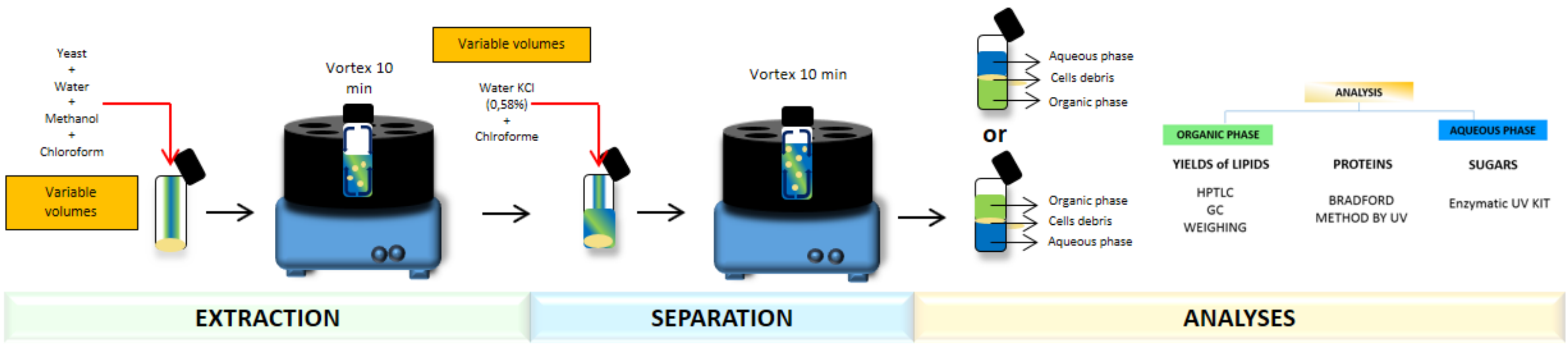

CLASSICAL BLIGH AND DYER

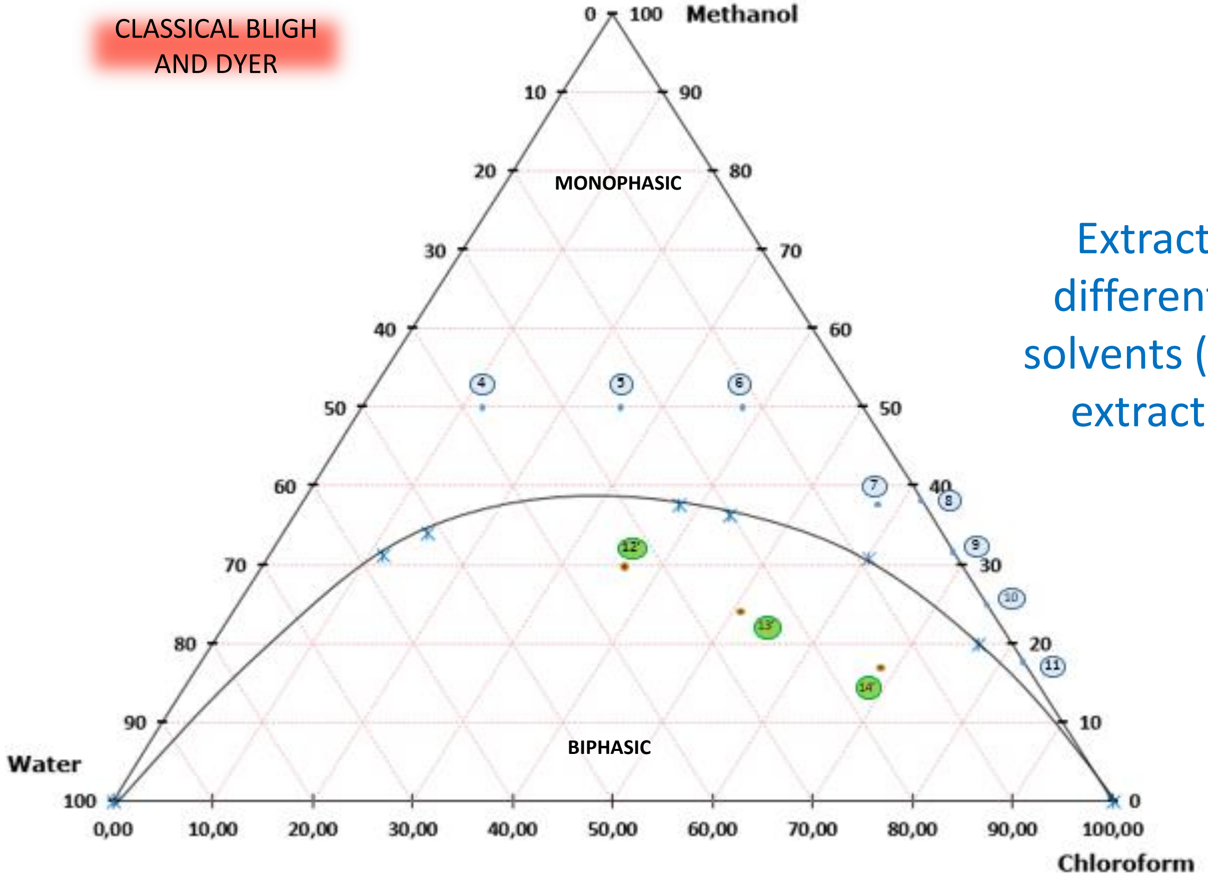

Extraction with different ratios of solvents (solid-liquid extraction step)

GREEN BLIGH AND DYER

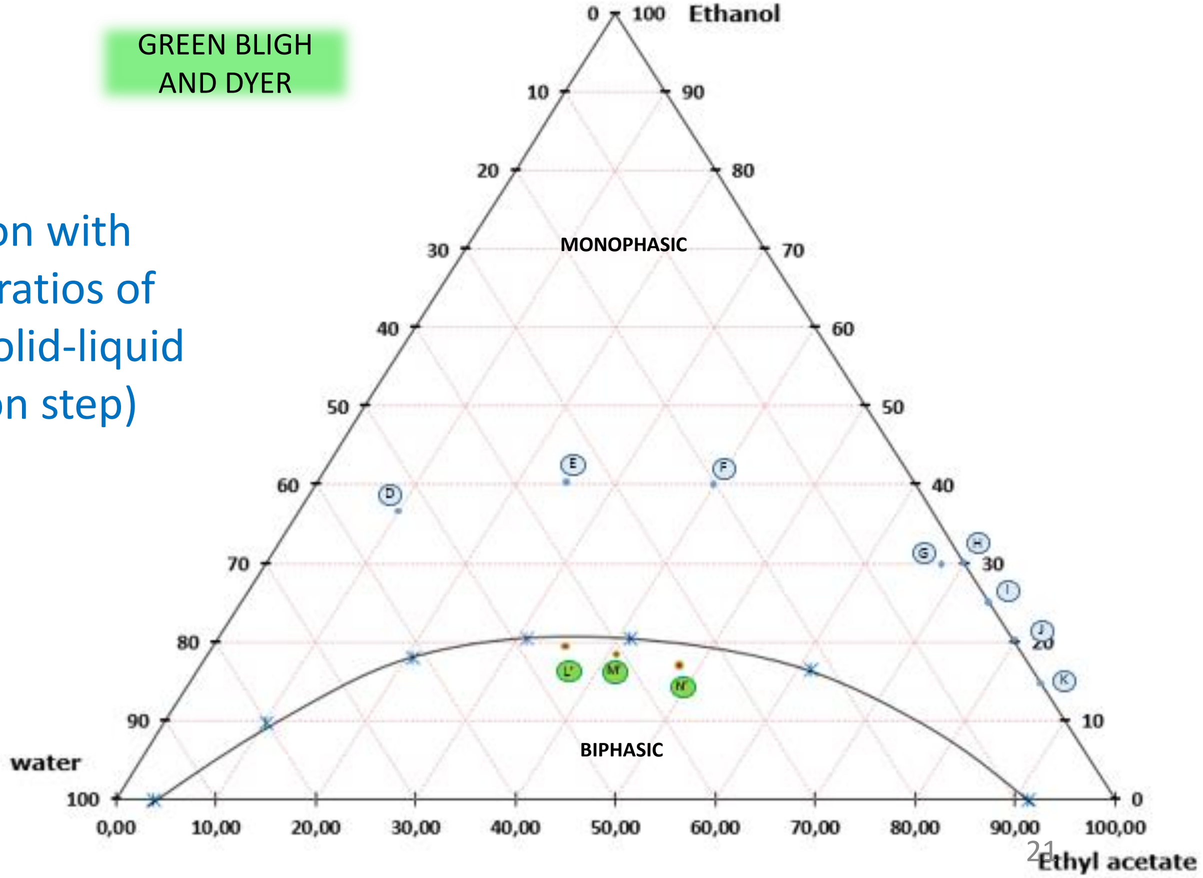

EXTRACTION PROCEDURE

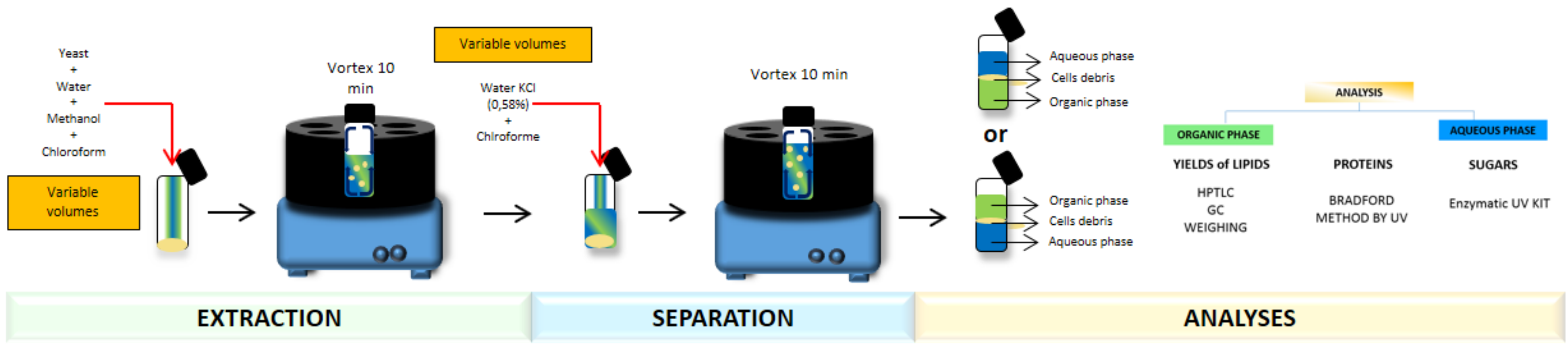

CLASSICAL BLIGH AND DYER

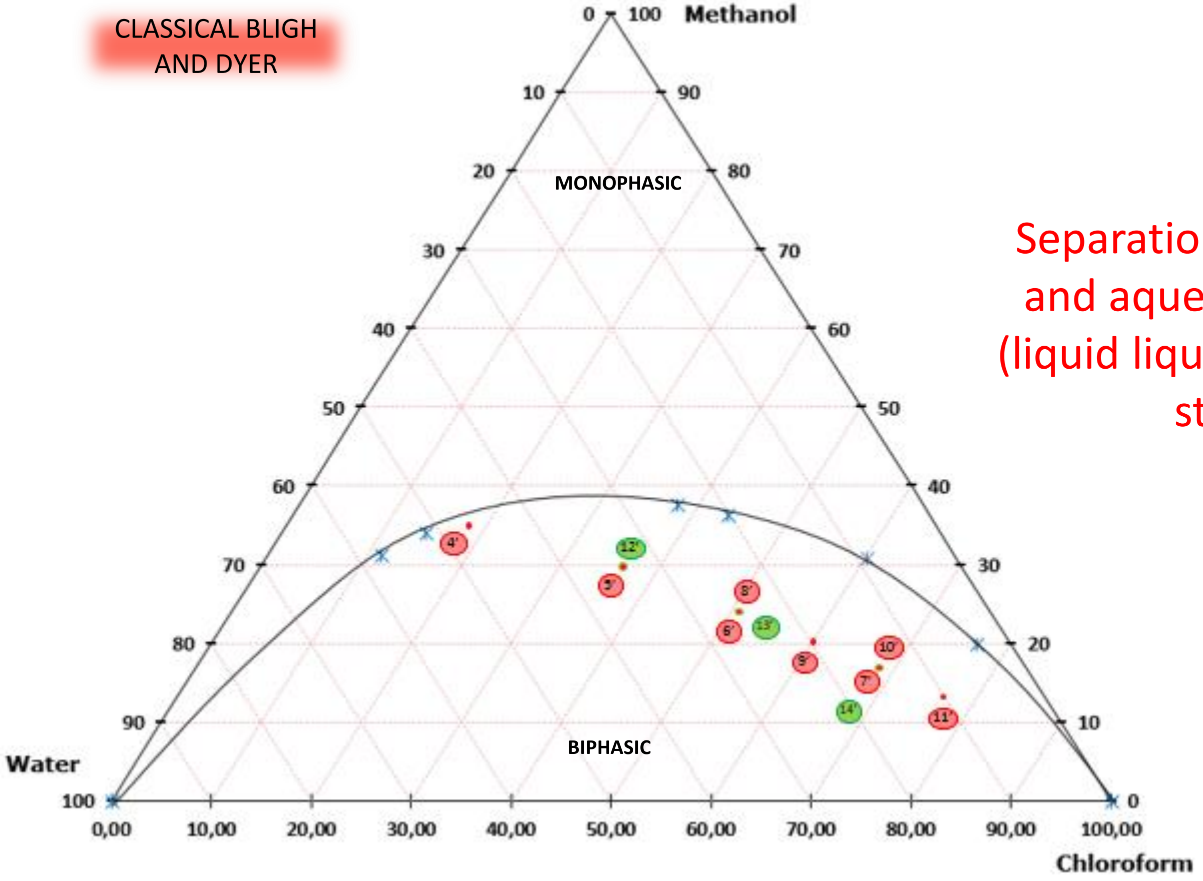

Separation of organic and aqueous phases (liquid liquid extraction step)

GREEN BLIGH AND DYER

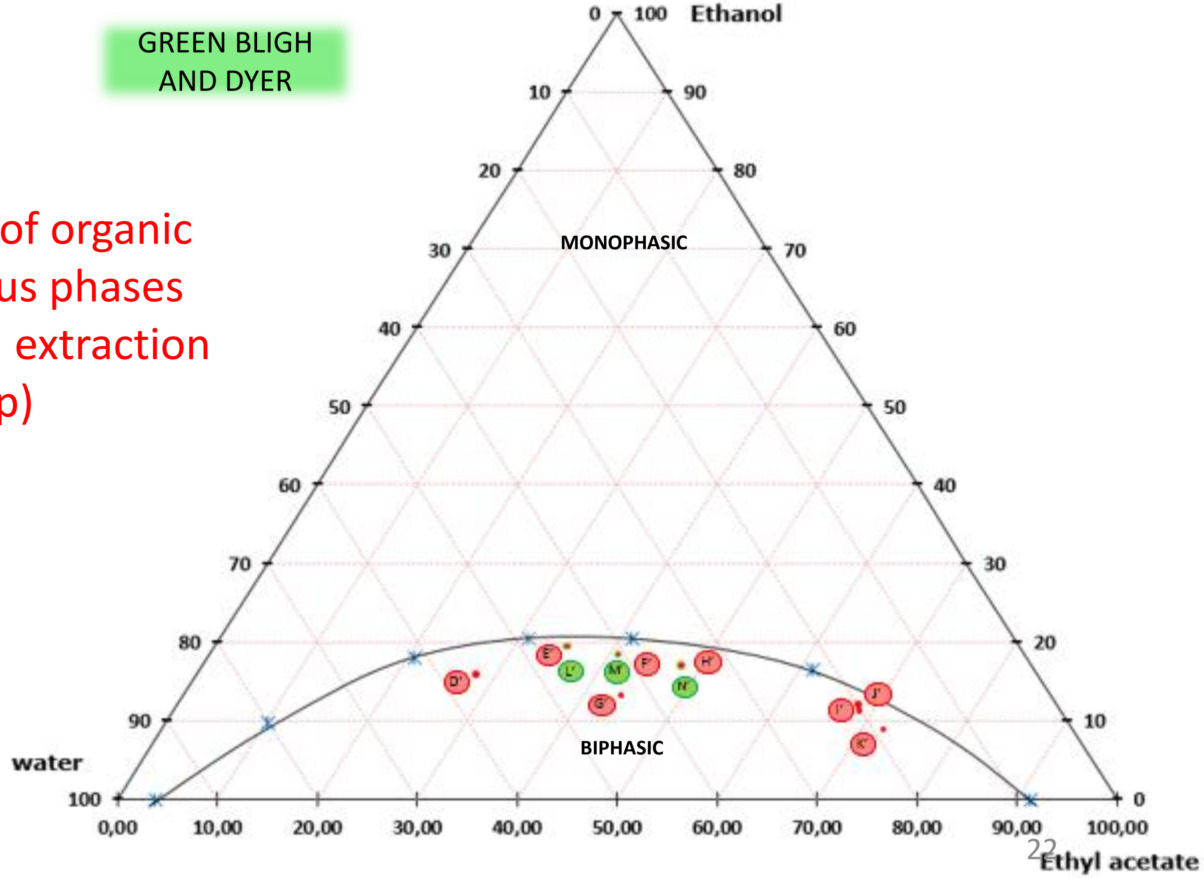

ANALYTICAL PROCEDURE FOR LIPIDS

GAS CHROMATOGRAPHY

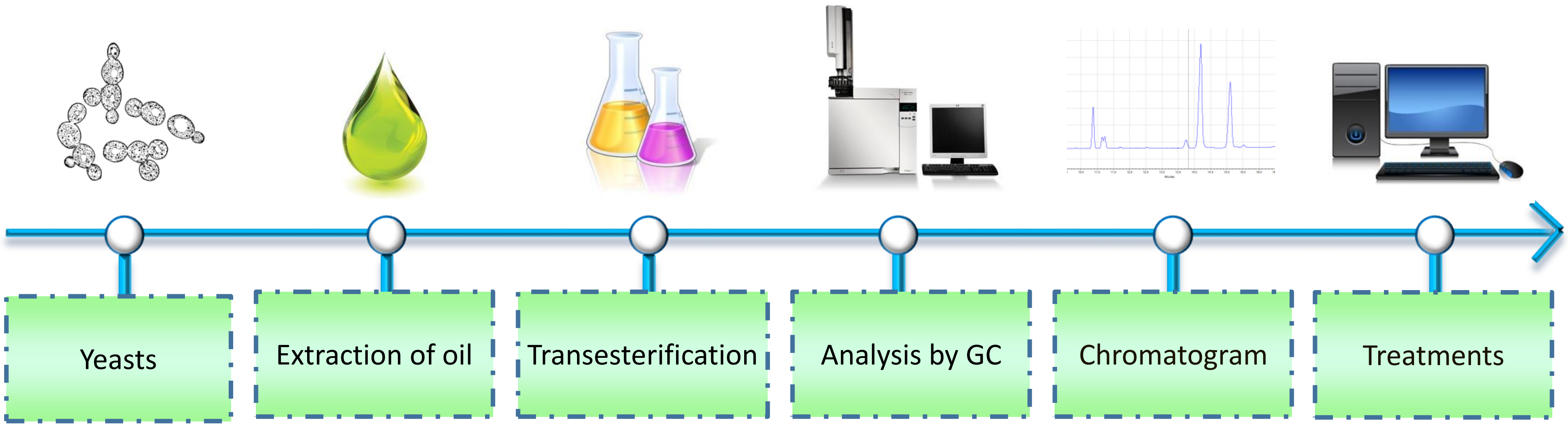

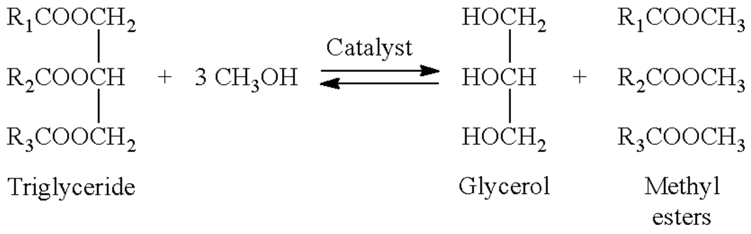

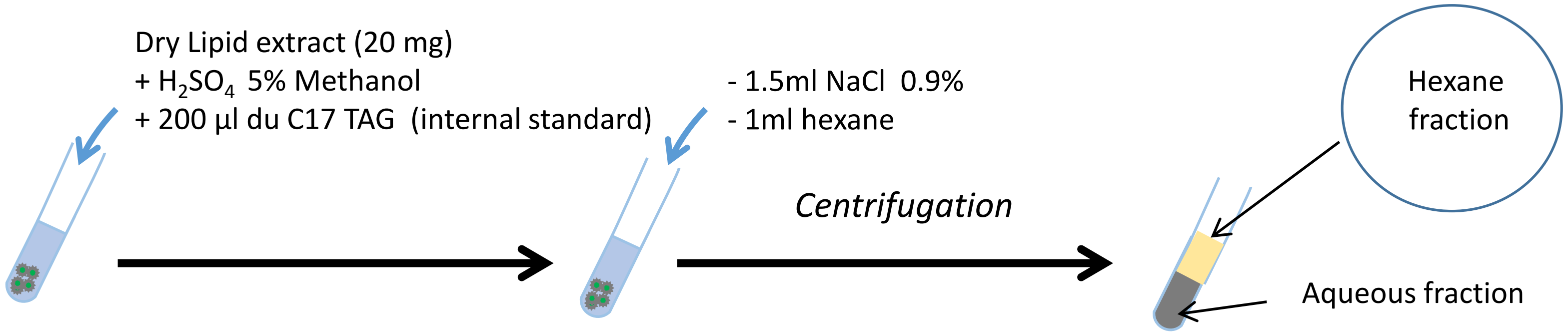

LIPIDS ANALYSIS OF BLIGH AND DYER

CLASSICAL BLIGH AND DYER

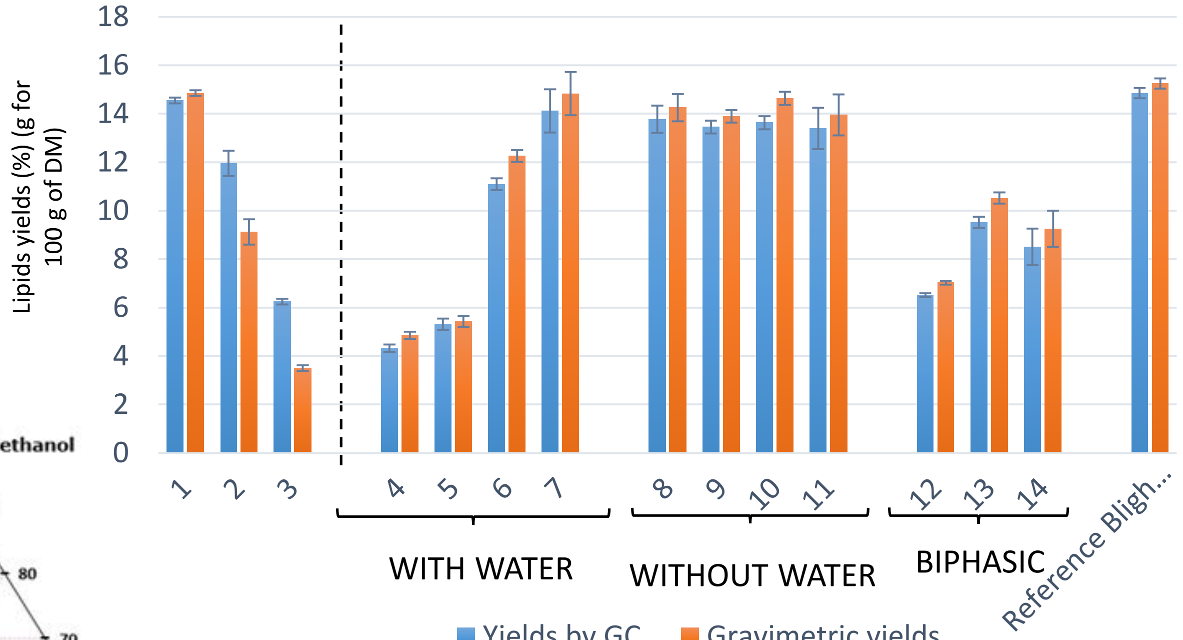

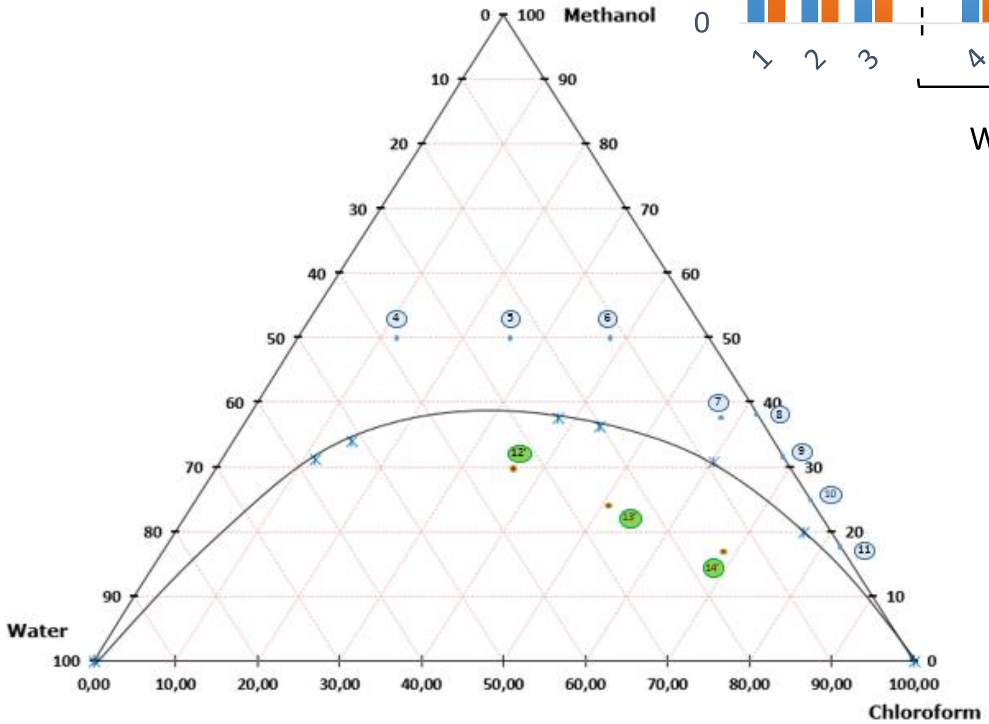

- Yields ↗ when the % of chloroform ↗
- % of chloroform in point 8 is sufficient to extract lipids
- Biphasic systems are not efficient

LIPIDS ANALYSIS OF BLIGH AND DYER

GREEN BLIGH AND DYER

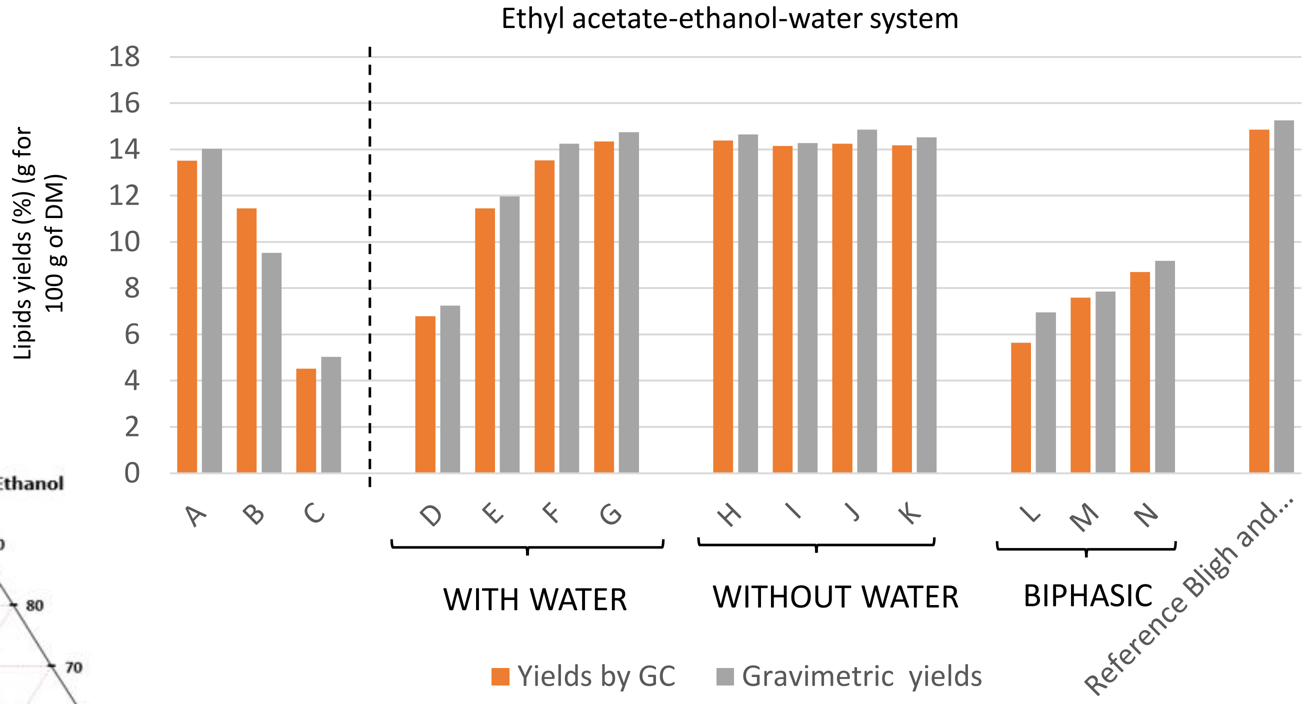

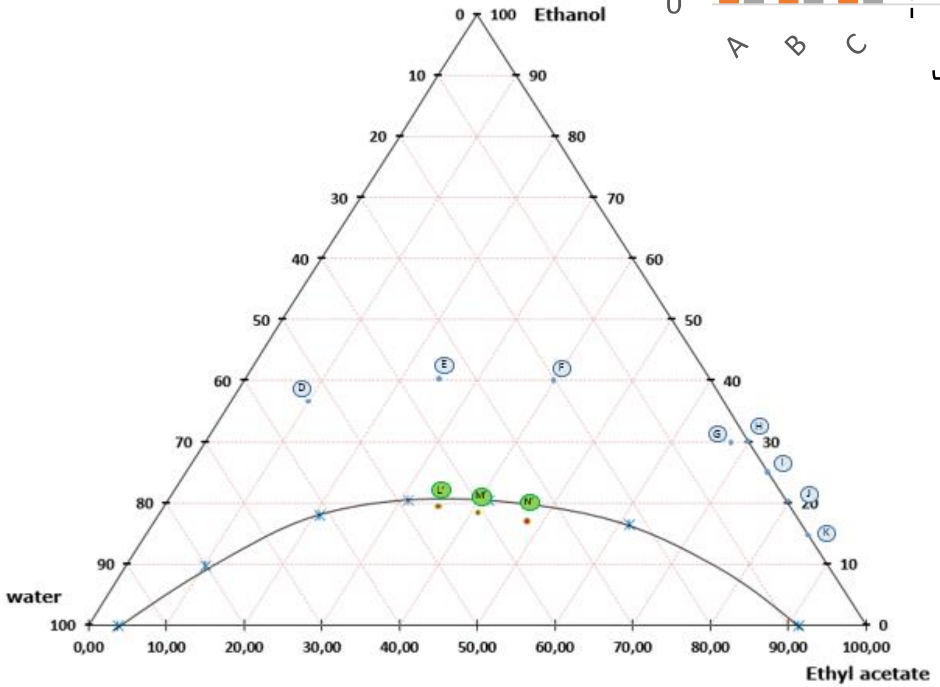

- Yields ↗ when the % of ethyl acetate ↗
- % of ethyl acetate in point H is sufficient to extract lipids
- Biphasic systems are not efficient

ANALYTICAL PROCEDURE

High Performance Thin Layer Chromatography

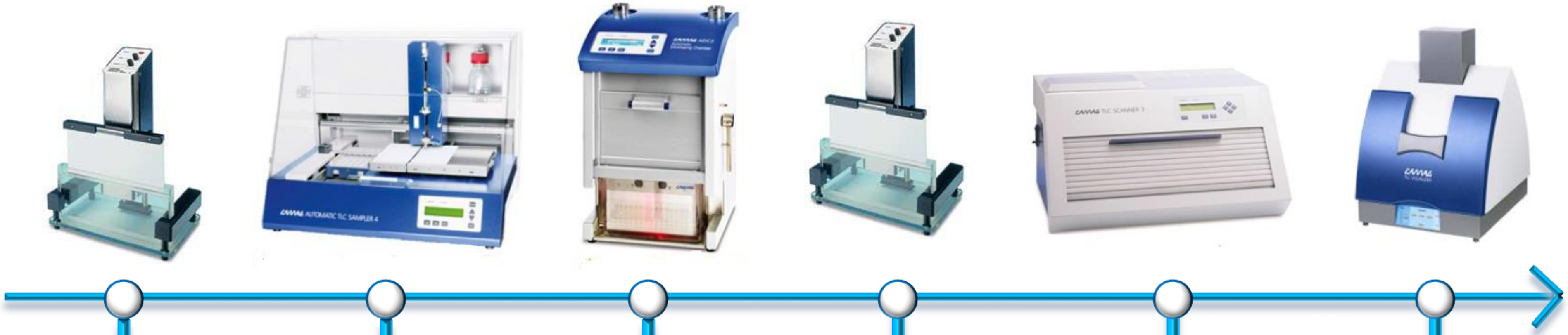

Conditioning of the plate	Automatic deposit	Automatic deposit	Revelation	Evaluation by densitometry	Picture and Data processing

SOLVENT
CHCl3/MeOH

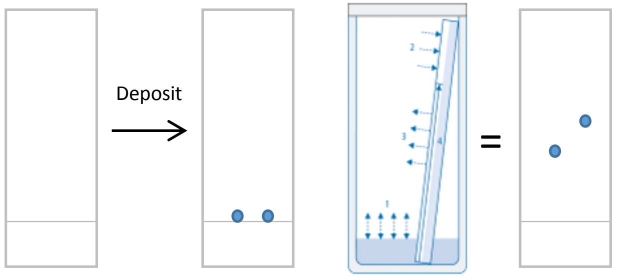

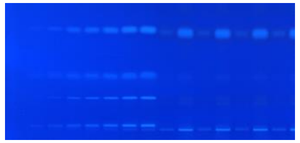

MAG, DAG, FFA, TAG

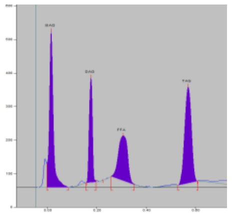

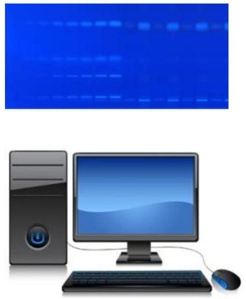

Cleaning and pretreatment of the plate	Automatic deposit of sample spot or strip	Solvent :<br>• Hexane<br>• Diethylether<br>• Acetic acid	Solvent :<br>• Primuline<br>• Acetone<br>• Eau	• Lamp : Hg<br>• λ : 366 nm	Computer data processing

ANALYTICAL PROCEDURE

High Performance Thin Layer Chromatography

CLASSICAL BLIGH & DYER

AQUEOUS PHASE

NEUTRAL LIPIDS

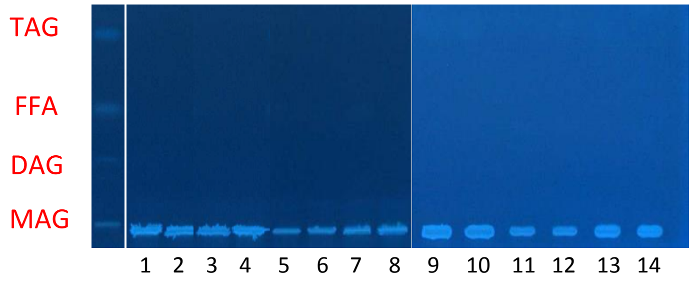

- No spot of lipids in aquous phase

POLAR LIPIDS

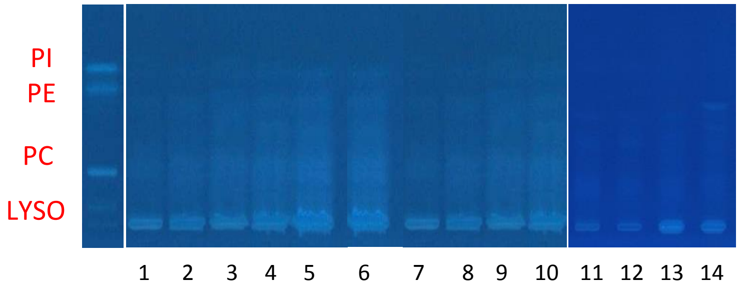

- No spot of lipids in aquous phase

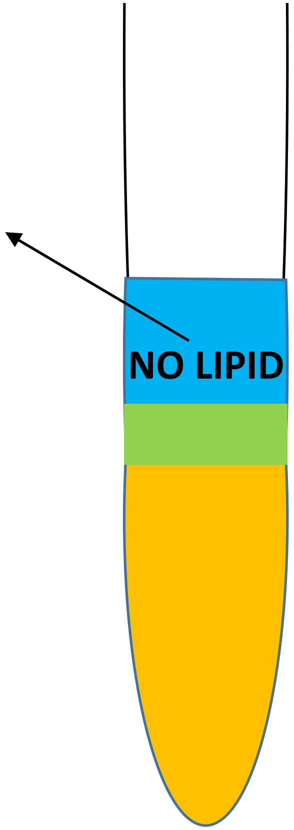

ANALYTICAL PROCEDURE

High Performance Thin Layer Chromatography

CLASSICAL BLIGH & DYER

AQUEOUS PHASE

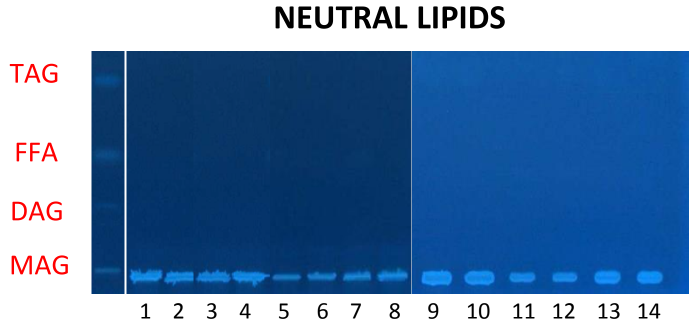

- No spot of lipids in aquous phase

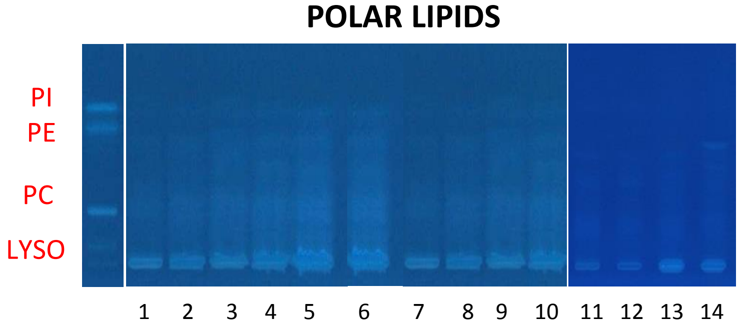

- No spot of lipids in aquous phase

ORGANIC PHASE

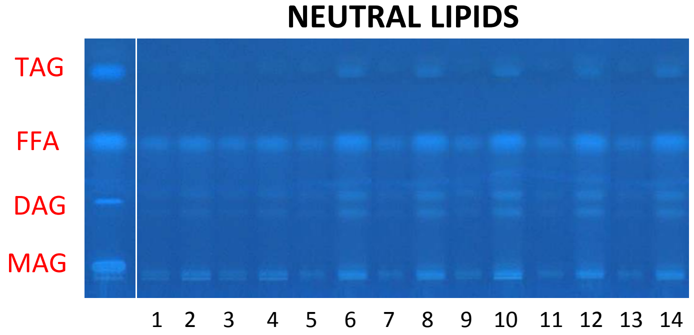

- Lipids present in organic phase
- Presence of sterols

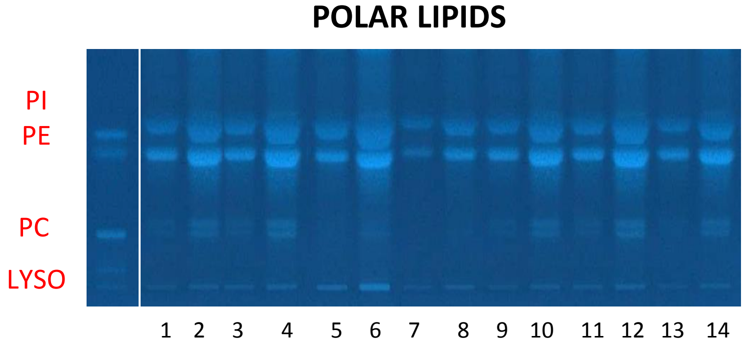

- Lipids present in organic phase

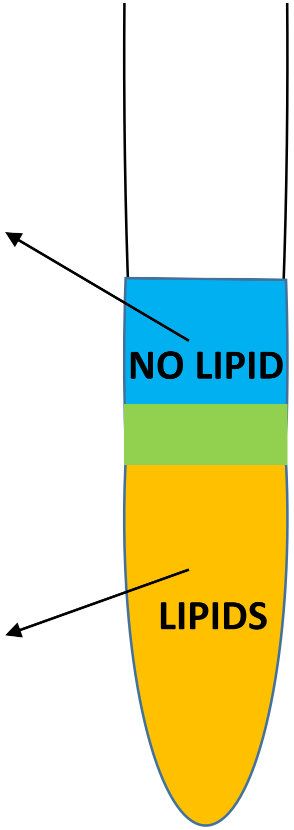

Classical system is efficient to extract total lipids in organic phases

ANALYTICAL PROCEDURE

High Performance Thin Layer Chromatography

GREEN BLIGH & DYER

ORGANIC PHASE

NEUTRAL LIPIDS

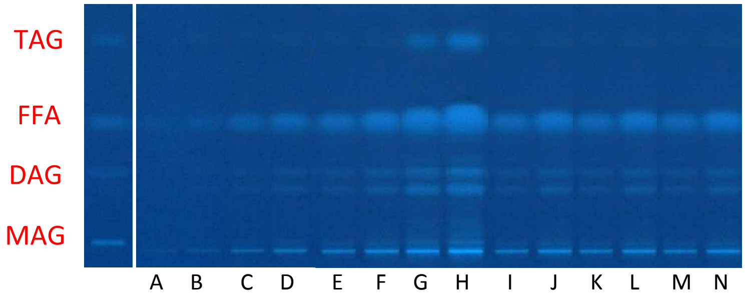

- Lipids present in organic phase
- Presence of sterols

POLAR LIPIDS

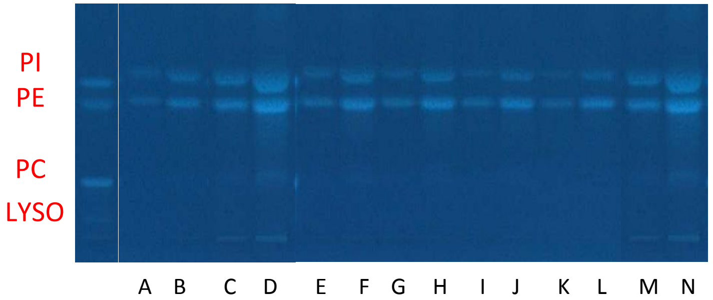

- Lipids present in organic phase

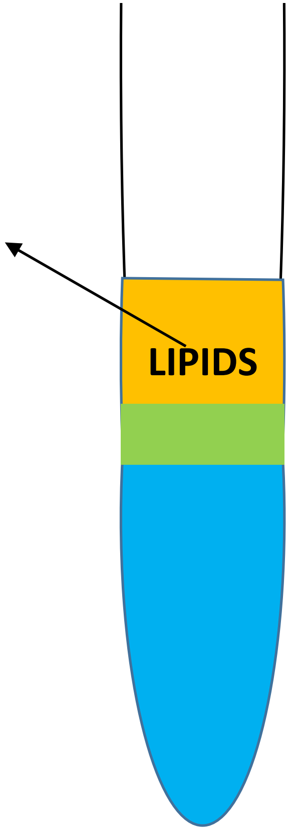

ANALYTICAL PROCEDURE

High Performance Thin Layer Chromatography

GREEN BLIGH & DYER

ORGANIC PHASE

NEUTRAL LIPIDS

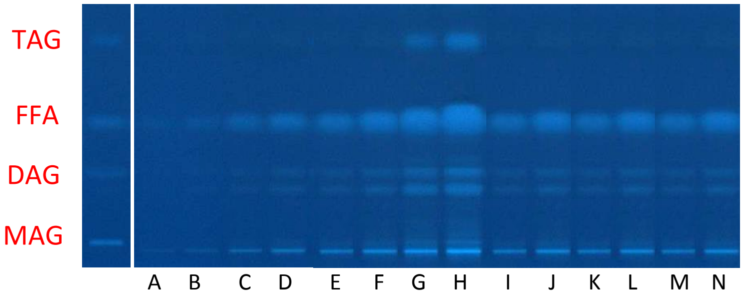

- Lipids present in organic phase
- Presence of sterols

POLAR LIPIDS

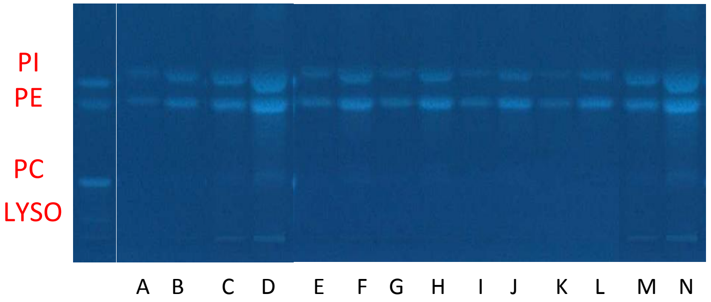

- Lipids present in organic phase

AQUEOUS PHASE

NEUTRAL LIPIDS

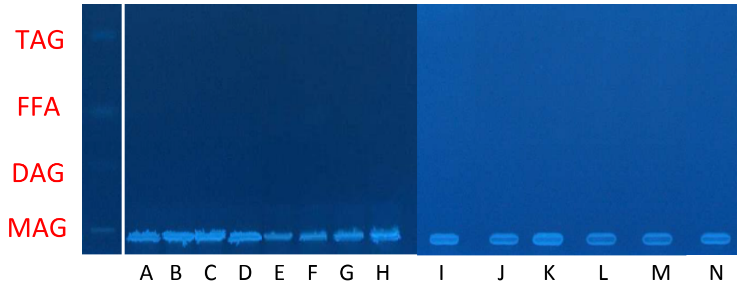

- No spot of lipids in aquous phase

POLAR LIPIDS

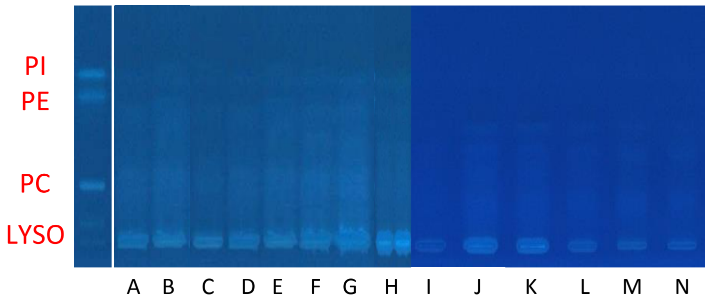

- No spot of lipids in aquous phase

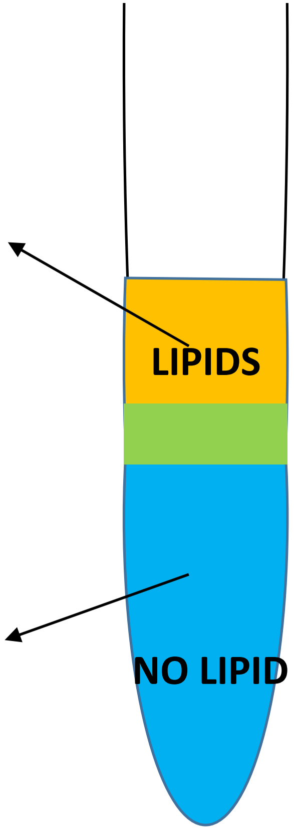

Green system is efficient to extract total lipids in organic phases

ANALYTICAL PROCEDURE

High Performance Thin Layer Chromatography

RELATIVE PROFIL OF LIPID CLASSES BY HPTLC

Analyses by HPTLC

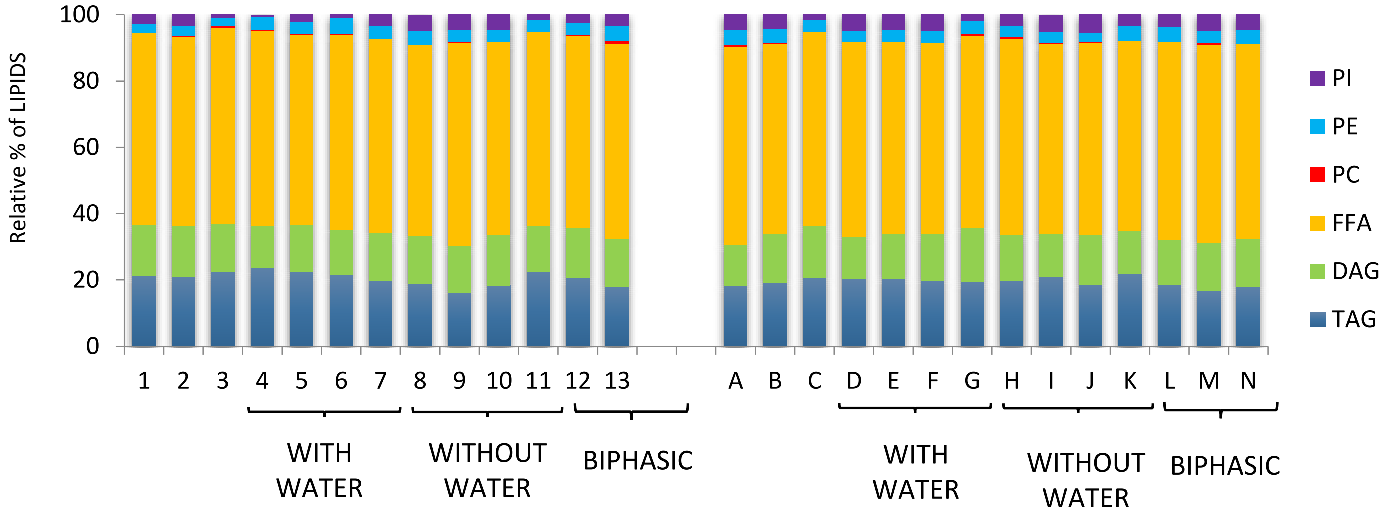

Mainly composed of FFA, TAG and DAG

No selectivity between extractions

RELATIVE PROFIL OF FATTY ACIDS BY GC

Analyses by GC

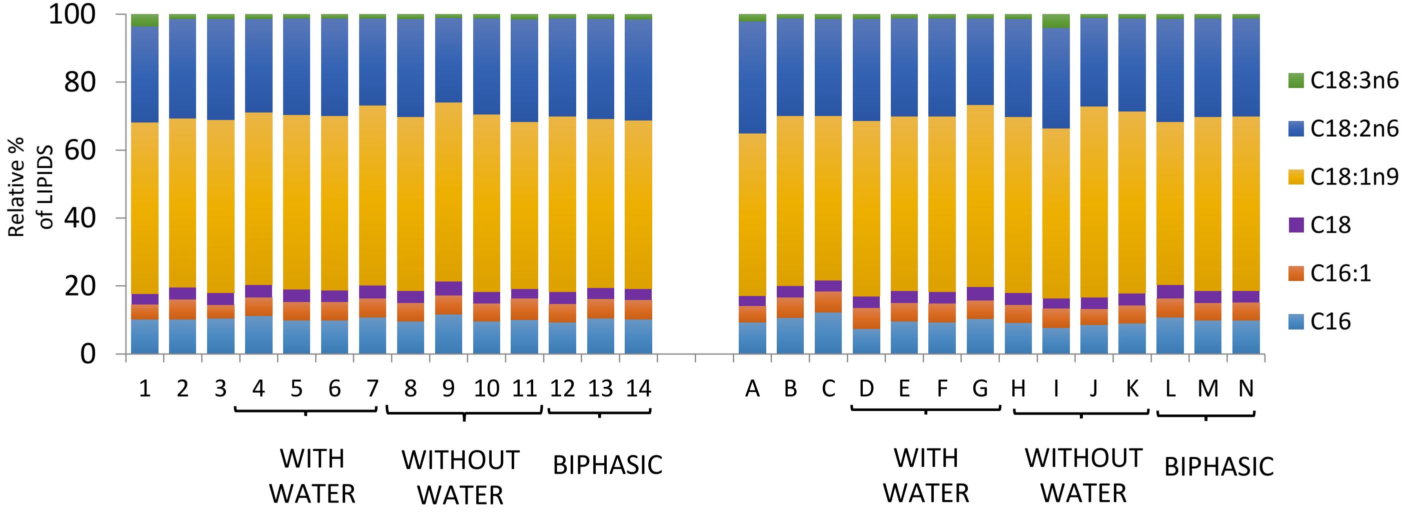

Mainly composed of oleic acid, linoleic acid and palmitic acid

No selectivity between extractions

ANALYTICAL PROCEDURE

PROTEINS AND SUGARS

GREEN BLIGH AND DYER

CLASSICAL BLIGH AND DYER

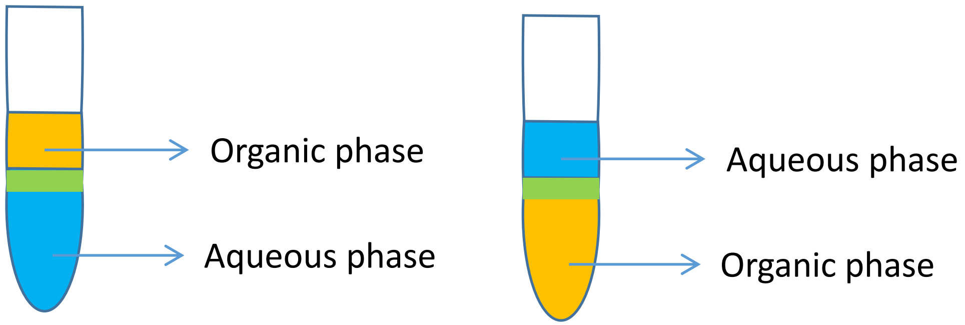

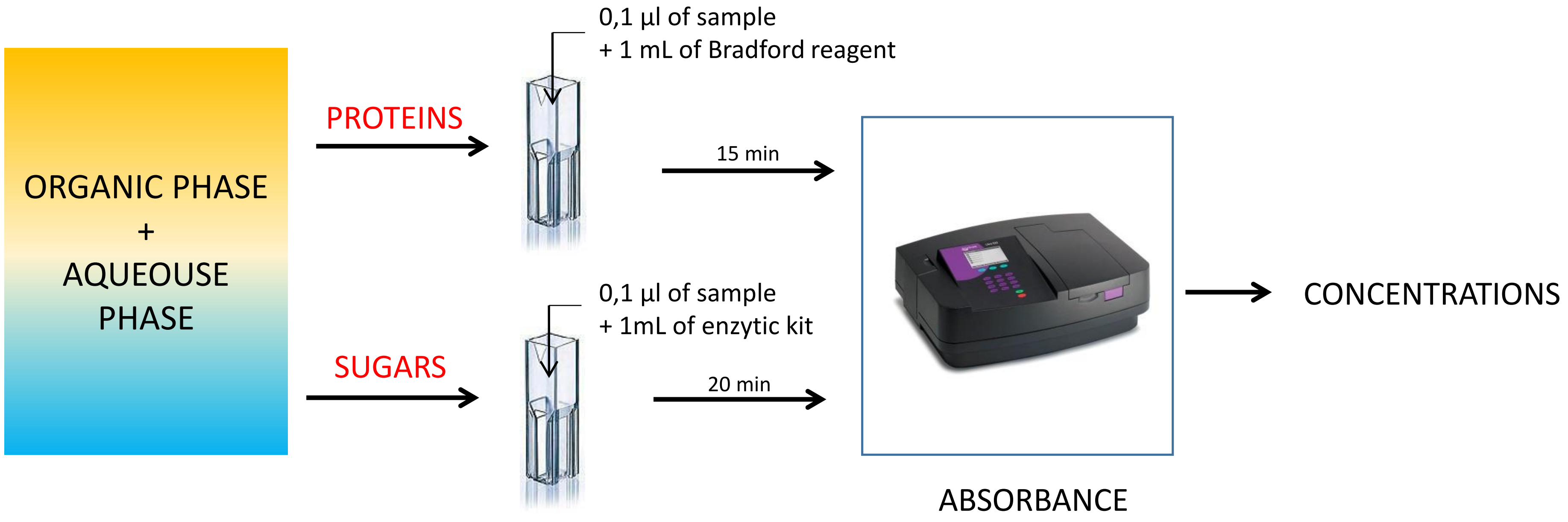

DETERMINATION OF PROTEINS BY BRADFORD METHOD

CLASSICAL BLIGH AND DYER

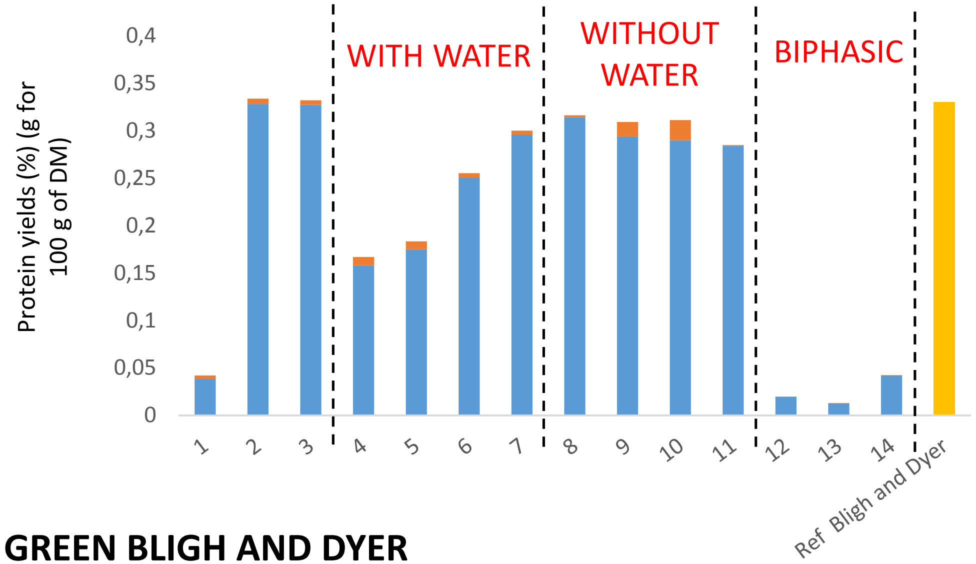

- Few proteins are present in the organic phases
- The quantity of proteins increased from points 4 to 7
- The quantity of proteins extracted is the same for points 8 to 11.
- Biphasic systems are not efficient.
- Chloroform doesn't extract proteins.

GREEN BLIGH AND DYER

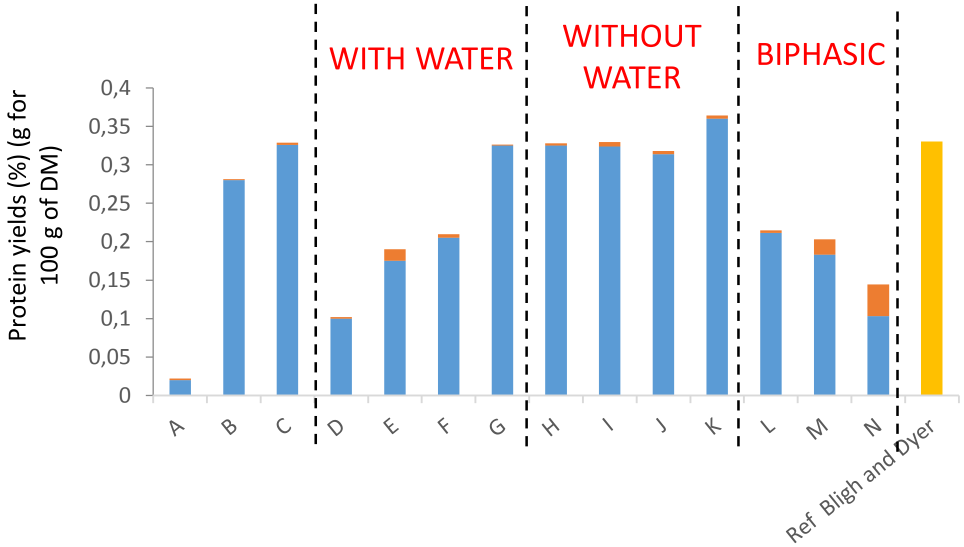

- Few proteins are present in the organic phases
- The quantity of proteins increased from points D to G
- The quantity of proteins extracted is the same for points H to K
- Biphasic systems are not efficient
- Ethyl acetate doesn't extract proteins

DETERMINATION OF GLUCOSE BY ENZYMATIC METHOD

CLASSICAL BLIGH AND DYER

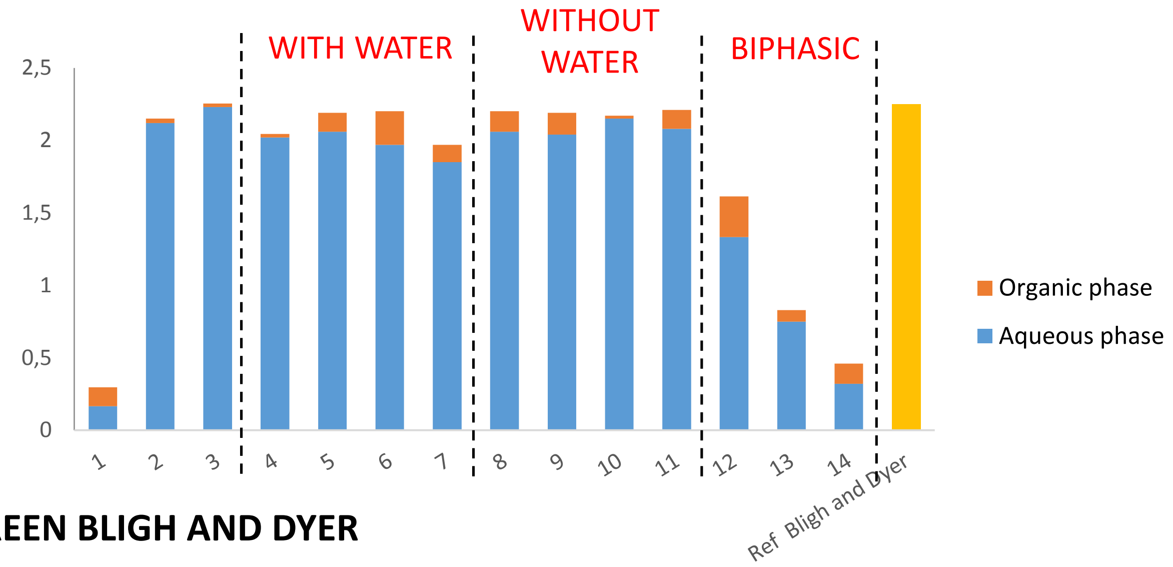

- Few sugars are present in the organic phases
- The quantity of sugars extracted is the same for points 4 to 11
- The quantities of methanol in all extractions are enough to extract all sugars
- Biphasic systems are not efficient
- Chloroform is not efficient

GREEN BLIGH AND DYER

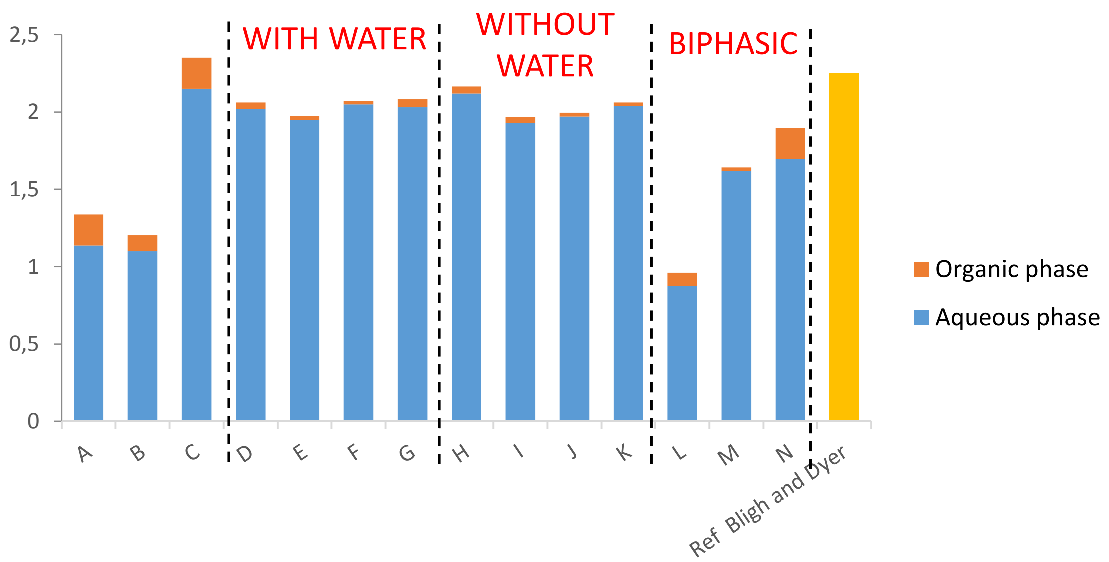

- Few sugars are present in the organic phases
- The quantity of sugars extracted is the same for points D to K
- The quantities of ethanol in all extractions are enough to extract all sugars
- Biphasic systems are not efficient

MODELIZATION PART
EXPERIMENTAL VS COSMO-RS

COMPARISON BETWEEN EXPERIMENTAL WITH COSMO-RS

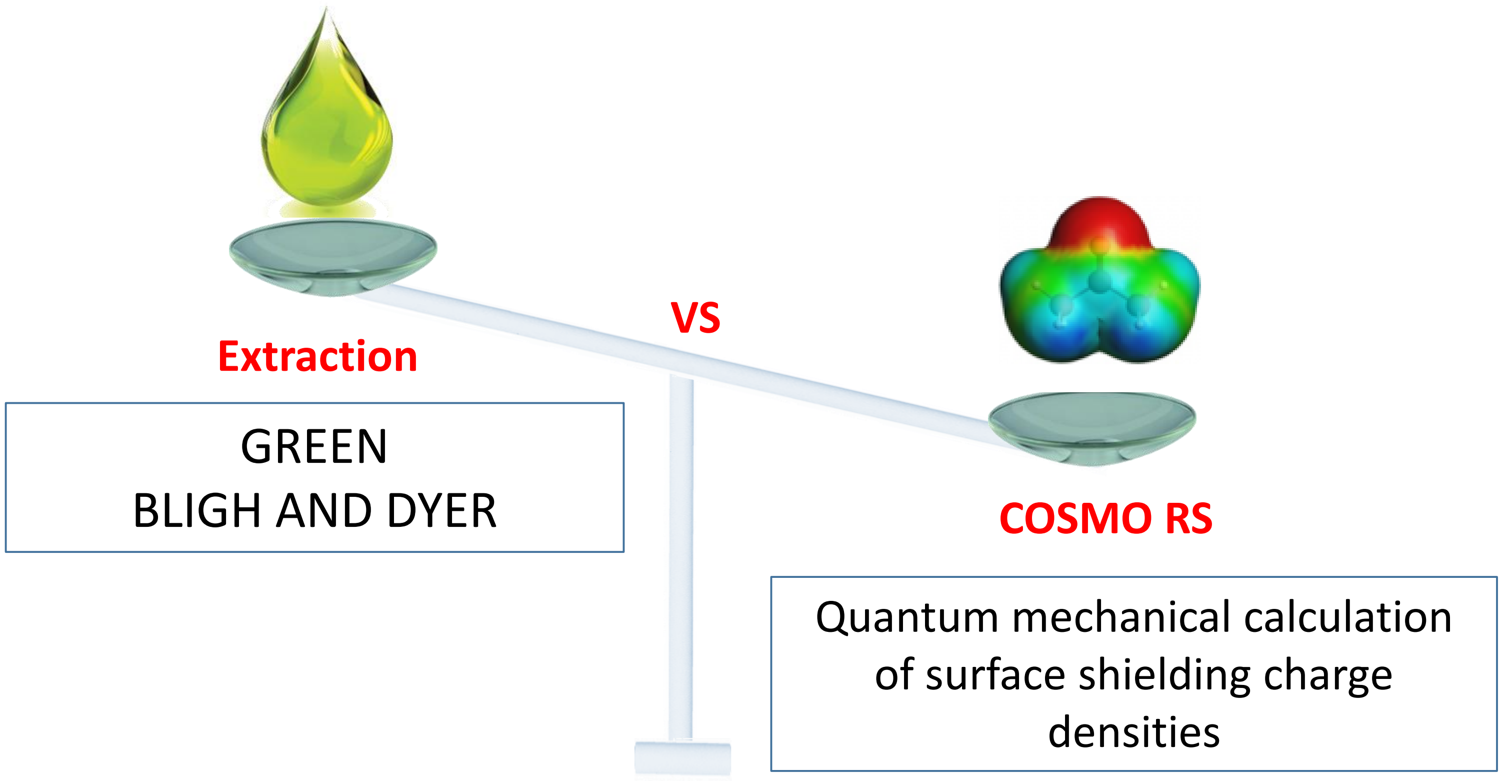

COSMO-RS VS EXPERIMENTAL

COSMO-RS Results

Wet biomass (row G)

	TAG LLL	TAG LOO	TAG OOO	DAG LGL	DAG LGO	DAG OGO	FFA181n9	FFA182n6	FFA 16	PC LL	PC OL	PE LL	PE OL	Lanosterol	Ergosterol	1,6bd glucan	1,4bd glucan	Chitine	Glycerol	Histidine	Arginine	Glucose
D	-9,8045	-10,1093	-10,0964	-5,6932	-7,1147	-6,3073	-2,9187	-2,7960	-2,7017	0,0000	0,0000	-4,7803	-4,6388	-4,0461	-3,5475	0,0000	0,0000	0,0000	0,0000	0,0000	0,0000	0,0000
E	-5,9882	-6,2811	-6,2806	-3,1411	-4,3326	-3,6557	-0,1416	-0,4542	-0,2268		0,0000	0,0000	-0,2826	-2,2974	0,0000	0,0000	0,0000	0,0000	0,0000	0,0000	0,0000	0,0000
F	-3,4403	-3,7745	-3,7767	-0,0879	-2,3168	-0,2306	-0,5897	-0,2510	-0,2827	0,0000	0,0000	-0,1642	-0,1834	0,0000	0,0000	0,0000	0,0000	0,0000	0,0000	0,0000	0,0000	0,0000
G	0,0000	0,0000	0,0000	0,0000	-0,3812	-0,0502	0,0000	0,0000	0,0000	0,0000	0,0000	0,0000	0,0000	-0,2064	-0,2721	0,0000	0,0000	0,0000	0,0000	0,0000	0,0000	0,0000
H	-0,0373	-0,0495	-0,0477	0,0000	-0,0698	0,0000	0,0000	0,0000	0,0000	0,0000	0,0000	0,0000	0,0000	-0,0410	0,0000	0,0000	0,0000	0,0000	0,0000	0,0000	0,0000	0,0000
I	0,0000	0,0000	-0,0943	-0,0819	0,0000	0,0000	-0,0317	0,0000	-0,0009	0,0000	0,0000	0,0000	0,0000	0,0000	0,0000	0,0000	0,0000	0,0000	0,0000	0,0000	0,0000	0,0000
J	0,0000	-0,2454	-0,1412	0,0000	0,0000	0,0000	0,0000	0,0000	0,0000	0,0000	0,0000	0,0000	0,0000	0,0000	0,0000	0,0000	0,0000	0,0000	0,0000	0,0000	0,0000	0,0000
K	0,0000	-0,0932	-0,0551	0,0000	-0,0698	0,0000	0,0000	0,0000	0,0000	0,0000	0,0000	0,0000	0,0000	-0,0410	0,0000	0,0000	0,0000	0,0000	0,0000	0,0000	0,0000	0,0000
L	-6,9698	-6,9677	-6,6309	-3,5914	-4,8259	-4,1463	-0,4332	-0,1857	-0,2656	0,0000	0,0000	-2,5101	-2,3435	-2,6500	-2,1338	0,0000	0,0000	0,0000	0,0000	0,0000	0,0000	0,0000
M	-4,8769	-4,8712	-4,5084	-0,1187	-3,2579	-2,5774	-0,4641	-0,2841	-0,3857	0,0000	0,0000	-0,1845	-0,1498	0,0000	0,0000	0,0000	0,0000	0,0000		-0,0790	0,0000	0,0000
N	-0,0272	0,0000	-0,0235	-0,4024	-0,0279	-0,2056	-0,0111	0,0000	-0,0080	0,0000	0,0000	0,0000	0,0000	-0,0430	0,0000	0,0000	0,0000	0,0000	0,0000	0,0000	0,0000	0,0000
Water	-23,9561	-24,5679	-24,5454	-15,0881	-17,2616	-16,2308	-8,1786	-7,9878	-7,6721	-7,8643	-5,9187	-14,5558	-14,1337	-10,0524	-9,1910	0,0000	-1,0140	-0,2142	0,0000	0,0000	0,0000	0,0000
Ethanol	-2,4067	-2,3964	-2,2586	0,0000	-0,0317	-0,0819	-0,0009	0,0000	0,0000	0,0000	-0,2683	-0,1585	0,0000	0,0000	0,0000	0,0000	0,0000	0,0000	0,0000	0,0000	0,0000	0,0000
Ethyl acetate	0,0000	0,0000	0,0000	0,0000	0,0000	0,0000	0,0000	0,0000	0,0000	0,0000	0,0000	0,0000	0,0000	0,0000	0,0000	-7,8643	-5,9187	-14,5558	-14,1337	-17,4794	-20,2240	0,0000
Water	-23,9561	-24,5679	-24,5454	-15,0881	-17,2616	-16,2308	-8,1786	-7,9878	-7,6721	-7,8643	-5,9187	-14,5558	-14,1337	-10,0524	-9,1910	0,0000	-1,0140	-0,2142	0,0000	0,0000	0,0000	0,0000

GREEN BLIGH AND DYER

Experimental part

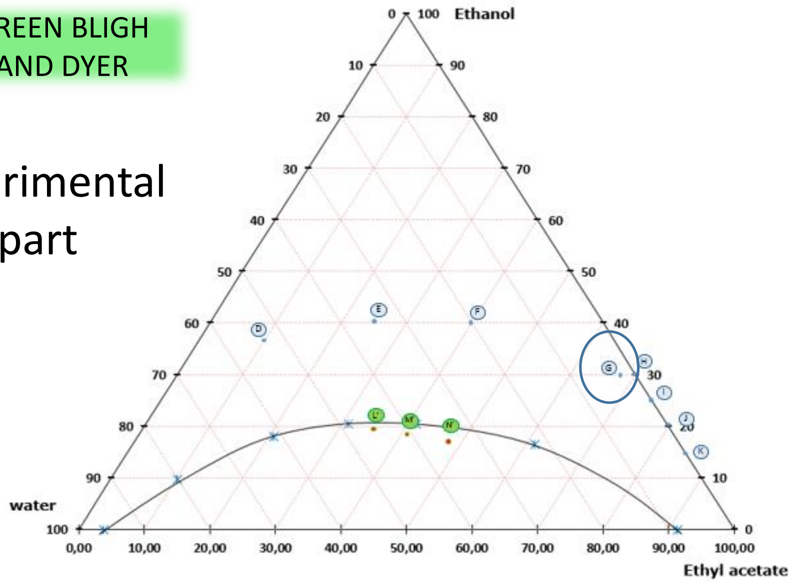

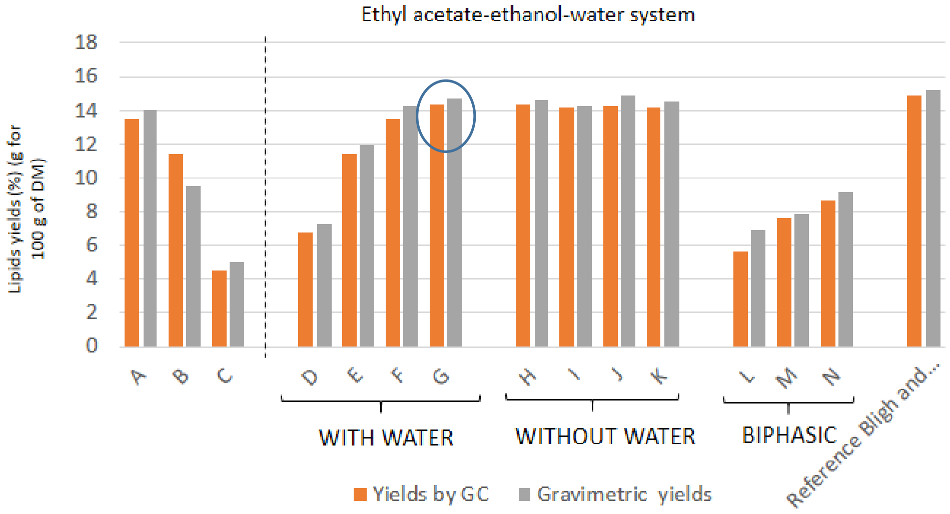

Theory is similar with the experiment

COSMO-RS VS EXPERIMENTAL

COSMO-RS Results

		TAG LLL	TAG LOO	TAG OOO	DAG LGL	DAG LGO	DAG OGO	FFA181n9	FFA182n6	FFA 16	PC LL	PC OL	PE LL	PE OL	Lanosterol	Ergosterol	1,6bd glucan	1,4bd glucan	Chitine	Glycerol	Histidine	Arginine	Glucose
	D	-9,8045	-10,1093	-10,0964	-5,6932	-7,1147	-6,3073	-2,9187	-2,7960	-2,7017	0,0000	0,0000	-4,7803	-4,6388	-4,0461	-3,5475	0,0000	0,0000	0,0000	0,0000	0,0000	0,0000	0,0000
	E	-5,9882	-6,2811	-6,2806	-3,1411	-4,3326	-3,6557	-0,1416	-0,4542	-0,2268		0,0000	0,0000	-0,2826	-2,2974	0,0000	0,0000	0,0000	0,0000	0,0000	0,0000	0,0000	0,0000
	F	-3,4403	-3,7745	-3,7767	-0,0879	-2,3168	-0,2306	-0,5897	-0,2510	-0,2827	0,0000	0,0000	-0,1642	-0,1834	0,0000	0,0000	0,0000	0,0000	0,0000	0,0000	0,0000	0,0000	0,0000
Wet biomass	G	0,0000	0,0000	0,0000	0,0000	-0,3812	-0,0502	0,0000	0,0000	0,0000	0,0000	0,0000	0,0000	0,0000	-0,2064	-0,2721	0,0000	0,0000	0,0000	0,0000	0,0000	0,0000	0,0000
Dry biomass	H	-0,0373	-0,0495	-0,0477	0,0000	-0,0698	0,0000	0,0000	0,0000	0,0000	0,0000	0,0000	0,0000	0,0000	-0,0410	0,0000	0,0000	0,0000	0,0000	0,0000	0,0000	0,0000	0,0000
Dry biomass	I	0,0000	0,0000	-0,0943	-0,0819	0,0000	0,0000	-0,0317	0,0000	-0,0009	0,0000	0,0000	0,0000	0,0000	0,0000	0,0000	0,0000	0,0000	0,0000	0,0000	0,0000	0,0000	0,0000
Dry biomass	J	0,0000	-0,2454	-0,1412	0,0000	0,0000	0,0000	0,0000	0,0000	0,0000	0,0000	0,0000	0,0000	0,0000	0,0000	0,0000	0,0000	0,0000	0,0000	0,0000	0,0000	0,0000	0,0000
Dry biomass	K	0,0000	-0,0932	-0,0551	0,0000	-0,0698	0,0000	0,0000	0,0000	0,0000	0,0000	0,0000	0,0000	0,0000	-0,0410	0,0000	0,0000	0,0000	0,0000	0,0000	0,0000	0,0000	0,0000
	L	-6,9698	-6,9677	-6,6309	-3,5914	-4,8259	-4,1463	-0,4332	-0,1857	-0,2656	0,0000	0,0000	-2,5101	-2,3435	-2,6500	-2,1338	0,0000	0,0000	0,0000	0,0000	0,0000	0,0000	0,0000
	M	-4,8769	-4,8712	-4,5084	-0,1187	-3,2579	-2,5774	-0,4641	-0,2841	-0,3857	0,0000	0,0000	-0,1845	-0,1498	0,0000	0,0000	0,0000	0,0000	0,0000		-0,0790	0,0000	0,0000
	N	-0,0272	0,0000	-0,0235	-0,4024	-0,0279	-0,2056	-0,0111	0,0000	-0,0080	0,0000	0,0000	0,0000	0,0000	-0,0430	0,0000	0,0000	0,0000	0,0000	0,0000	0,0000	0,0000	0,0000
	Water	-23,9561	-24,5679	-24,5454	-15,0881	-17,2616	-16,2308	-8,1786	-7,9878	-7,6721	-7,8643	-5,9187	-14,5558	-14,1337	-10,0524	-9,1910	0,0000	-1,0140	-0,2142	0,0000	0,0000	0,0000	0,0000
	Ethanol	-2,4067	-2,3964	-2,2586	0,0000	-0,0317	-0,0819	-0,0009	0,0000	0,0000	0,0000	-0,2683	-0,1585	0,0000	0,0000	0,0000	0,0000	0,0000	0,0000	0,0000	0,0000	0,0000	0,0000
	Ethyl acetate	0,0000	0,0000	0,0000	0,0000	0,0000	0,0000	0,0000	0,0000	0,0000	0,0000	0,0000	0,0000	0,0000	0,0000	0,0000	-7,8643	-5,9187	-14,5558	-14,1337	-17,4794	-20,2240	0,0000
	Water	-23,9561	-24,5679	-24,5454	-15,0881	-17,2616	-16,2308	-8,1786	-7,9878	-7,6721	-7,8643	-5,9187	-14,5558	-14,1337	-10,0524	-9,1910	0,0000	-1,0140	-0,2142	0,0000	0,0000	0,0000	0,0000

GREEN BLIGH AND DYER

Experimental part

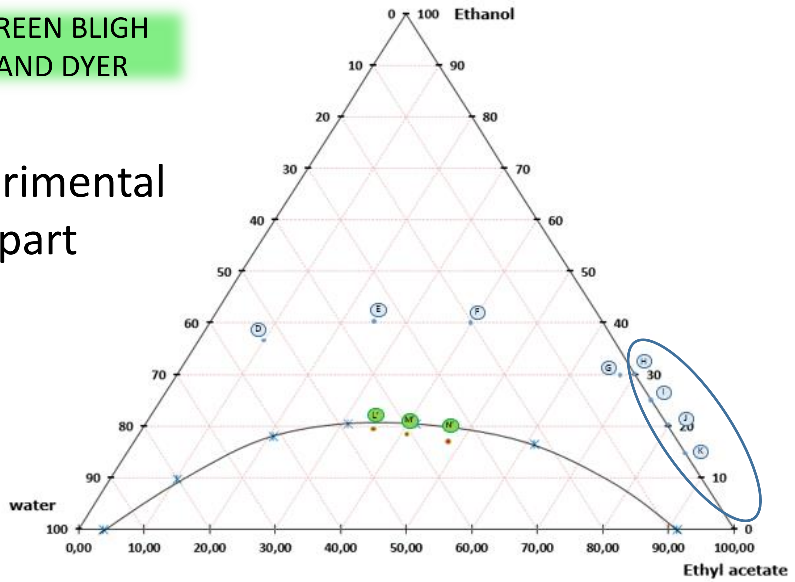

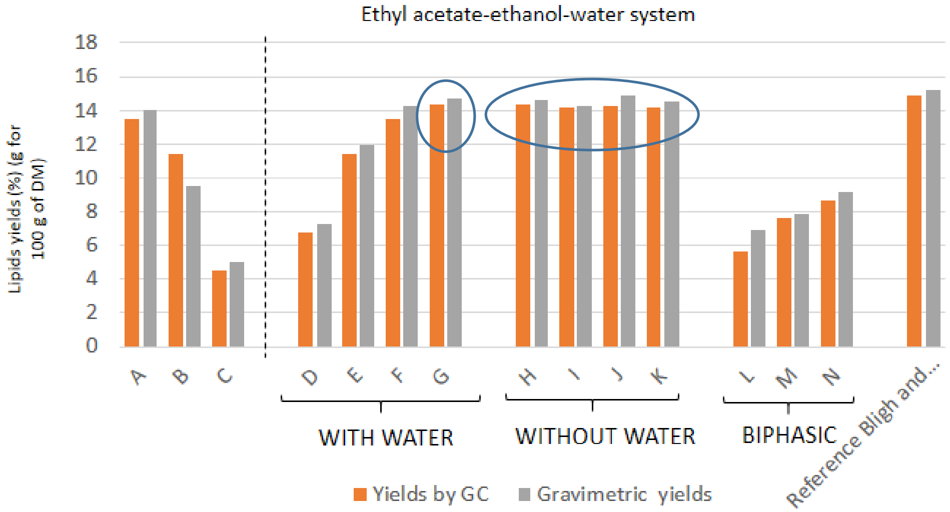

Theory is similar with the experiment

COSMO-RS VS EXPERIMENTAL

COSMO-RS Results

		TAG LLL	TAG LOO	TAG OOO	DAG LGL	DAG LGO	DAG OGO	FFA181n9	FFA182n6	FFA 16	PC LL	PC OL	PE LL	PE OL	Lanosterol	Ergosterol	1,6bd glucan	1,4bd glucan	Chitine	Glycerol	Histidine	Arginine	Glucose
	D	-9,8045	-10,1093	-10,0964	-5,6932	-7,1147	-6,3073	-2,9187	-2,7960	-2,7017	0,0000	0,0000	-4,7803	-4,6388	-4,0461	-3,5475	0,0000	0,0000	0,0000	0,0000	0,0000	0,0000	0,0000
	E	-5,9882	-6,2811	-6,2806	-3,1411	-4,3326	-3,6557	-0,1416	-0,4542	-0,2268		0,0000	0,0000	-0,2826	-2,2974	0,0000	0,0000	0,0000	0,0000	0,0000	0,0000	0,0000	0,0000
	F	-3,4403	-3,7745	-3,7767	-0,0879	-2,3168	-0,2306	-0,5897	-0,2510	-0,2827	0,0000	0,0000	-0,1642	-0,1834	0,0000	0,0000	0,0000	0,0000	0,0000	0,0000	0,0000	0,0000	0,0000
Wet biomass	G	0,0000	0,0000	0,0000	0,0000	-0,3812	-0,0502	0,0000	0,0000	0,0000	0,0000	0,0000	0,0000	0,0000	-0,2064	-0,2721	0,0000	0,0000	0,0000	0,0000	0,0000	0,0000	0,0000
Dry biomass	H	-0,0373	-0,0495	-0,0477	0,0000	-0,0698	0,0000	0,0000	0,0000	0,0000	0,0000	0,0000	0,0000	0,0000	-0,0410	0,0000	0,0000	0,0000	0,0000	0,0000	0,0000	0,0000	0,0000
	I	0,0000	0,0000	-0,0943	-0,0819	0,0000	0,0000	-0,0317	0,0000	-0,0009	0,0000	0,0000	0,0000	0,0000	0,0000	0,0000	0,0000	0,0000	0,0000	0,0000	0,0000	0,0000	0,0000
	J	0,0000	-0,2454	-0,1412	0,0000	0,0000	0,0000	0,0000	0,0000	0,0000	0,0000	0,0000	0,0000	0,0000	0,0000	0,0000	0,0000	0,0000	0,0000	0,0000	0,0000	0,0000	0,0000
	K	0,0000	-0,0932	-0,0551	0,0000	-0,0698	0,0000	0,0000	0,0000	0,0000	0,0000	0,0000	0,0000	0,0000	-0,0410	0,0000	0,0000	0,0000	0,0000	0,0000	0,0000	0,0000	0,0000
Extraction in biphasic part	L	-6,9698	-6,9677	-6,6309	-3,5914	-4,8259	-4,1463	-0,4332	-0,1857	-0,2656	0,0000	0,0000	-2,5101	-2,3435	-2,6500	-2,1338	0,0000	0,0000	0,0000	0,0000	0,0000	0,0000	0,0000
	M	-4,8769	-4,8712	-4,5084	-0,1187	-3,2579	-2,5774	-0,4641	-0,2841	-0,3857	0,0000	0,0000	-0,1845	-0,1498	0,0000	0,0000	0,0000	0,0000	0,0000		-0,0790	0,0000	0,0000
	N	-0,0272	0,0000	-0,0235	-0,4024	-0,0279	-0,2056	-0,0111	0,0000	-0,0080	0,0000	0,0000	0,0000	0,0000	-0,0430	0,0000	0,0000	0,0000	0,0000	0,0000	0,0000	0,0000	0,0000
	Water	-23,9561	-24,5679	-24,5454	-15,0881	-17,2616	-16,2308	-8,1786	-7,9878	-7,6721	-7,8643	-5,9187	-14,5558	-14,1337	-10,0524	-9,1910	0,0000	-1,0140	-0,2142	0,0000	0,0000	0,0000	0,0000
	Ethanol	-2,4067	-2,3964	-2,2586	0,0000	-0,0317	-0,0819	-0,0009	0,0000	0,0000	0,0000	-0,2683	-0,1585	0,0000	0,0000	0,0000	0,0000	0,0000	0,0000	0,0000	0,0000	0,0000	0,0000
	Ethyl acetate	0,0000	0,0000	0,0000	0,0000	0,0000	0,0000	0,0000	0,0000	0,0000	0,0000	0,0000	0,0000	0,0000	0,0000	0,0000	-7,8643	-5,9187	-14,5558	-14,1337	-17,4794	-20,2240	0,0000
	Water	-23,9561	-24,5679	-24,5454	-15,0881	-17,2616	-16,2308	-8,1786	-7,9878	-7,6721	-7,8643	-5,9187	-14,5558	-14,1337	-10,0524	-9,1910	0,0000	-1,0140	-0,2142	0,0000	0,0000	0,0000	0,0000

GREEN BLIGH AND DYER

Experimental part

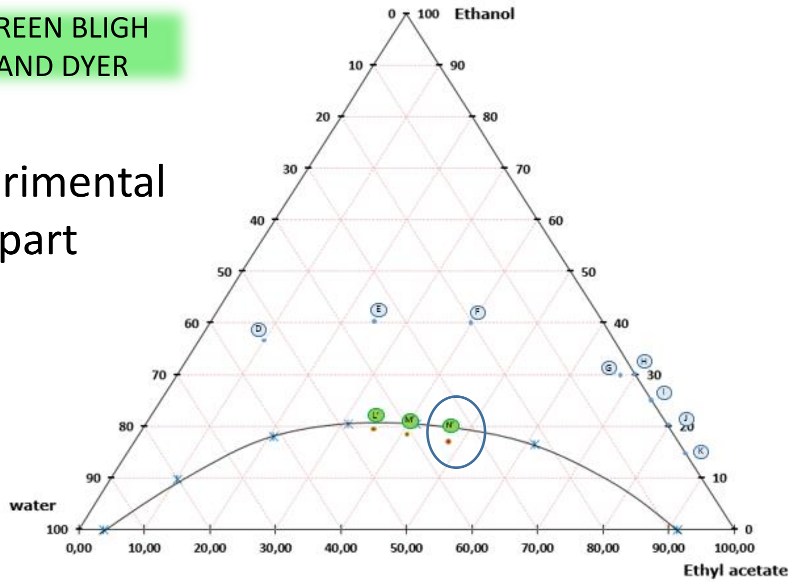

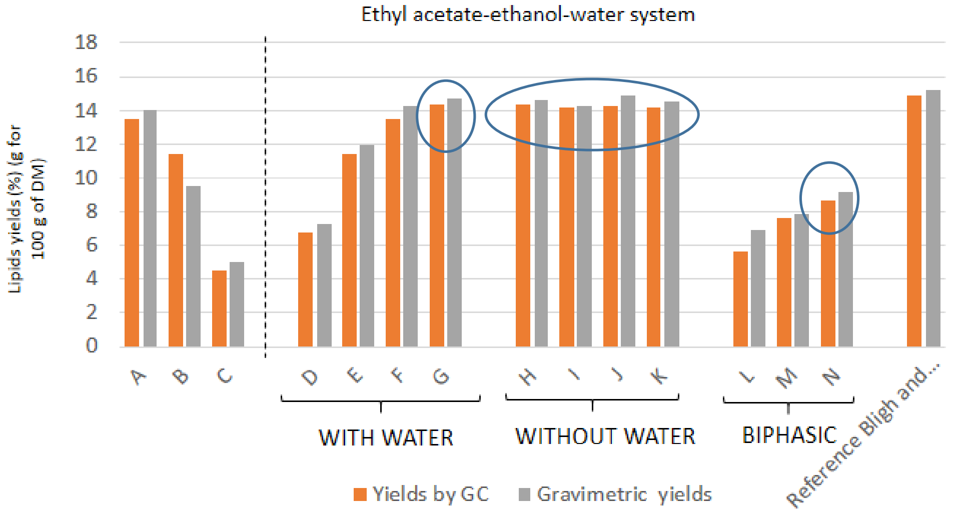

Theory is similar with the experiment

CONCLUSION

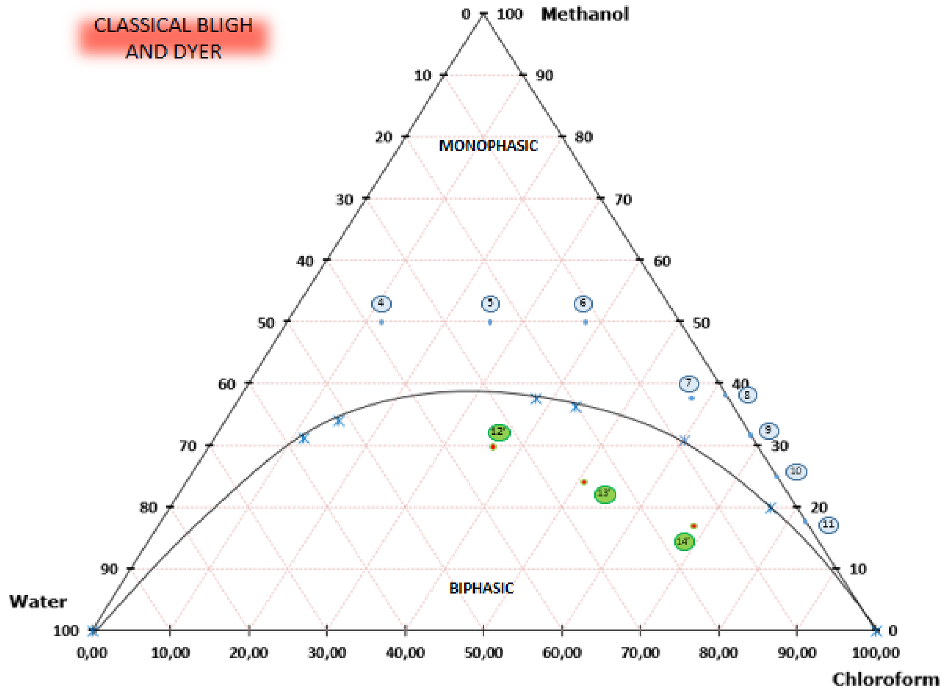

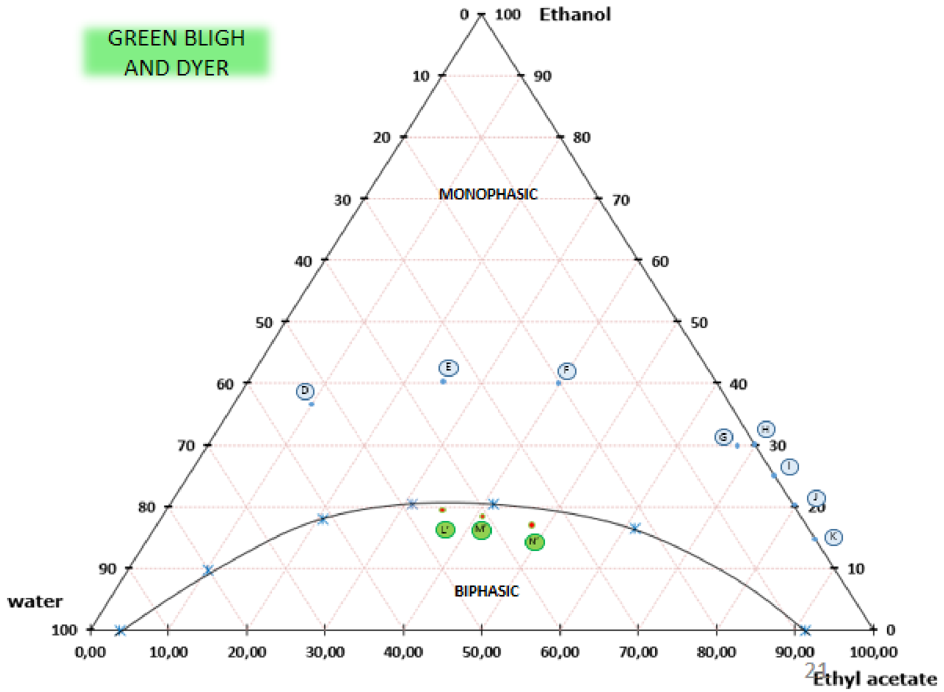

✚

- GREEN bligh and dyer is efficient to extract lipids in organic phase but also proteins and sugars in aqueous phase
- The % of ethyl acetate to point E is sufficient to extract all lipids

THEORITICAL APPROACH

- Theory given with COSMO-RS matches with experiment
